# Cascade Aryne Aminoarylation for Biaryl Phenol Synthesis

**DOI:** 10.1021/acs.orglett.4c00624

**Published:** 2024-03-21

**Authors:** Aniruddha Das, Danielle L. Myers, Venkataraman Ganesh, Michael F. Greaney

**Affiliations:** † Department of Chemistry, University of Manchester, Oxford Rd, Manchester, M13 9PL, U.K.; ‡ Department of Chemistry, Indian Institute of Technology Kharagpur, West Bengal-721302, India

## Abstract

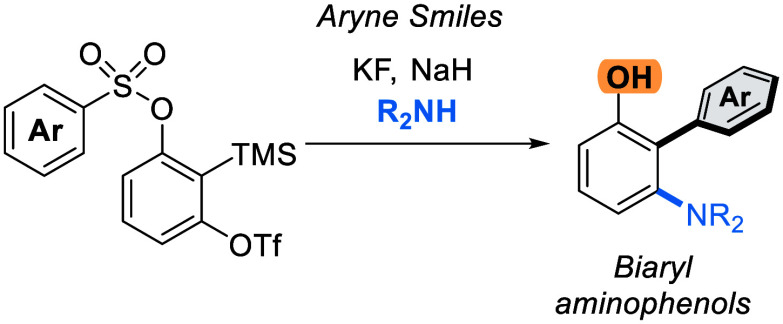

We describe a transition metal-free approach to hindered 3-amino-2-aryl phenols through a cascade nucleophilic addition / Smiles–Truce rearrangement of a functionalized Kobayashi aryne precursor. Under anionic conditions, secondary alkyl amines add to the aryne intermediate to set up an aryl transfer from a neighboring sulfonate group. The use of a sulfonate, rather than the more typical sulfonamide, enables access to phenolic biaryl products that are important motifs in natural products and pharmaceuticals.

The biaryl structure is a fundamental architecture found in many soft materials, pharmaceuticals, and natural products. As a result, numerous methods have been developed to synthesize these motifs, with transition metal-catalyzed cross-couplings being especially prominent in recent years.^1^ The aryne route to biaryl synthesis offers a different approach, creating biaryl bonds under conditions that can be mild and economical for certain substrate classes.^2,3^ The aryne route was first described in seminal work from Wittig via the addition of phenyllithium to benzyne (**1**) to yield biphenyl (**3**) (Scheme 1A), with early examples generally featuring strongly basic conditions that limited application to more complex products.^4^ The introduction of Kobayashi precursors (**5**) has prompted a re-examination of the aryne route, as the benzyne-generating method is extremely mild.^5^ A number of metal-free arylation strategies are now possible, often via initial pericyclic or heteroatom addition to the aryne, followed by biaryl C–C bond formation.^6^ An example from our laboratory demonstrated aryne capture by electron-poor arylsulfonamides **4** to set up a Smiles–Truce rearrangement,^6d^ eliminating SO_2_ to form aminobiaryls **7** (Scheme 1B).^7^ Such methods enable access to densely functionalized biaryls under transition metal-free conditions, which are attractive for sustainable chemical synthesis.

**Scheme 1:**
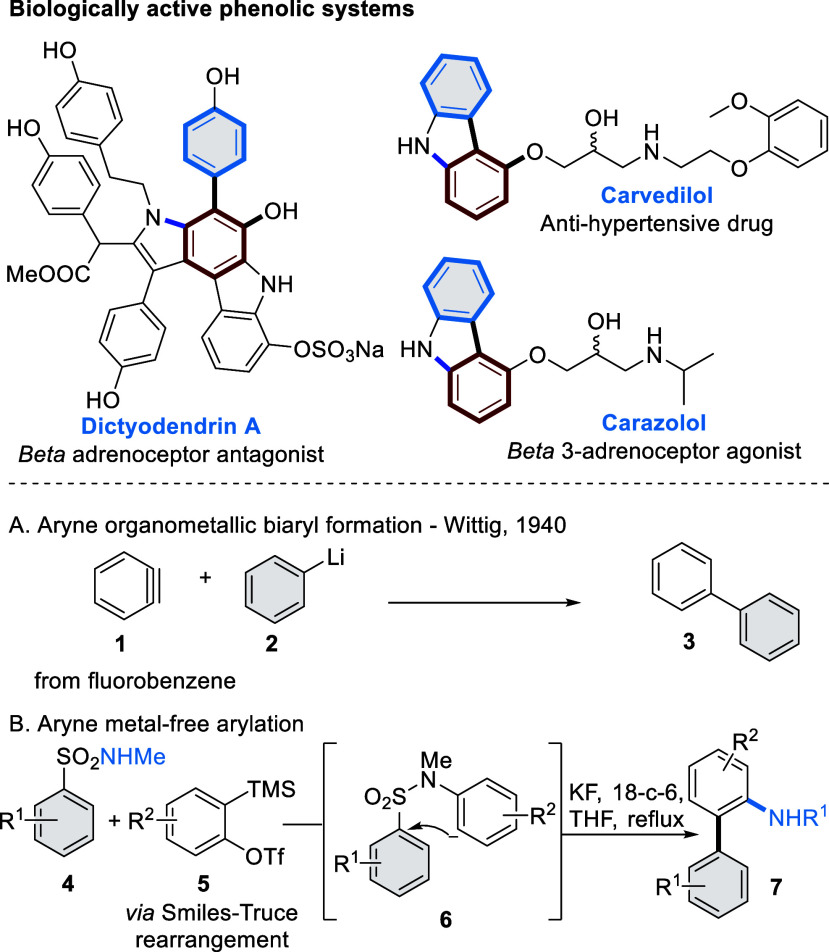
Aryne Approaches to Biaryls

We were interested in applying the Smiles–Truce aryne approach to biaryl phenol synthesis, as these motifs are widely found in biologically active compounds such as dictyodendrin A and B, carvedilol, and carazolol (Scheme 1). Initial attempts at the addition/rearrangement of arylsulfonic acids to benzyne, analogous to our sulfonamide system (Scheme 1B), were unsuccessful, prompting us to consider a new reaction design. Accordingly, we designed sulfonate aryne precursor **8**, with the aim of setting up a cascade process of regioselective nucleophilic addition,^8^ triggering a Smiles–Truce aryl transfer and resultant biaryl formation (Scheme 2A). Work from Li, Hosoya, Yoshida, Akai, and others has shown that designing more functionalized Kobayashi precursors can enable powerful cascade sequences, rapidly accessing arenes that are typically made via lengthy, multistep routes.^9^

**Scheme 2:**
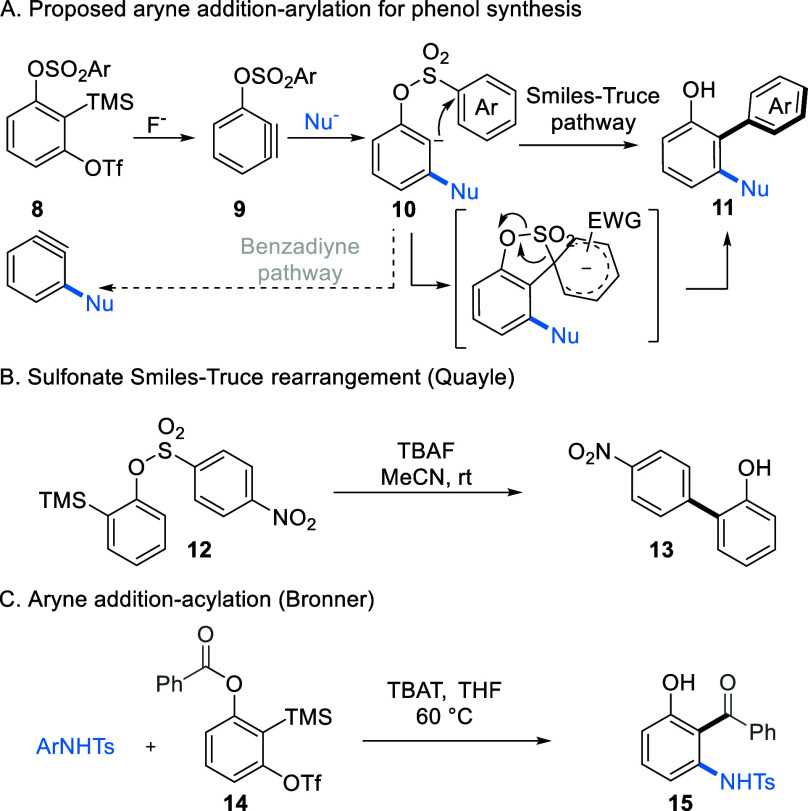
Substrate Design and Prior Art

We envisaged that the addition of a heteroatom nucleophile, such as an amine, to aryne **9** would generate anion **10**, which could undergo arene transfer to give phenol product **11** and establish a transition metal-free route to this important class of biaryls (Scheme 2A). The proposed route requires that anion intermediate **10** undergo the desired Smiles–Truce rearrangement and not participate in an elimination reaction, creating a second benzyne intermediate. Li and co-workers have extensively developed this benzadiyne chemistry using triflate leaving groups,^10^ making the choice of arylsulfonate key to controlling the desired arylation pathway. A further consideration for the proposed transformation concerns the critical SO_2_ extrusion step from a sulfonate linkage. While arylsulfonamides have been central to the renaissance of Smiles–Truce methods in synthesis, the related sulfonates are far less common. Studying the more fragile O–S bond in this context as a precursor to phenolic products would be interesting.^11^ An important precedent for sulfonate Smiles–Truce chemistry in the anionic regime has been reported by Quayle (Scheme 2B), who showed that silyl sulfonates **12** effectively undergo aryl transfer when treated with fluoride.^12^ Further relevant prior art for the proposed aryne reactivity came from Bronner and co-workers, who described an aryne nucleophilic addition–Fries rearrangement system to access C-acylated amino-phenols **15** using sulfonamides as nucleophiles (Scheme 2C).^13^

We began our studies using nosylate **8a**, containing an electron-deficient arene, to promote the planned Smiles–Truce rearrangement. Sulfonate **8a** can be prepared in three steps from a symmetrical resorcinol starting material, using a procedure modified from that of Hosoya and co-workers (see the Supporting Information).^14^ Using KF in the presence of 18-crown-6 as the fluoride source and THF as the solvent, we trialed diisopropylamine (DIPA) as a nucleophile at 70 °C to promote the aryl transfer. We were pleased to observe the formation of the desired biaryl phenol **11a**, but in low yield (25%) and accompanied by slightly larger amounts of simple addition product **16**.

We did not observe nosylate elimination [from intermediate **10** (Scheme 2)], suggesting that a putative benzadiyne pathway was not operational under these conditions. The formation of **16** presumably resulted from simple protonation of intermediate **10** (Scheme 2A). To suppress this side product, we switched to anionic conditions using NaH to deprotonate the amine before it attacks the benzyne and were pleased to isolate **11a** as the desired product (40%) with only small amounts of **16** [5% (Table 1, entry 3)]. Further increasing the equivalency of NaH completely suppressed the formation of **16** (entry 4).

**Table 1:**
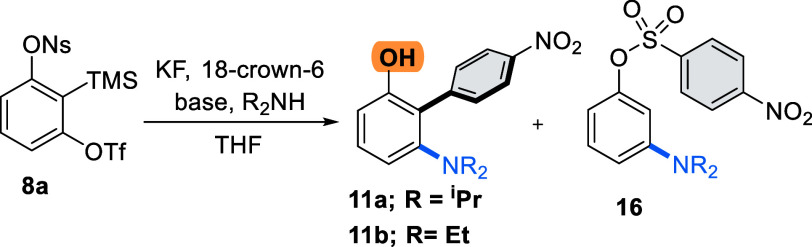
Optimization of the Reaction Conditions for the Smiles–Truce Rearrangement

entry^a^	nucleophile	temp (°C)	time (h)	base (equiv)	yield (%) (**11a/11b**)	yield (%) (**16**)
1^b^	DIPA	70	1	–	25	35
2	DIPA	70	1	–	31	32
3	DIPA	70	1	NaH (4)	40	5
4	DIPA	70	1	NaH (6)	42	0
5	Et_2_NH	70	1	NaH (6)	45	0
6	Et_2_NH	70	16	NaH (6)	35	0
7	Et_2_NH	rt	16	NaH (6)	52	0
8^c^	Et_2_NH	rt	16	NaH (6)	64	0

a NMR yields with nitromethane as the internal standard. Precursor **8a** (0.1 mmol), amine (0.4 mmol), KF (0.2 mmol), and 18-crown-6 (0.3 mmol) at a concentration of 6.3 mM in THF. Ns = *p*-nitrophenylsulfonyl. DIPA = diisopropylamine.

b Reaction run at 0.031 M.

c KF (3 equiv), 18-crown-6 (3 equiv).

Finally, when Et_2_NH was employed as the nucleophile, switching to room-temperature conditions for a longer reaction time of 16 h proved beneficial, affording a 52% yield of biaryl product **11b**. The yield of **11b** further improved to 64% with additional fluoride loading (entry 8).

With the optimized conditions in hand, we examined the reaction with different amine nucleophiles and migratory groups (Scheme 3). We were pleased to observe the biaryl products with a wide range of acyclic secondary amines (**11a**–**g**) and cyclic secondary amines (**11h**–**l**) as pronucleophiles, including the highly sterically hindered examples, diisopropylamine **11a** (46%) and dicyclohexylamine **11d** (32%). Primary amines were ineffective in the reaction because the residual proton on the amine after the nucleophilic attack on the benzyne effectively sequstered the resultant aryl anion, affording low yields of the products (<5%). Pleasingly, with benzylated secondary amines as pronucleophiles, the reaction provided the desired biaryl product **11e** in good yield (70%), which acts as a surrogate for primary amines upon debenzylation. Similarly, dibenzylamine afforded **11f** in good yield (75%), providing a route to installing the -NH_2_ group. We were pleased to observe reactivity for cyclic amine substrates such as piperidines (**11h**–**j**), azepane **11k**, and perhydroisoquinoline **11l** in moderate yields (32–55%). These *N*-arylamines are important pharmacophores, with the *N*-arylpiperidine, in particular, being extensively represented in pharmaceutical structures.^15^

**Scheme 3:**
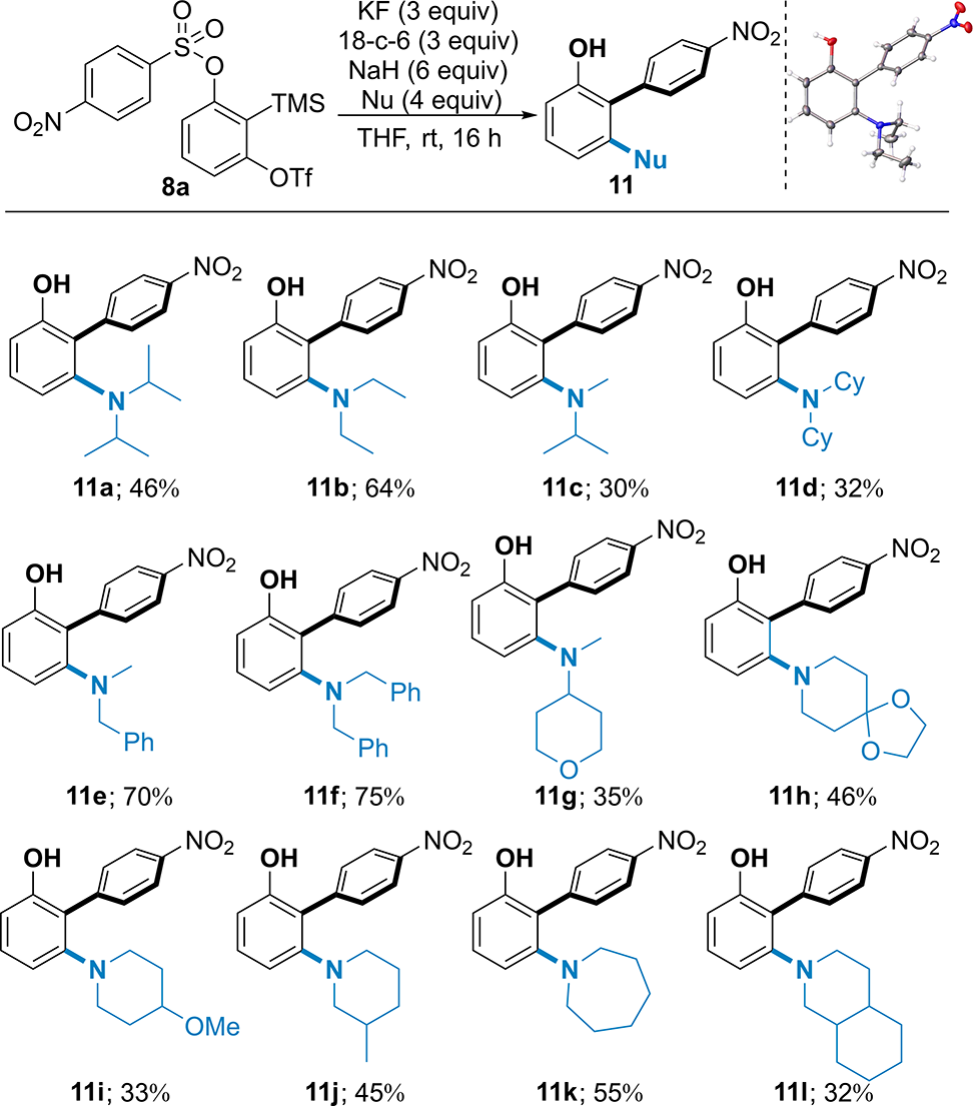
Substrate Scope for Nucleophiles

Next, we examined the scope of the substituents on the migratory arene ring (Scheme 4). Electron-poor arenes are frequently a requisite of anionic Smiles–Truce rearrangements, and that proved to be the case for this reaction. Using diethylamine as the nucleophile, we could situate the activating NO_2_ group at the *ortho* position, affording biaryls **11m**–**p** containing three substituents around the biaryl axis. Additional *m*-chloro (**11o**) and *p*-chloro and methoxy (**11n** and **11p**, respectively) substitution on the *o*-nitro-substituted ring afforded good yields (46–61%). We found that the *o*-methoxy-*p*-nitro arene was the most effective substitution pattern in the study, affording product **11q** with diethylamine in excellent yield (85%). With DIPA as a pronucleophile, the reaction afforded the corresponding biaryl product **11r** in 50% yield.

**Scheme 4:**
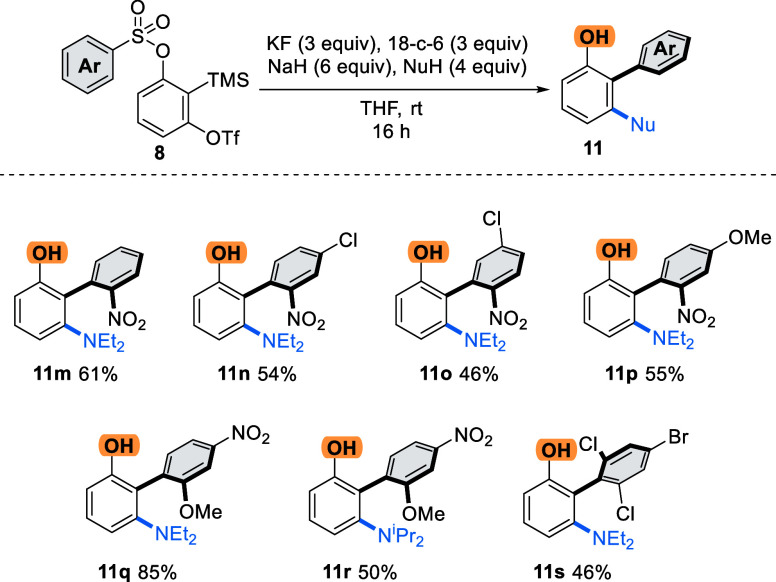
Substrate Scope for the Migratory Aryl Group

Other less electron-deficient arenes were not generally successful migrators in the reaction, but we were pleased to observe reactivity with the 2,4,5-trihaloarene derivative, affording tetra-*o*-substituted biaryl phenol **11s** in 46% yield. Such biaryl structures are frequently challenging to synthesize using transition metal catalysis, due to the highly hindered C–C bond surrounded by multiple metal-coordinating groups.

The versatility of the nitro group in subsequent manipulations enables a switch from the electron-poor arene character to electron-rich character via simple reduction (Scheme 5). We exemplified this reaction with substrate **11b** using iron, preparing aniline **17** in good yield (74%). We could also exploit the facility of the nitro group to act as a nucleofuge in ipso substitution, whereby treatment of **11n** with NaH gave functionalized dibenzofuran **18** in 68% yield.

**Scheme 5:**
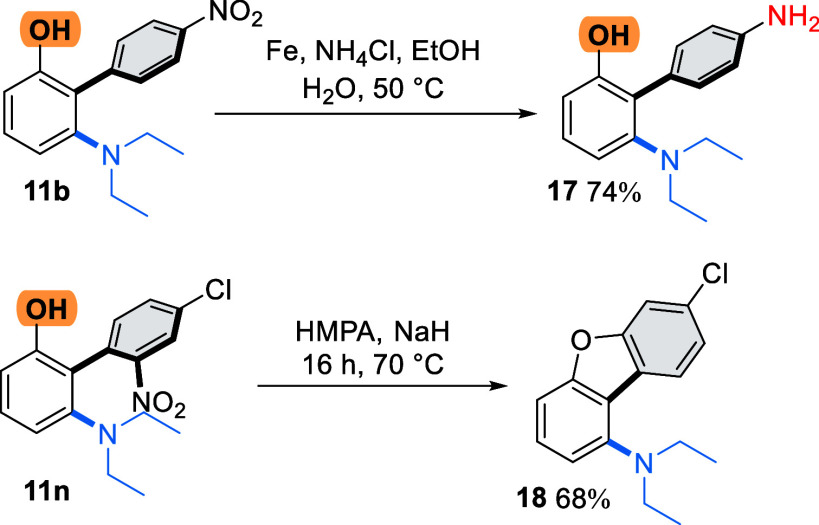
Synthetic Utility of Biaryl Phenols

In conclusion, we have developed a new cascade transformation built around Kobayashi precursors featuring aryl sulfonate groups adjacent to the putative triple bond. The process creates hindered biaryl aminophenols through the nucleophilic addition of secondary amines, followed by Smiles–Truce rearrangement of the sulfonate group. The reaction is free of transition metals, using simple base treatment at room temperature to create the aryl C–C bond, and is amenable to the construction of highly sterically hindered biaryls.

## Data Availability

The data underlying this study are available in the published article and its Supporting Information.

## Supporting Information Available

Synthetic methods, optimization studies, preparative procedures, mechanistic studies, ^1^H, ^13^C, and ^19^F NMR spectra, HRMS, and melting points (PDF)
