# Supplementary material for: Cascade Aryne Aminoarylation for Biaryl Phenol Synthesis
Source: Org Lett. 2024 Mar 21;26(13):2612–6. doi: 10.1021/acs.orglett.4c00624 (PMC11002935; doi:10.1021/acs.orglett.4c00624)
Supplement: Supplementary file 1 — ol4c00624_si_001.pdf [file ol4c00624_si_001.pdf]

Cascade Aryne Aminoarylation for Biaryl Phenol Synthesis

Aniruddha Das, Danielle L. Myers, Venkataraman Ganesh,* Michael F. Greaney*

[a] Department of Chemistry, University of Manchester, Oxford Road, Manchester, M139PL, UK

[b] Department of Chemistry, Indian Institute of Technology Kharagpur-721302, West Bengal, India

ganesh.v@chem.iitkgp.ac.in; michael.greaney@manchester.ac.uk

Supporting Information

1. General Remarks S2
2. General Procedures S3
3. Unsuccessful Substrates S5
4. Data for Synthesized Compounds S5
5. Single crystal XRD Data for **11b** S21
6. NMR Spectra for Synthesized Compounds S24
7. References S55

1. General Remarks

All solvents and reagents were purchased from Sigma Aldrich, Thermo Fisher Scientific, Apollo Scientific or Fluorochem and were used as received without further purification. Flash column chromatography was performed using either Biotage Snap Ultra cartridges or Biotage Sfar Silica cartridges on a Biotage Isolera automated column. ^{1}H, ^{13}C and ^{19}F NMR spectroscopy were recorded on either 400 MHz or 500 MHz Bruker Avance NMR spectrometers. Chemical shifts (δ) are reported in parts per million (ppm) and multiplicities are reported as either singlets (s), doublets (d), triplets (t), quartets (q) or multiplets (m). Coupling constants (J) are reported in Hertz (Hz). All ^{1}H NMR and ^{13}C NMR shifts were referenced to the residual solvent peak of $CDCl_3$ (^{1}H referenced to 7.26 ppm and ^{13}C referenced to 77.16 ppm. All ^{19}F chemical shifts were unadjusted from raw data. High resolution mass spectrometry (HRMS) was recorded on a Waters QTOF, using either ESI or APCI as ionisation methods. Thin layer chromatography (TLC) was carried out using commercially available coated TLC plates and spots were illuminated either by UV light (254 nm) or by staining the plate with a $KMnO_4$ solution. Compound names are those generated by ChemBioDrawTM (CambridgeSoft) following International Union of Pure and Applied Chemistry (IUPAC) nomenclature. Melting points (MPs) were recorded on a Griffin melting point apparatus to the nearest degree. Reactions which proceeded under microwave irradiation were performed in a Biotage Initiator Microwave Synthesizer. Reactions which performed under heating conditions silicone oil bath was used for heating. Low temperature reactions were performed using dry ice.

2. General Procedures

General Procedure A for the synthesis of starting materials

2-(Trimethylsilyl)-3-(trimethylsilyloxy) phenol (C)

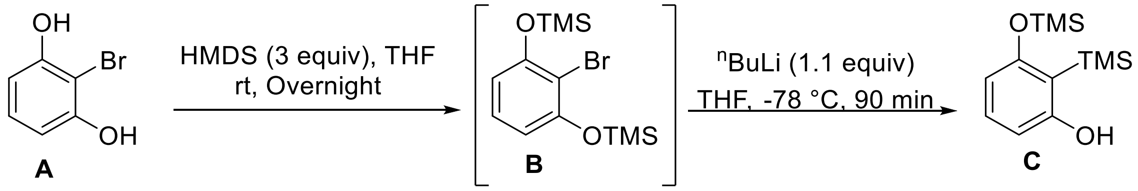

Prepared according to a procedure by Hosoya and co-workers.[1] A round bottomed flask was charged with 2-bromoresorcinol **A** (3.18 g, 16.8 mmol, 1.00 equiv) and a stirrer bar, and evacuated/backfilled with N_2 × 3. To this reaction mixture was added dry THF (6.7 mL), followed by HMDS (10.6 mL, 8.13 g, 50.4 mmol, 3.00 equiv). The reaction mixture was left stirring at room temperature overnight. The reaction mixture was then concentrated in vacuo. The resulting residue was dissolved in dry THF (34 mL) under N_2, and cooled to -78 ^{o}C. To this was added ^{n}BuLi (1.60 M in hexanes, 11.6 mL, 18.5 mmol, 1.10 equiv) dropwise. The reaction mixture was stirred for 1.5 hours at -78 ^{o}C, then sat. aq. NH_4Cl (35 mL) was added. The layers were separated, and the aqueous layer was extracted with DCM (25 mL × 2). The combined organic extracts were washed with brine (25 mL), dried over Na_2SO_4, and concentrated in vacuo. The resulting crude product was purified by column chromatography.

3-Hydroxy-2-(trimethylsilyl)phenyl triflate (F)

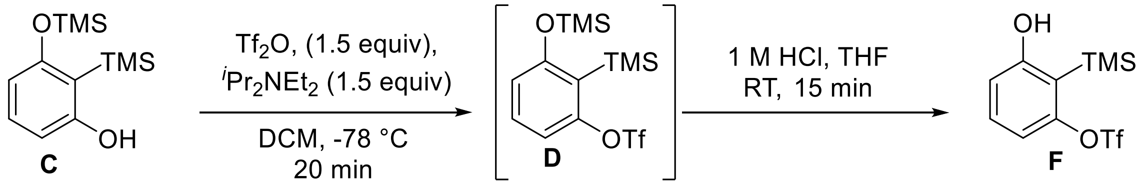

Prepared according to a procedure by Hosoya and co-workers.[1] To a stirring solution of 2-(trimethylsilyl)-3-(trimethylsilyloxy)phenol **C** (2.98 g, 11.7 mmol, 1.00 equiv) in dry DCM under N_2 was added $^{i}Pr_2NEt$ (3.06 mL, 2.27 g, 17.6 mmol, 1.50 equiv). The reaction mixture was cooled to -78 ^{o}C, and triflic anyhydride (2.96 mL, 4.96 g, 17.6 mmol, 1.50 equiv) was added dropwise. The reaction mixture was stirred for 20 minutes at -78 ^{o}C, then sat. aq. $NaHCO_3$ (35 mL) was added, and the mixture was extracted with DCM (25 mL×3). The combined organic extracts were washed with brine (25 mL), dried over Na_2SO_4, and concentrated to give crude 2-(trimethylsilyl)-3- ((trimethylsilyl)oxy)phenyl triflate **D**, which was used without further purification. 1.0 M aq. HCl (11.7 mL, 11.7 mmol, 1.00 equiv) was added to a stirring solution of 2-(trimethylsilyl)-3-((trimethylsilyl)oxy)phenyl triflate **D** (611.7 mmol, 1.00 equiv) in THF (11.7 mL). After 15 minutes at room temperature, the reaction was quenched with sat. aq. $NaHCO_3$ (7 mL). The mixture was extracted with EtOAc (25 mL × 3), and the combined organic extracts were washed with

brine (20 mL), dried over Na_2SO_4 and concentrated. The resulting oil was purified by column chromatography.

3-(((Triuoromethyl)sulfonyl)oxy)-2-(trimethylsilyl)phenyl 4-nitrobenzene-sulfonate (8)

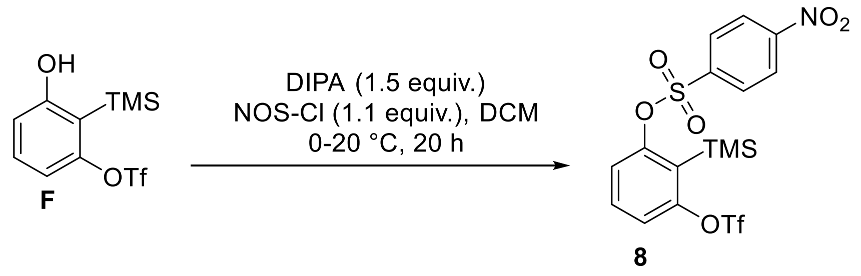

A stirring solution of 3-hydroxy-2-(trimethylsilyl)phenyl triflate, **F** (1.04 g, 3.31 mmol, 1.00 equiv) in dry DCM (61 mL) was cooled to 0 ºC. To this was added DIPA (0.696 mL, 502 mg, 4.96 mmol, 1.50 equiv). After five minutes at 0 ºC, 4-nitrobenzenesulfonyl chloride (807 mg, 3.64 mmol, 1.10 equiv) was added. The reaction mixture was stirred for a further 20 minutes at 0 ºC, before being allowed to warm to room temperature overnight. After 20 hours, sat. aq. NH_4Cl was added to the reaction mixture. The layers were separated, and the aqueous layer was extracted twice with DCM. The combined organic extracts were washed with brine, dried over Na_2SO_4, and concentrated.

General procedure B for the desulfonylative Smiles rearrangement

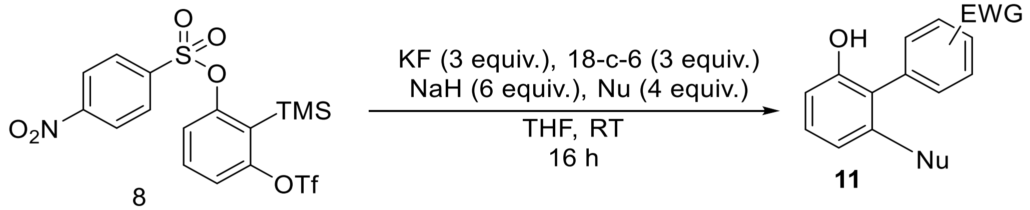

A microwave vial was charged with a stirrer bar, NaH (0.600 mmol, 6 equiv), KF (0.300 mmol, 3.00 equiv), and 18-crown-6 (0.300 mmol, 3.00 equiv), then capped and evacuated/backfilled with $N_2 \times 3$. To this was added nucleophile (0.400 mmol, 4.00 equiv) and THF (14.4 mL). The reaction mixture was stirred at room temperature for 20 min, before the addition of a solution of [3-(triuoromethylsulfonyloxy-2-trimethylsilyl-phenyl] 4-nitrobenzenesulfonate **8** (50.0 mg, 0.100 mmol, 1.00 equiv) in THF (1.6 mL). The reaction mixture was left stirring in a sealed vial at room temperature for 16 hours. The reaction mixture was diluted with Et_2O, and H_2O was added. The pH was adjusted to between 6 and 7 using 1 M aq. HCl, and the layers were separated, then the organic layer was washed with brine (2 × 2 mL). The organic layer was dried over Na_2SO_4, then the solvent removed in vacuo. The yield was calculated using ^{1}H NMR, with nitromethane as an internal standard.

3. Unsuccessful substrates

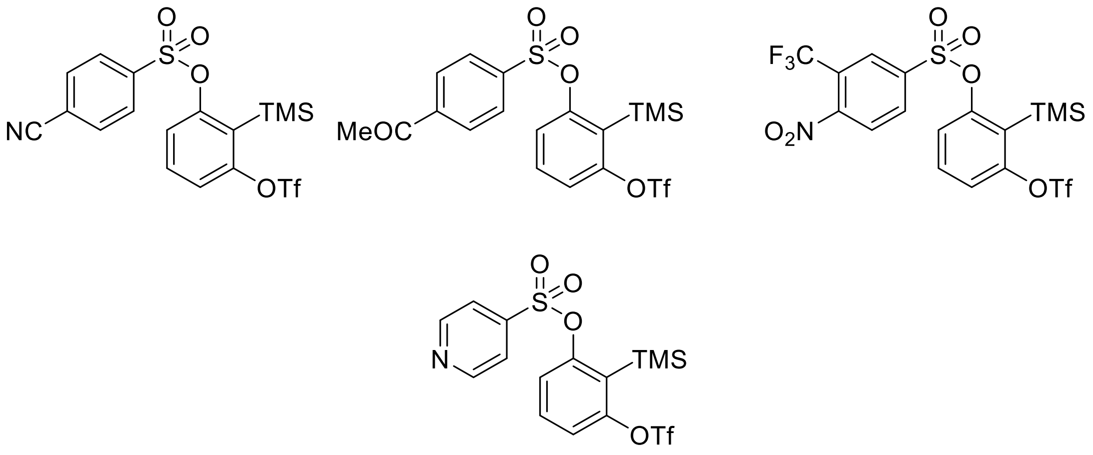

4. Data for synthesized compounds

Synthesized starting materials

2-(Trimethylsilyl)-3-(trimethylsilyloxy) phenol (C)

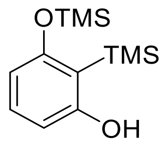

Synthesized according to the general procedure A (column condition: 0-50% EtOAc in hexane). The pure product was afforded as a white solid (2.98 g, 70% yield).

^{1}H NMR (400 MHz, Chloroform-*d*) δ 7.08 (t, *J* = 8.0 Hz, 1H), 6.35 (m, 2H), 5.06 (d, *J* = 1.3 Hz, 1H), 0.36 (s, 9H), 0.32 (s, 9H).

Data matched with reported literature compound.[2]

3-Hydroxy-2-(trimethylsilyl)phenyl triflate (F)

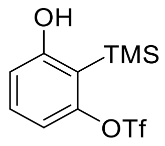

Synthesized according to the general procedure A (column condition: 0-50% EtOAc in hexane). The pure product was afforded as a yellow oil (1.86 g, 50% yield).

^{1}H NMR (400 MHz, Chloroform-*d*) δ 7.26 (t, *J* = 8.2 Hz, 1H), 6.93 (d, *J* = 8.3 Hz, 1H), 6.69 (d, *J* = 8.1 Hz, 1H), 0.42 (s, 9H).

Data matched with reported literature compound.[2]

3-(((trifluoromethyl)sulfonyl)oxy)-2-(trimethylsilyl)phenyl 4-nitrobenzenesulfonate (8a)

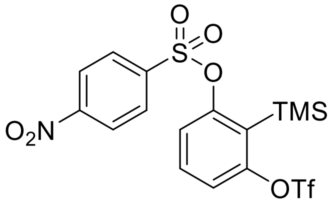

Synthesized according to the general procedure A (column condition: 0-50% EtOAc in hexane). The pure product was afforded as a yellow solid (180 mg, 72% yield). Data matched with the reported literature compound.[3]

^{1}H NMR (400 MHz, Chloroform-*d*) δ 8.44 (d, *J* = 8.3 Hz, 2H), 8.12 (d, *J* = 8.4 Hz, 2H), 7.39 (t, *J* = 8.3 Hz, 1H), 7.31 (d, *J* = 8.4 Hz, 1H), 7.05 (d, *J* = 8.2 Hz, 1H), 0.36 (s, 9H).

3-(((trifluoromethyl)sulfonyl)oxy)-2-(trimethylsilyl)phenyl 2-nitrobenzenesulfonate (8b)

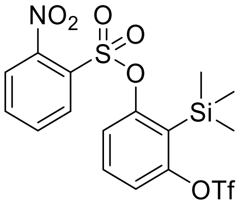

Synthesized according to the general procedure A (column condition: 0-50% EtOAc in hexane). The pure product was afforded as a yellow solid (145 mg, 58% yield).

^{1}H NMR (500 MHz, Chloroform-*d*) δ 8.07 (d, *J* = 7.9 Hz, 1H), 7.91 (d, *J* = 4.1 Hz, 2H), 7.80 (m, 1H), 7.37 (t, *J* = 8.2 Hz, 1H), 7.32 (d, *J* = 9.5 Hz, 1H), 7.02 (d, *J* = 8.0 Hz, 1H), 0.43 (s, 9H).

^{13}C NMR (126 MHz, Chloroform-*d*) δ 155.3, 154.9, 137.1, 136.3, 133.4, 132.9, 132.0, 132.0, 129.5, 127.4, 125.7, 120.9, 116.3 (q, *J* = 309.6 Hz), 1.1.

^{19}F NMR (471 MHz, Chloroform-*d*) δ -73.36.

HRMS (ESI) m/z: $[M+Na]^+$calcd for $C_{16}H_{16}F_3NO_8S_2SiNa$: 521.9931. Found: 521.9917

MP 52-54 ºC

3-(((trifluoromethyl)sulfonyl)oxy)-2-(trimethylsilyl)phenyl 4-chloro-2-nitrobenzenesulfonate (8c)

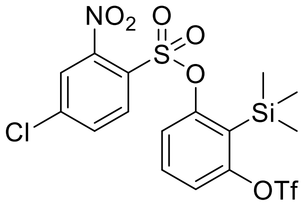

Synthesized according to the general procedure A (column condition: 0-50% EtOAc in hexane). The pure product was afforded as a yellow solid (149 mg, 56% yield).

^{1}H NMR (500 MHz, Chloroform-*d*) δ 8.00 (d, *J* = 8.5 Hz, 1H), 7.89 (d, *J* = 2.1 Hz, 1H), 7.75 (dd, *J* = 8.5, 2.0 Hz, 1H), 7.38 (t, *J* = 8.2 Hz, 1H), 7.33 (d, *J* = 8.4 Hz, 1H), 7.01 (dd, *J* = 8.1, 1.1 Hz, 1H), 0.42 (s, 9H).

^{13}C NMR (126 MHz, Chloroform-*d*) δ 155.4, 154.8, 149.2, 142.7, 133.3, 132.9, 132.1, 127.9, 127.5, 125.9, 120.9, 119.3, 117.5 (q, *J* = 317.5 Hz), 1.1.

^{19}F NMR (471 MHz, Chloroform-*d*) δ -73.38.

HRMS (ESI) m/z: $[M+Na]^+$calcd for $C_{16}H_{15}ClF_3NO_8S_2SiNa$: 555.9541. Found: 555.9538

MP 78-80 ºC

3-(((trifluoromethyl)sulfonyl)oxy)-2-(trimethylsilyl)phenyl 5-chloro-2-nitrobenzenesulfonate (8d)

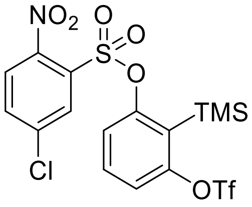

Synthesized according to the general procedure A (column condition: 0-50% EtOAc in hexane). The pure product was afforded as a yellow solid (139 mg, 52% yield).

^{1}H NMR (500 MHz, Chloroform-*d*) δ 8.06 (d, *J* = 2.2 Hz, 1H), 7.91 (d, *J* = 8.5 Hz, 1H), 7.85 (dd, *J* = 8.6, 2.2 Hz, 1H), 7.41 (t, *J* = 8.3 Hz, 1H), 7.35 (d, *J* = 8.4 Hz, 1H), 7.04 (dd, *J* = 8.1, 1.0 Hz, 1H), 0.44 (s, 9H).

^{13}C NMR (126 MHz, Chloroform-*d*) δ 155.2, 154.5, 146.7, 139.3, 135.7, 131.9, 131.7, 131.2, 127.2, 126.9, 120.6, 119.1, 116.1 (q, *J* = 319.5 Hz), 0.9.

^{19}F NMR (471 MHz, Chloroform-*d*) δ -73.34.

HRMS (ESI) m/z: $[M+Na]^+$calcd for $C_{16}H_{15}ClF_3NO_8S_2SiNa$: 555.9541. Found:555.9538

MP 118-120 ºC

3-(((trifluoromethyl)sulfonyl)oxy)-2-(trimethylsilyl)phenyl-4-methoxy-2-nitrobenzenesulfonate (8e)

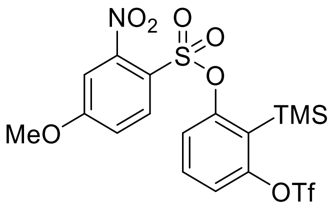

Synthesized according to the general procedure A (column condition: 0-50% EtOAc in hexane). The pure product was afforded as a yellow solid (169 mg, 64% yield).

^{1}H NMR (500 MHz, Chloroform-*d*) δ 7.94 (d, *J* = 8.8 Hz, 1H), 7.37 (d, *J* = 8.3 Hz, 1H), 7.35 (d, *J* = 2.7 Hz, 1H), 7.30 (d, *J* = 8.4 Hz, 1H), 7.17 (d, *J* = 2.6 Hz, 1H), 7.06 (d, *J* = 7.2 Hz, 1H), 3.98 (s, 3H), 0.42 (s, 9H).

^{13}C NMR (126 MHz, Chloroform-*d*) δ 164.9, 155.1, 154.8, 150.3, 133.9, 131.6, 127.1, 120.8, 120.4, 118.7, 118.6 (q, *J* = 320.4 Hz), 116.6, 111.6, 56.8, 0.9.

^{19}F NMR (471 MHz, Chloroform-*d*) δ -73.39.

HRMS (ESI) m/z: $[M+Na]^+$ calcd for $C_{17}H_{18}F_3NO_9S_2SiNa$: 552.0037. Found:552.0060

MP 87-89 ^oC

3-(((trifluoromethyl)sulfonyl)oxy)-2-(trimethylsilyl)phenyl 2-methoxy-4-nitrobenzenesulfonate (8f)

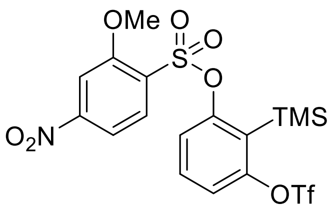

Synthesized according to the general procedure A (column condition: 0-50% EtOAc in hexane). The pure product was afforded as an white solid (164 mg, 62% yield).

^{1}H NMR (500 MHz, Chloroform-*d*) δ 8.16 (d, *J* = 8.6 Hz, 1H), 7.96 (s, 1H), 7.94 (d, *J* = 7.0 Hz, 1H), 7.39 (t, *J* = 8.3 Hz, 1H), 7.28 (d, *J* = 8.5 Hz, 1H), 7.12 (d, *J* = 8.2 Hz, 1H), 4.03 (s, 3H), 0.38 (s, 9H).

^{13}C NMR (126 MHz, Chloroform-*d*) δ 158.7, 155.6, 155.1, 153.0, 132.8, 131.9, 130.2, 126.6, 120.1, 118.8, 118.2 (q, *J* = 320.6 Hz), 115.5, 108.3, 57.6, 1.1.

^{19}F NMR (471 MHz, Chloroform-*d*) δ -73.23.

HRMS (ESI) m/z: $[M+Na]^+$ calcd for $C_{17}H_{18}F_3NO_9S_2SiNa$: 552.0036. Found:552.0037

MP 87-89 ^oC

3-(((trifluoromethyl)sulfonyl)oxy)-2-(trimethylsilyl)phenyl 4-bromo-2,6-dichlorobenzenesulfonate (8g)

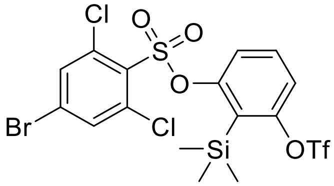

Synthesized according to the general procedure A (column condition: 0-50% EtOAc in hexane). The pure product was afforded as a off white solid (214 mg, 71% yield).

^{1}H NMR (500 MHz, Chloroform-*d*) δ 7.56 (s, 2H), 7.21 (t, *J* = 8.3 Hz, 1H), 7.13 (d, *J* = 8.4 Hz, 1H), 6.76 (d, *J* = 8.2 Hz, 1H), 0.28 (s, 9H).

^{13}C NMR (126 MHz, Chloroform-*d*) δ 155.0, 154.9, 136.8, 134.5, 132.4, 131.8, 128.5, 126.9, 119.5, 118.7, 118.6 (q, *J* = 320.4 Hz), 0.9.

^{19}F NMR (471 MHz, Chloroform-*d*) δ -73.22.

HRMS (ESI) m/z: $[M+Na]^+$calcd for $C_{16}H_{14}BrCl_2F_3O_6S_2SiNa$: 622.8406. Found: 622.8398

MP 56-58 ^oC

Products from desulfonylative Smiles rearrangement

6-(diisopropylamino)-4'-nitro-[1,1'-biphenyl]-2-ol (11a)

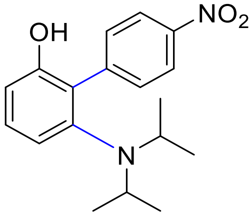

Synthesized according to the general procedure B (column condition: 0-50% EtOAc in hexane). The pure product was afforded as a yellow solid (15 mg, 46% yield).

^{1}H NMR (400 MHz, Chloroform-*d*) δ 8.29 (d, *J* = 8.7 Hz, 2H), 7.56 (d, *J* = 8.7 Hz, 2H), 7.21 (t, *J* = 8.1 Hz, 1H), 6.93 (d, *J* = 8.1 Hz, 1H), 6.78 (d, *J* = 8.1 Hz, 1H), 4.67 (s, 1H), 3.41 – 3.17 (m, 2H), 0.89 (d, *J* = 6.5 Hz, 12H).

^{13}C NMR (101 MHz, Chloroform-*d*) δ 153.1, 148.9, 146.9, 143.7, 132.7, 128.8, 127.1, 123.4, 121.0, 112.2, 50.6, 21.7.

HRMS (ESI) m/z: $[M+H]^+$calcd for $C_{18}H_{23}N_2O_3$: 315.1703. Found: 315.1697

MP 136-138 ºC

6-(diethylamino)-4'-nitro-[1,1'-biphenyl]-2-ol (11b)

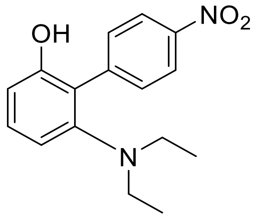

Synthesized according to the general procedure B (column condition: 0-50% EtOAc in hexane). The pure product was afforded as a yellow solid (18 mg, 64% yield).

1 mmol scale procedure:

A microwave vial was charged with a stirrer bar, NaH (6.00 mmol, 6 equiv), KF (3.00 mmol, 3.00 eq.), and 18-crown-6 (3.00 mmol, 3.00 equiv), then capped and evacuated/backfilled with N_2 × 3. To this was added nucleophile (4.00 mmol, 4.00 equiv) and THF (144 mL). The reaction mixture was stirred at room temperature for 20 min, before the addition of a solution of [3-(triuoromethylsulfonyloxy-2-trimethylsilyl-phenyl] 4-nitrobenzenesulfonate **8a** (499 mg, 1.00 mmol, 1.00 equiv) in THF (16 mL). The reaction mixture was left stirring in a sealed vial at room temperature for 16 hours. The reaction mixture was diluted with Et_2O, and H_2O was added. The pH was adjusted to between 6 and 7 using 1 M aq. HCl, and the layers were separated, then the organic layer was washed with brine (2 × 2 mL). The organic layer was dried over Na_2SO_4, then the solvent removed in vacuo. Product was purified using column chromatography (166 mg, 58% yield).

^{1}H NMR (400 MHz, Chloroform-*d*) δ 8.31 (d, *J* = 8.8 Hz, 2H), 7.60 (d, *J* = 8.7 Hz, 2H), 7.21 (t, *J* = 8.1 Hz, 1H), 6.76 (d, *J* = 8.1 Hz, 1H), 6.66 (d, *J* = 8.1 Hz, 1H), 4.69 (s, 1H), 2.77 (q, *J* = 7.1 Hz, 4H), 0.84 (t, *J* = 7.1 Hz, 6H).

^{13}C NMR (126 MHz, Chloroform-*d*) δ 156.7, 153.1, 150.4, 146.8, 143.4, 132.0, 129.4, 123.7, 114.4, 110.3, 46.6, 12.1.

HRMS (ESI) m/z: $[M+H]^+$ calcd for $C_{16}H_{19}N_2O_3$: 287.1390. Found: 287.1388

MP 123-125 ºC

6-(isopropyl(methyl)amino)-4'-nitro-[1,1'-biphenyl]-2-ol (11c)

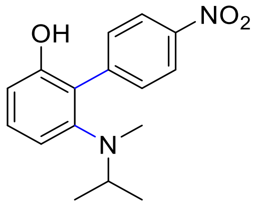

Synthesized according to the general procedure B (column condition: 0-50% EtOAc in hexane). The pure product was afforded as a deep yellow solid (8.5 mg, 30% yield).

^{1}H NMR (400 MHz, Chloroform-*d*) δ 8.31 (d, *J* = 8.8 Hz, 2H), 7.62 (d, *J* = 8.7 Hz, 2H), 7.20 (t, *J* = 8.1 Hz, 1H), 6.73 (d, *J* = 8.5 Hz, 1H), 6.62 (dd, *J* = 8.2, 1.1 Hz, 1H), 4.77 (s, 1H), 3.00 (p, *J* = 6.6 Hz, 1H), 2.50 (s, 3H), 0.73 (d, *J* = 6.6 Hz, 6H).
^{13}C NMR (101 MHz, Chloroform-*d*) δ 152.8, 152.5, 146.5, 143.4, 131.8, 129.4, 123.6, 120.6, 113.5, 109.6, 53.3, 31.8, 17.8.

HRMS (ESI) m/z: $[M+H]^+$ calcd for $C_{16}H_{19}N_2O_3$: 287.1390. Found: 287.1404

MP 155-157 ºC

6-(dicyclohexylamino)-4'-nitro-[1,1'-biphenyl]-2-ol (11d)

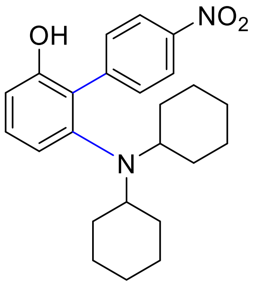

Synthesized according to the general procedure B (column condition: 0-50% EtOAc in hexane). The pure product was afforded as a yellow solid (13 mg, 32% yield).

^{1}H NMR (500 MHz, Chloroform-*d*) δ 8.29 (d, *J* = 8.8 Hz, 2H), 7.55 (d, *J* = 8.7 Hz, 2H), 7.19 (t, *J* = 8.1 Hz, 1H), 6.95 (d, *J* = 8.0 Hz, 1H), 6.77 (d, *J* = 8.1 Hz, 1H), 4.65 (s, 1H), 2.85 (ddq, *J* = 11.2, 6.8, 3.3 Hz, 2H), 1.69–1.57 (m, 9H), 1.56–1.49 (m, 2H), 1.07 (qd, *J* = 10.9, 10.2, 2.7 Hz, 7H), 1.02–0.95 (m, 2H).

^{13}C NMR (126 MHz, Chloroform-*d*) δ 153.1, 149.0, 146.9, 143.9, 132.7, 128.6, 127.4, 123.4, 122.1, 112.1, 60.0, 32.5, 26.5, 26.2.

HRMS (ESI) m/z: $[M+H]^+$ calcd for $C_{24}H_{31}N_2O_3$: 395.2329. Found: 395.2349

MP 156-158 ºC

6-(benzyl(methyl)amino)-4'-nitro-[1,1'-biphenyl]-2-ol (11e)

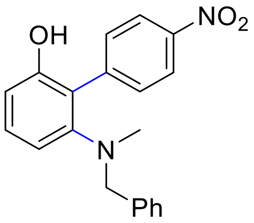

Synthesized according to the general procedure B (column condition: 0-50% EtOAc in hexane). The pure product was afforded as a yellow solid (23 mg, 70% yield).

^{1}H NMR (500 MHz, Chloroform-*d*) δ 8.38 (d, *J* = 8.9 Hz, 2H), 7.72 (d, *J* = 8.4 Hz, 2H), 7.36–7.24 (m, 5H), 7.06–6.98 (m, 2H), 6.86 (d, *J* = 7.6 Hz, 1H), 6.77 (d, *J* = 8.2 Hz, 1H), 4.87 (s, 1H), 3.91 (s, 2H), 2.49 (s, 3H).

^{13}C NMR (101 MHz, Chloroform-*d*) δ 153.3, 152.7, 147.0, 143.2, 138.0, 132.2, 130.1, 128.3, 128.2, 127.2, 124.0, 120.7, 112.9, 110.6, 60.6, 40.7.

HRMS (ESI) m/z: $[M+H]^+$calcd for $C_{20}H_{19}N_2O_3$: 335.1390. Found: 335.1409

MP 100-102 ^oC

6-(dibenzylamino)-4'-nitro-[1,1'-biphenyl]-2-ol (11f)

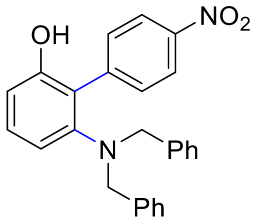

Synthesized according to the general procedure B (column condition: 0-50% EtOAc in hexane). The pure product was afforded as a yellow solid (31 mg, 75% yield).

^{1}H NMR (400 MHz, Chloroform-*d*) δ 8.33 (d, *J* = 8.6 Hz, 2H), 7.51 (d, *J* = 8.6 Hz, 2H), 7.30 – 7.18 (m, 7H), 6.98 (m, 4H), 6.81 (d, *J* = 8.1 Hz, 1H), 6.72 (d, *J* = 8.1 Hz, 1H), 4.81 (s, 1H), 3.85 (s, 4H).

^{13}C NMR (126 MHz, Chloroform-*d*) δ 153.3, 150.7, 147.2, 143.1, 137.8, 132.2, 129.8, 128.9, 128.3, 127.3, 124.0, 122.5, 115.6, 111.4, 57.0.

HRMS (ESI) m/z: $[M+H]^+$ calcd for $C_{26}H_{22}N_2O_3$: 411.1703. Found: 411.1717

MP 143-145 ^oC

6-(methyl(tetrahydro-2H-pyran-4-yl)amino)-4'-nitro-[1,1'-biphenyl]-2-ol (11g)

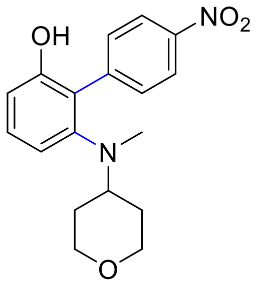

Synthesized according to the general procedure B (column condition: 0-50% EtOAc in hexane). The pure product was afforded as a yellow solid (11 mg, 35% yield).

^{1}H NMR (400 MHz, Chloroform-*d*) δ 8.30 (d, *J* = 8.8 Hz, 2H), 7.60 (d, *J* = 8.8 Hz, 2H), 7.21 (t, *J* = 8.1 Hz, 1H), 6.79 (d, *J* = 1.0 Hz, 1H), 6.66 (d, *J* = 8.1 Hz, 1H), 4.97 (s, 1H), 3.94–3.63 (m, 2H), 3.03 (m, 2H), 2.79 (m, 1H), 2.55 (s, 3H), 1.51 (m, 2H), 1.23–1.03 (m, 2H).

^{13}C NMR (101 MHz, Chloroform-*d*) δ 153.4, 146.9, 143.4, 132.2, 129.8, 126.4, 123.8, 121.9, 114.6, 110.9, 67.6, 60.0, 35.1, 29.4.

HRMS (ESI) m/z: [M-H]$^+$calcd for $C_{18}H_{19}N_2O_4$: 327.1350. Found: 327.1343

MP 179-181 ^{o}C

4'-nitro-6-(1,4-dioxa-8-azaspiro[4.5]decan-8-yl)-[1,1'-biphenyl]-2-ol (11h)

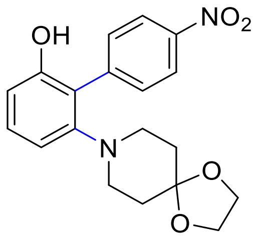

Synthesized according to the general procedure B (column condition: 0-50% EtOAc in hexane). The pure product was afforded as a yellow solid (16 mg, 46% yield).

^{1}H NMR (500 MHz, Chloroform-*d*) δ 8.31 (d, *J* = 8.7 Hz, 2H), 7.69 (d, *J* = 8.7 Hz, 2H), 7.21 (t, *J* = 8.1 Hz, 1H), 6.72 (d, *J* = 8.1 Hz, 1H), 6.67 (d, *J* = 7.6 Hz, 1H), 4.84 (s, 1H), 3.90 (s, 4H), 2.92–2.77 (m, 4H), 1.49 (t, *J* = 5.6 Hz, 4H).

^{13}C NMR (101 MHz, Chloroform-*d*) δ 152.8, 151.9, 146.6, 142.7, 131.6, 129.9, 123.6, 120.2, 111.9, 110.7, 106.5, 64.1, 49.7, 34.8.

HRMS (ESI) m/z: [M-H]$^+$calcd for $C_{19}H_{19}N_2O_5$: 355.1299. Found: 355.1291

MP 198-200 ^{o}C

6-(4-methoxypiperidin-1-yl)-4'-nitro-[1,1'-biphenyl]-2-ol (11i)

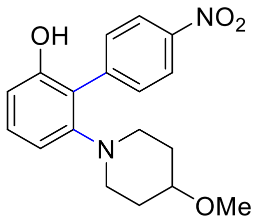

Synthesized according to the general procedure B (column condition: 0-50% EtOAc in hexane). The pure product was afforded as a yellow solid (11 mg, 33% yield).

^{1}H NMR (500 MHz, Chloroform-*d*) δ 8.31 (d, *J* = 8.7 Hz, 2H), 7.68 (d, *J* = 8.7 Hz, 2H), 7.21 (t, *J* = 8.1 Hz, 1H), 6.70 (d, *J* = 8.1 Hz, 1H), 6.66 (d, *J* = 8.1 Hz, 1H), 4.86 (d, *J* = 2.4 Hz, 1H), 3.28 (s, 3H), 3.17 (m, 1H), 2.98–2.89 (m, 2H), 2.57 (m, 2H), 1.72–1.65 (m, 2H), 1.34–1.27 (m, 2H).

^{13}C NMR (101 MHz, Chloroform-*d*) δ 153.1, 152.5, 146.9, 143.0, 131.9, 130.3, 123.9, 120.5, 112.0, 110.9, 55.7, 53.6, 49.6, 31.0.

HRMS (ESI) m/z: $[M-H]^{+}$calcd for $C_{18}H_{19}N_2O_4$: 327.1350. Found: 327.1345

MP 134-136 °C

6-(3-methylpiperidin-1-yl)-4'-nitro-[1,1'-biphenyl]-2-ol (11j)

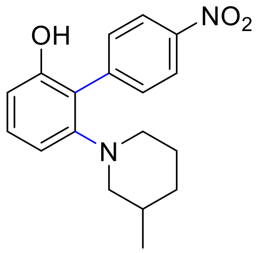

Synthesized according to the general procedure B (column condition: 0-50% EtOAc in hexane). The pure product was afforded as a yellow solid (14 mg, 45% yield).

^{1}H NMR (500 MHz, Chloroform-*d*) δ 8.31 (d, *J* = 8.7 Hz, 2H), 7.67 (d, *J* = 8.7 Hz, 2H), 7.21 (t, *J* = 8.1 Hz, 1H), 6.70 (d, *J* = 8.1 Hz, 1H), 6.65 (d, *J* = 8.1 Hz, 1H), 4.81 (s, 1H), 2.92–2.79 (m, 2H), 2.42 (td, *J* = 11.4, 2.7 Hz, 1H), 2.17 (dd, *J* = 11.3, 9.8 Hz, 1H), 1.46–1.33 (m, 2H), 1.29–1.13 (m, 2H), 0.88–0.79 (m, 1H), 0.70 (d, *J* = 6.7 Hz, 3H).

^{13}C NMR (101 MHz, Chloroform-*d*) δ 153.0, 153.0, 146.9, 143.2, 132.1, 130.2, 123.8, 120.5, 112.1, 110.5, 60.1, 52.8, 32.6, 31.2, 25.4, 19.3.

HRMS (ESI) m/z: $[M-H]^{+}$calcd for $C_{18}H_{19}N_2O_3$: 311.1401. Found: 311.1397

MP 103-105 °C

6-(azepan-1-yl)-4'-nitro-[1,1'-biphenyl]-2-ol (11k)

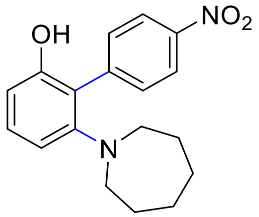

Synthesized according to the general procedure B (column condition: 0-50% EtOAc in hexane). The pure product was afforded as a deep yellow solid (17 mg, 55% yield).

^{1}H NMR (500 MHz, Chloroform-*d*) δ 8.32 (d, *J* = 8.7 Hz, 2H), 7.60 (d, *J* = 8.7 Hz, 2H), 7.18 (t, *J* = 8.1 Hz, 1H), 6.76 (d, *J* = 8.1 Hz, 1H), 6.59 (d, *J* = 8.1 Hz, 1H), 4.68 (s, 1H), 3.02–2.81 (m, 4H), 1.43 (m, 4H), 1.40–1.29 (m, 4H).

^{13}C NMR (101 MHz, Chloroform-*d*) δ 154.9, 153.5, 147.2, 144.2, 132.7, 130.3, 124.3, 120.6, 113.6, 109.7, 55.9, 29.2, 27.4.

HRMS (ESI) m/z: $[M-H]^+$calcd for $C_{18}H_{19}N_2O_3$: 311.1401. Found: 311.1412

MP 146-148 ºC

4'-nitro-6-(octahydroisoquinolin-2(1H)-yl)-[1,1'-biphenyl]-2-ol (11l)

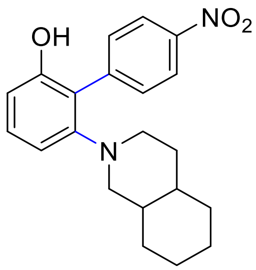

Synthesized according to the general procedure B (column condition: 0-50% EtOAc in hexane). The pure product was afforded as a yellow solid (11 mg, 32% yield).

^{1}H NMR (500 MHz, Chloroform-*d*) δ 8.31 (d, *J* = 8.8 Hz, 2H), 7.67 (d, *J* = 8.8 Hz, 2H), 7.21 (t, *J* = 8.1 Hz, 1H), 6.70 (d, *J* = 7.7 Hz, 1H), 6.65 (d, *J* = 8.1 Hz, 1H), 4.84 (s, 1H), 2.90 (dt, *J* = 11.6, 1.8 Hz, 1H), 2.83 (dd, *J* = 11.2, 1.3 Hz, 1H), 2.47 (td, *J* = 11.7, 2.5 Hz, 1H), 2.23 (t, *J* = 10.7 Hz, 1H), 1.72 – 1.63 (m, 2H), 1.54 (dt, *J* = 12.2, 2.3 Hz, 1H), 1.34 – 1.29 (m, 2H), 1.21 – 1.13 (m, 2H), 0.94 – 0.79 (m, 5H).

^{13}C NMR (101 MHz, Chloroform-*d*) δ 153.1, 146.9, 143.2, 132.1, 123.8, 123.8, 120.3, 112.1, 110.6, 58.7, 53.6, 41.7, 32.9, 30.4, 26.5, 26.1, 22.1, 14.3. [C-OH signal not resolved].

HRMS (ESI) m/z: $[M+H]^+$ calcd for $C_{21}H_{25}N_2O_3$: 353.1860 Found: 353.1867

MP 50-52 ºC

6-(diethylamino)-2'-nitro-[1,1'-biphenyl]-2-ol (11m)

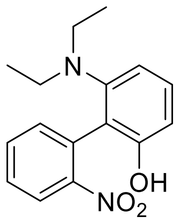

Synthesized according to the general procedure B (column condition: 0-50% EtOAc in hexane). The pure product was afforded as a yellow solid (17 mg, 61% yield).

^{1}H NMR (500 MHz, Chloroform-*d*) δ 8.02 (d, *J* = 8.3 Hz, 1H), 7.65 (t, *J* = 7.6 Hz, 1H), 7.48 (ddd, *J* = 7.2, 4.2, 2.7 Hz, 2H), 7.17 (t, *J* = 8.1 Hz, 1H), 6.71 (d, *J* = 8.2 Hz, 1H), 6.61 (d, *J* = 8.1 Hz, 1H), 4.80 (s, 1H), 2.80 (dq, *J* = 14.0, 7.0 Hz, 2H), 2.70 (dq, *J* = 14.0, 7.1 Hz, 2H), 0.79 (t, *J* = 7.0 Hz, 6H).

^{13}C NMR (126 MHz, Chloroform-*d*) δ 153.0, 150.3, 150.0, 133.8, 132.9, 131.1, 129.2, 128.3, 124.6, 120.7, 114.3, 110.4, 46.3, 11.8.

HRMS (ESI) m/z: $[M+H]^{+}$calcd for $C_{16}H_{19}N_{2}O_{3}$: 287.1390. Found: 287.1384

MP 102-104 ^{o}C

4'-chloro-6-(diethylamino)-2'-nitro-[1,1'-biphenyl]-2-ol (11n)

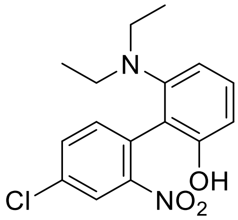

Synthesized according to the general procedure B (column condition: 0-50% EtOAc in hexane). The pure product was afforded as a yellow liquid (17 mg, 54% yield).

^{1}H NMR (500 MHz, Chloroform-*d*) δ 7.97 (d, *J* = 8.4 Hz, 1H), 7.55 (s, 1H), 7.37 (t, *J* = 8.0 Hz, 1H), 7.33 (d, *J* = 8.3 Hz, 1H), 7.22 (d, *J* = 8.1 Hz, 1H), 6.97 (d, *J* = 8.0 Hz, 1H), 3.32 (q, *J* = 7.0 Hz, 4H), 1.08 (t, *J* = 7.1 Hz, 6H).

^{13}C NMR (126 MHz, Chloroform-*d*) δ 158.2, 156.3, 147.6, 132.0, 127.8, 123.9, 123.5, 123.1, 118.6, 115.5, 112.0, 106.2, 46.0, 12.1.

HRMS (APCI) m/z: $[M+H]^{+}$calcd for $C_{16}H_{18}ClN_{2}O_{3}$: 321.1000 Found: 321.0989

5'-chloro-6-(diethylamino)-2'-nitro-[1,1'-biphenyl]-2-ol (11o)

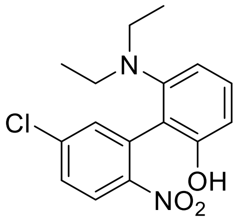

Synthesized according to the general procedure B (column condition: 0-50% EtOAc in hexane). The pure product was afforded as a yellow oil (15 mg, 46% yield).

^{1}H NMR (500 MHz, Chloroform-*d*) δ 7.97 (d, *J* = 9.0 Hz, 1H), 7.49 (s, 1H), 7.42 (d, *J* = 8.8 Hz, 1H), 7.17 (t, *J* = 8.1 Hz, 1H), 6.72 (d, *J* = 8.2 Hz, 1H), 6.56 (d, *J* = 8.0 Hz, 1H), 4.87 (s, 1H), 2.85 (dq, *J* = 14.0, 7.0 Hz, 2H), 2.75 (dq, *J* = 13.9, 7.0 Hz, 2H), 0.84 (t, *J* = 7.1 Hz, 6H).

^{13}C NMR (126 MHz, Chloroform-*d*) δ 152.9, 150.3, 138.7, 133.9, 133.4, 129.5, 128.0, 125.8, 119.6, 114.5, 110.2, 46.2, 11.9.

HRMS (ESI) m/z: $[M+H]^+$ calcd for $C_{16}H_{18}ClN_2O_3$: 321.1000 Found: 321.0995

6-(diethylamino)-4'-methoxy-2'-nitro-[1,1'-biphenyl]-2-ol (11p)

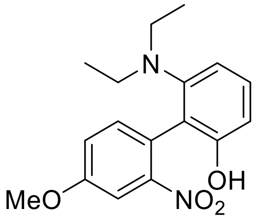

Synthesized according to the general procedure B (column condition: 0-50% EtOAc in hexane). The pure product was afforded as a yellow oil (17 mg, 55% yield).

^{1}H NMR (500 MHz, Chloroform-*d*) δ 7.55 (s, 1H), 7.40 – 7.32 (m, 1H), 7.20 (d, *J* = 8.8 Hz, 1H), 7.16 (t, *J* = 8.3 Hz, 1H), 6.69 (d, *J* = 8.1 Hz, 1H), 6.61 (d, *J* = 8.1 Hz, 1H), 4.76 (s, 1H), 3.91 (s, 3H), 2.80 (dq, *J* = 14.0, 7.0 Hz, 2H), 2.70 (dq, *J* = 13.9, 7.0 Hz, 2H), 0.80 (t, *J* = 7.1 Hz, 6H).

^{13}C NMR (126 MHz, Chloroform-*d*) δ 159.3, 153.3, 150.6, 150.4, 134.6, 128.9, 122.7, 120.5, 119.6, 114.3, 110.3, 109.5, 56.0, 46.2, 11.9.

HRMS (ESI) m/z: $[M-H]^+$ calcd for $C_{17}H_{19}N_2O_4$: 315.1350. Found: 315.1345.

6-(diethylamino)-2'-methoxy-4'-nitro-[1,1'-biphenyl]-2-ol (11q)

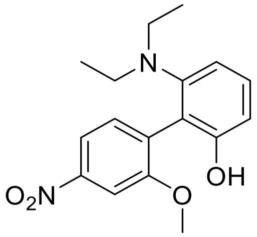

Synthesized according to the general procedure B (column condition: 0-50% EtOAc in hexane). The pure product was afforded as a green solid (27 mg, 85% yield).

^{1}H NMR (400 MHz, Chloroform-*d*) δ 7.93 (dd, *J* = 8.3, 2.2 Hz, 1H), 7.89 (d, *J* = 2.2 Hz, 1H), 7.46 (d, *J* = 8.3 Hz, 1H), 7.23 (t, *J* = 8.1 Hz, 1H), 6.75 (d, *J* = 8.1 Hz, 1H), 6.68 (d, *J* = 8.0 Hz, 1H), 4.91 (s, 1H), 3.93 (s, 3H), 2.76 (m, 4H), 0.81 (t, *J* = 7.1 Hz, 6H).

^{13}C NMR (126 MHz, Chloroform-*d*) δ 157.5, 153.9, 151.4, 148.5, 134.1, 132.6, 129.6, 119.8, 116.4, 114.3, 110.8, 106.3, 56.4, 46.9, 12.4.

HRMS (ESI) m/z: $[M+H]^+$ calcd for $C_{17}H_{21}N_2O_4$: 317.1490 Found: 317.1496

MP 138-140 ºC

6-(diisopropylamino)-2'-methoxy-4'-nitro-[1,1'-biphenyl]-2-ol (11r)

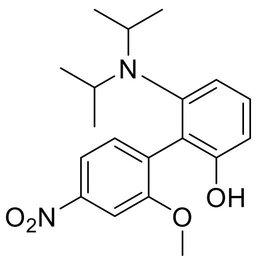

Synthesized according to the general procedure B (column condition: 0-50% EtOAc in hexane). The pure product was afforded as a yellow gummy liquid (17 mg, 50% yield).

^{1}H NMR (400 MHz, Chloroform-*d*) δ 7.93 (dd, *J* = 8.4, 2.2 Hz, 1H), 7.86 (d, *J* = 2.2 Hz, 1H), 7.47 (d, *J* = 8.3 Hz, 1H), 7.22 (t, *J* = 8.1 Hz, 1H), 6.94 (d, *J* = 1.1 Hz, 1H), 6.78 (d, *J* = 8.1 Hz, 1H), 4.83 (s, 1H), 3.90 (s, 3H), 3.27 (hept, *J* = 6.7 Hz, 2H), 0.89 (dd, *J* = 14.9, 6.6 Hz, 12H).

^{13}C NMR (126 MHz, Chloroform-*d*) δ 157.5, 153.9, 150.0, 148.5, 134.6, 132.9,128.8, 123.7, 120.7, 115.9, 112.4, 106.0, 56.3, 50.7, 22.3, 21.5.

HRMS (ESI) m/z: $[M-H]^+$ calcd for $C_{19}H_{23}N_2O_4$: 343.1663 Found: 343.1665

4'-bromo-2',6'-dichloro-6-(diethylamino)-[1,1'-biphenyl]-2-ol (11s)

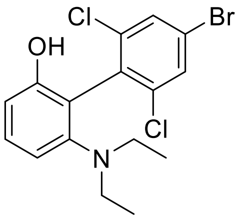

Synthesized according to the general procedure B (column condition: 0-50% EtOAc in hexane). The pure product was afforded as a yellow gummy liquid (18 mg, 46% yield).

^{1}H NMR (400 MHz, Chloroform-*d*) δ 7.61 (s, 2H), 7.26 – 7.22 (m, 1H), 6.72 (d, *J* = 8.2 Hz, 1H), 6.62 (d, *J* = 8.1 Hz, 1H), 4.56 (s, 1H), 2.85 (q, *J* = 7.0 Hz, 4H), 0.85 (t, *J* = 7.1 Hz, 6H).

^{13}C NMR (126 MHz, Chloroform-*d*) δ 153.4, 151.9, 137.7, 133.9, 131.2, 129.9, 121.7, 118.9, 114.1, 109.8, 46.6, 12.5.

HRMS (APCI) m/z: $[M]^+$calcd for $C_{16}H_{16}BrCl_2NO$: 386.9792 Found: 386.9791

4'-amino-6-(diethylamino)-[1,1'-biphenyl]-2-ol (17)

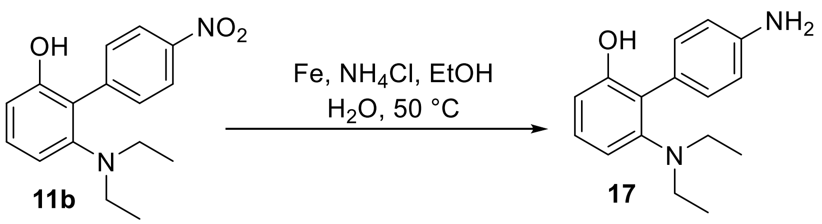

A 2-5 mL oven-dried microwave vial was charged with the corresponding nitrobiaryl **11b** (1.0 equiv), ammonium chloride (0.7 equiv), Fe filings (5.0 equiv) and water (0.33 M). EtOH (0.5 M) was added via syringe and the resulting suspension was stirred at 50 °C for 16 h. The reaction mixture was cooled to room temperature and filtered. Water was added and the aqueous layer was extracted with EtOAc × 3. The combined organic layers were washed with brine, dried over MgSO4, filtered, and concentrated in vacuo. The crude product was then purified by flash column chromatography (column condition: 0-50% EtOAc in hexane). The pure product was afforded as a yellow solid (19 mg, 74% yield).

^{1}H NMR (500 MHz, Chloroform-*d*) δ 7.14 (d, *J* = 8.2 Hz, 3H), 6.79 (d, *J* = 8.4 Hz, 2H), 6.66 (d, *J* = 8.1 Hz, 2H), 5.08 (s, 1H), 3.75 (s, 2H), 2.81 (q, *J* = 7.1 Hz, 4H), 0.84 (t, *J* = 7.1 Hz, 6H).

^{13}C NMR (126 MHz, Chloroform-*d*) δ 154.0, 150.6, 145.9, 131.9, 128.1, 124.5, 123.9, 115.9, 113.5, 109.2, 46.6, 12.5.

HRMS (ESI) m/z: $[M-H]^+$calcd for $C_{16}H_{19}N_2O$: 255.1503 Found:255.1496

MP 178-180 ^oC

7-chloro-N,N-diethyldibenzo[b,d]furan-1-amine (18)

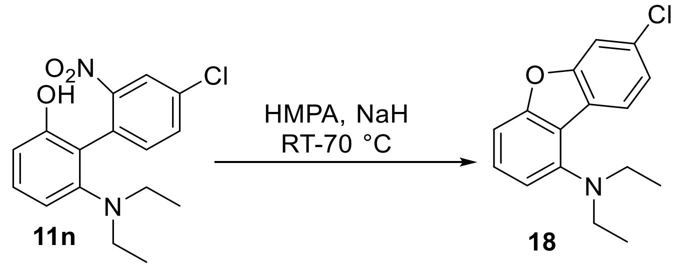

A solution of the phenol **11n** (0.1 mmol) in hexamethylphosphoric triamide (1 ml) was added to a stirred suspension of sodium hydride (0.2 mmol) in hexamethylphosphoric triamide (1 ml), and the mixture was stirred at room temperature for 24 h. The mixture was heated at 70 ^oC for 16 h, cooled, and poured into 5% aq. HCI. The aqueous mixture was extracted with ether, and the ethereal phase separated and washed well with water. The organic phase was dried (Na_2SO_4), and the solvent evaporated. The crude product was

purified by column chromatography (column condition: 0-50% EtOAc in hexane).[4] The pure product was afforded as a yellow oil (19 mg, 68% yield).

^{1}H NMR (500 MHz, Chloroform-*d*) δ 7.97 (d, *J* = 8.3 Hz, 1H), 7.55 (d, *J* = 1.9 Hz, 1H), 7.37 (t, *J* = 8.0 Hz, 1H), 7.33 (dd, *J* = 8.3, 1.9 Hz, 1H), 7.22 (d, *J* = 8.2 Hz, 1H), 6.97 (d, *J* = 7.9 Hz, 1H), 3.32 (q, *J* = 7.0 Hz, 4H), 1.08 (t, *J* = 7.0 Hz, 6H).

^{13}C NMR (126 MHz, Chloroform-*d*) δ 157.9, 156.0, 147.4, 131.8, 127.6, 123.6, 123.2, 122.9, 118.4, 115.3, 111.8, 105.9, 45.7, 11.8.

HRMS (ESI) m/z: $[M+H]^+$ calcd for $C_{16}H_{17}ClNO$: 274.0993 Found:274.0990

5. Single crystal XRD Data for 11b

Data collection, crystal structure determination, and refinements were done by the X-ray crystallography service (Dr Avantika Hasija) at The University of Manchester.

SCXRD experimental details

The crystals were prepared by layering of hexane onto a solution of the compound in CH_2Cl_2. Single crystal X-ray diffraction data for the crystals were collected on the Rigaku Oxford Diffraction Supernova, four-circle diffractometer equipped with CCD plate detector, using micro-focus sealed X-ray tube of Mo Kα radiation (λ = 0.71073 Å).[1] Data reduction was performed using CrysAlisPro software Suite.[1] The crystal structure was solved by Intrinsic Phasing using the ShelXT program.[2] All structures were refined by the full-matrix least-squares method using ShelXL 2018[3] present in the Olex2 interface.[4] The absorption correction is performed using Analytical method by identifying crystal faces. All non-hydrogen atoms were refined anisotropically, and all hydrogen atoms were positioned geometrically (hydrogen atom was located from the difference Fourier map when attached to nitrogen atom) and refined using a riding model.

Data availability

Crystallographic data for compound **11b** has been deposited in the Cambridge Crystallographic Data Centre, with deposition number **CCDC 2312409**, and is available free of charge via https://www.ccdc.cam.ac.uk/structures/.

X-ray crystal structure of 6-(diethylamino)-4'-nitro-[1,1'-biphenyl]-2-ol (11b)

(Ellipsoid Contour Probability at 50%)

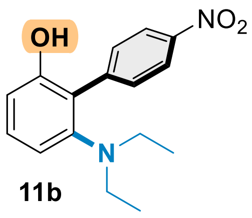

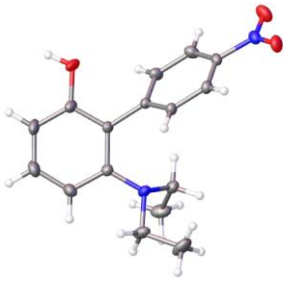

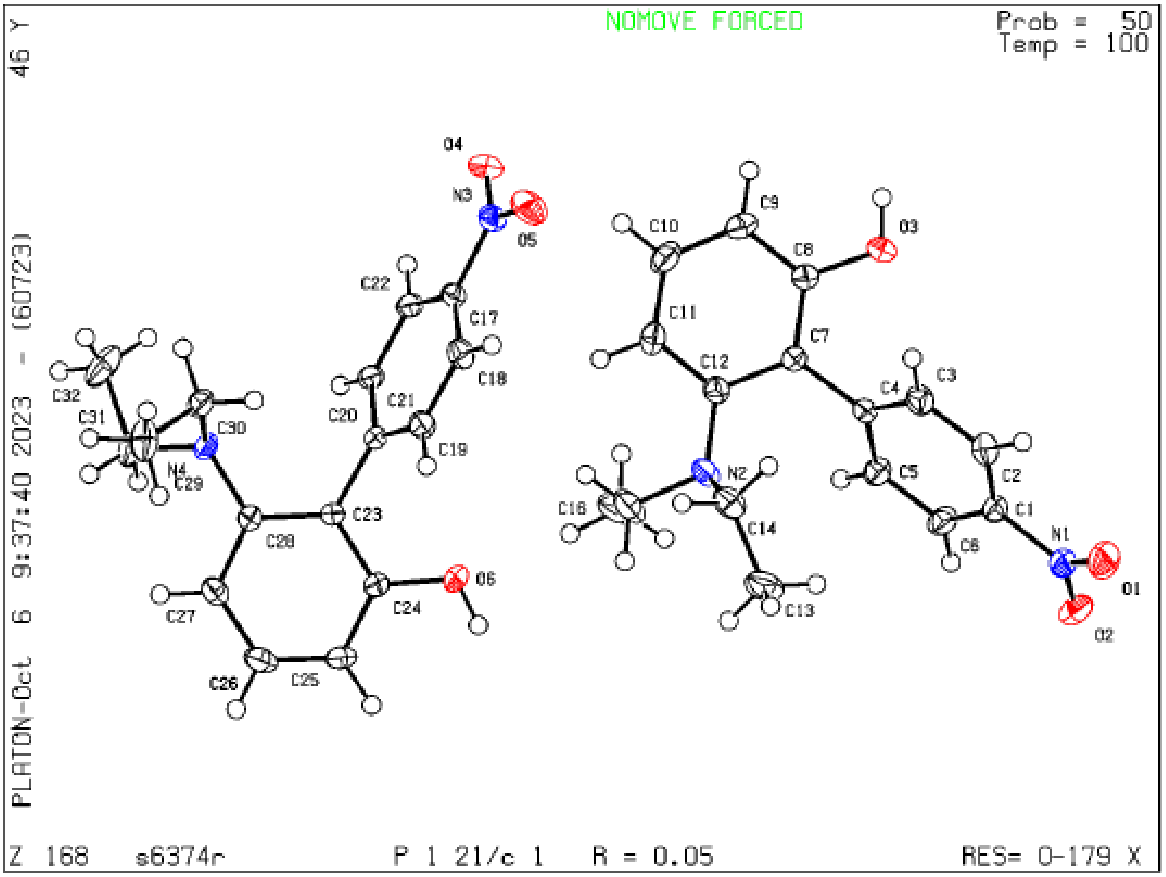

Empirical formula	C16 H18 N2 O3
Formula weight	286.32
Temperature (K)	100
Crystal habit	Block
Crystal system	Monoclinic
Space group	P 1 21/c 1
a (Å)	15.8814(4)
b (Å)	17.4198(4)
c (Å)	11.2680(3)
α (°)	90

β (°)	108.438(3)
γ (°)	90
Volume ($Å^3$)	2957.28(14)
Z	8
P_{calc} (g/cm^3)	1.286
μ (mm^{-1})	0.090
F(000)	1216.0
Radiation (Å)	MoKα (λ = 0.71073)
Independent reflections	9027
Data/parameters	9027/391
Goodness-of-fit on F^2	1.050
Final R indexes [I>=2σ (I)]	wR_2 = 0.1507
Final R indexes [all data]	R_1 = 0.0528

6. NMR spectra for synthesized compounds

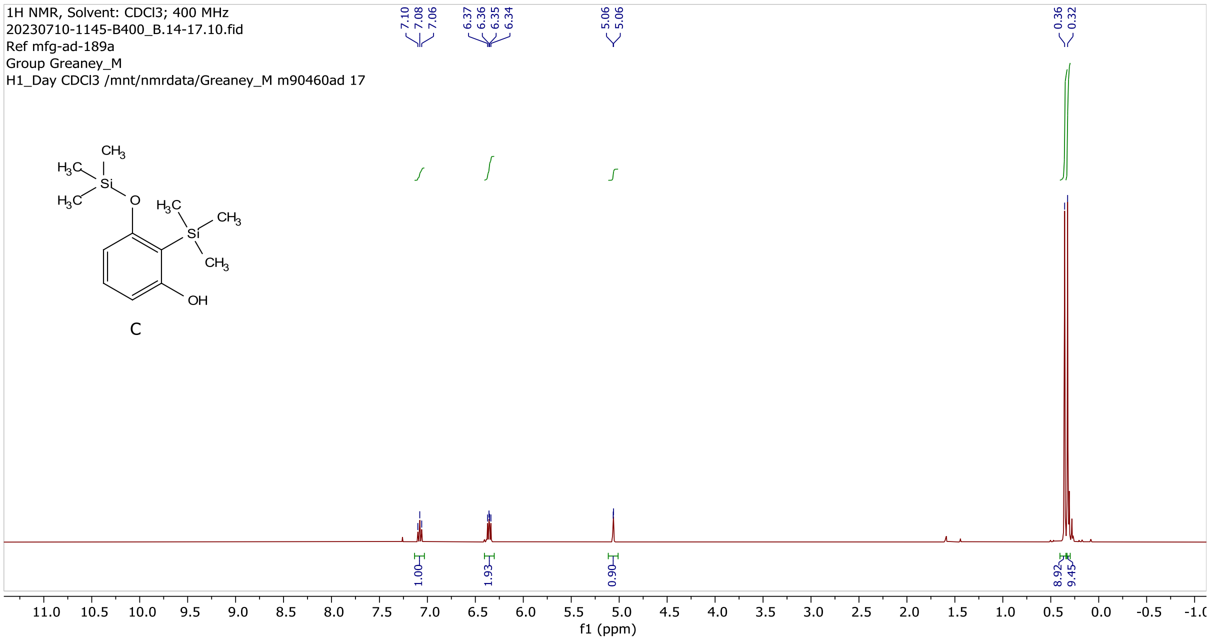

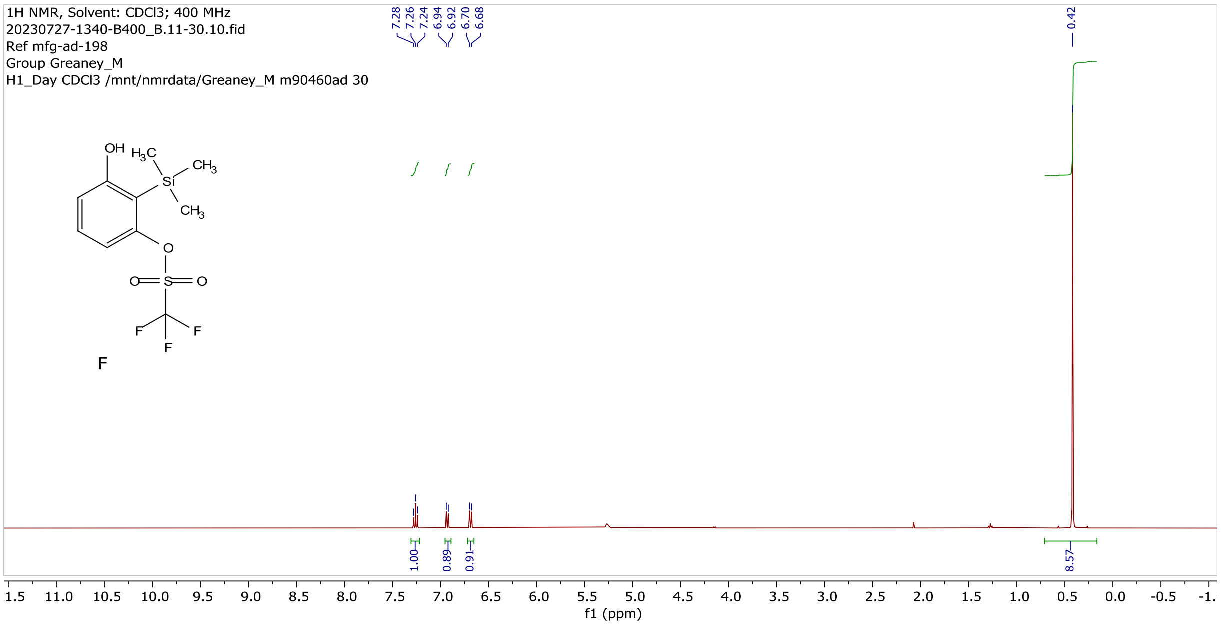

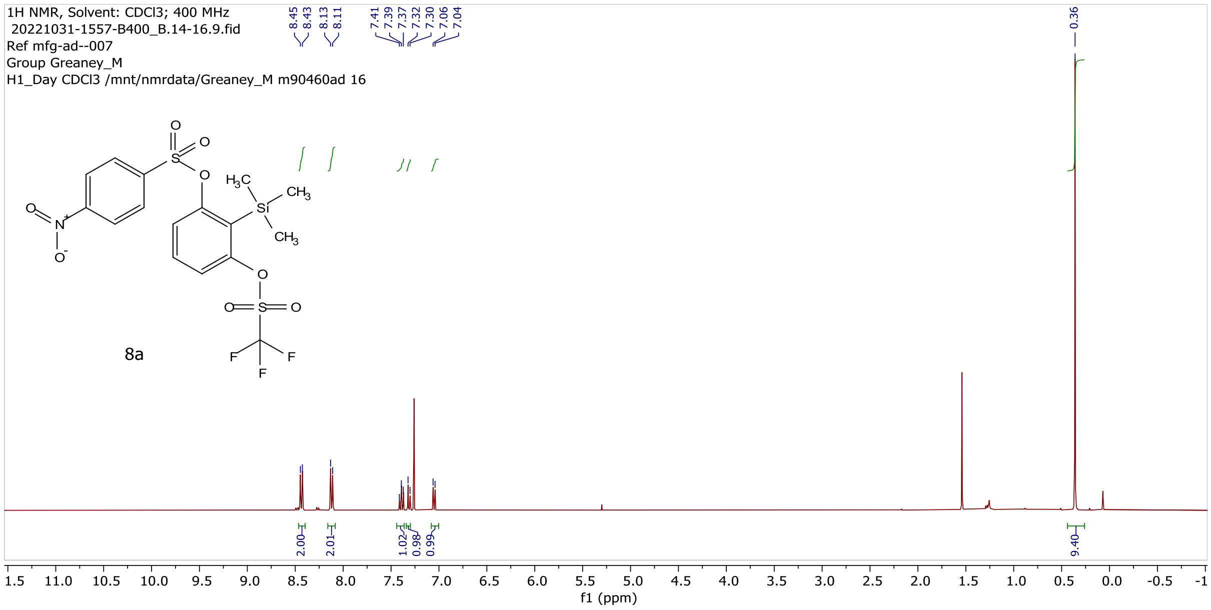
1H NMR, Solvent: CDCl3; 400 MHz
20221031-1557-B400_B.14-16.9.fid
Ref mfg-ad--007
Group Greaney_M
H1_Day CDCl3 /mnt/nmrdata/Greaney_M m90460ad 16
8a
f1 (ppm)

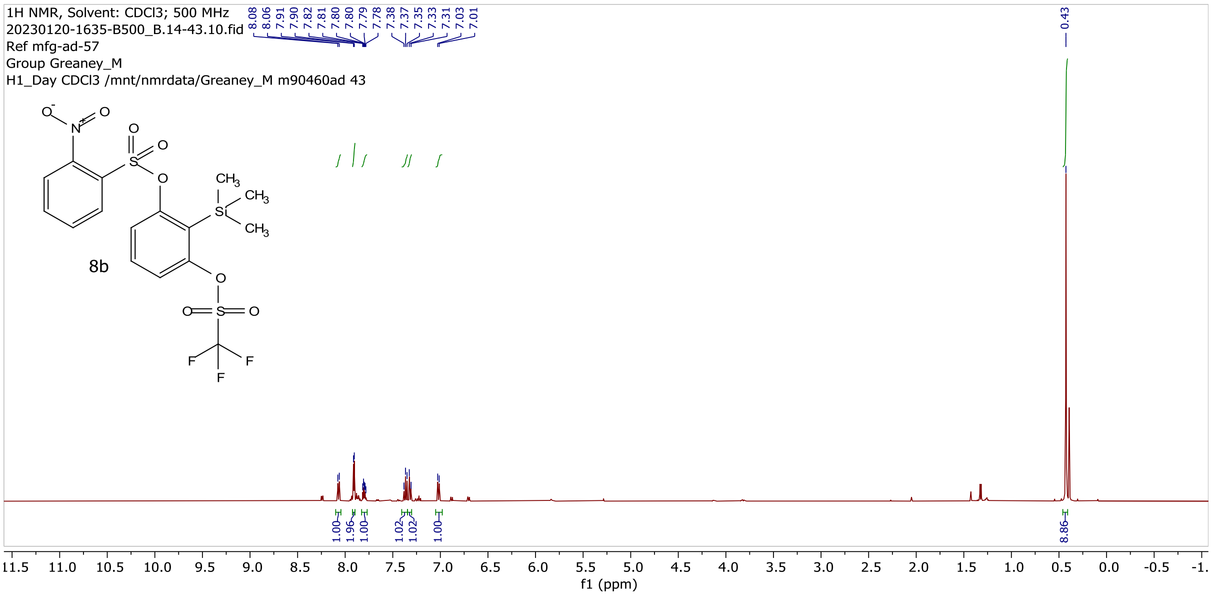
1H NMR, Solvent: CDCl3; 500 MHz
20230120-1635-B500_B.14-43.10.fid
Ref mfg-ad-57
Group Greaney_M
H1_Day CDCl3 /mnt/nmrdata/Greaney_M m90460ad 43
8b
f1 (ppm)

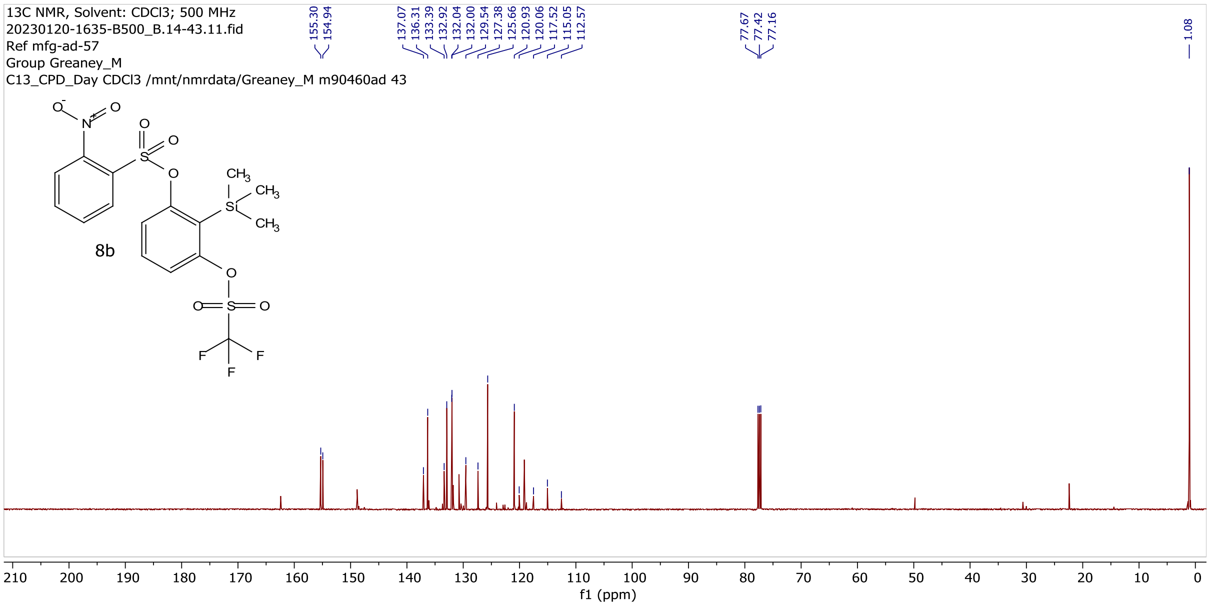
13C NMR, Solvent: CDCl3; 500 MHz
20230120-1635-B500_B.14-43.11.fid
Ref mfg-ad-57
Group Greaney_M
C13_CPD_Day CDCl3 /mnt/nmrdata/Greaney_M m90460ad 43
155.30
154.94
137.07
136.31
133.39
132.92
132.04
132.00
129.54
127.38
125.66
120.93
120.06
117.52
115.05
112.57
77.67
77.42
77.16
1.08
8b
f1 (ppm)

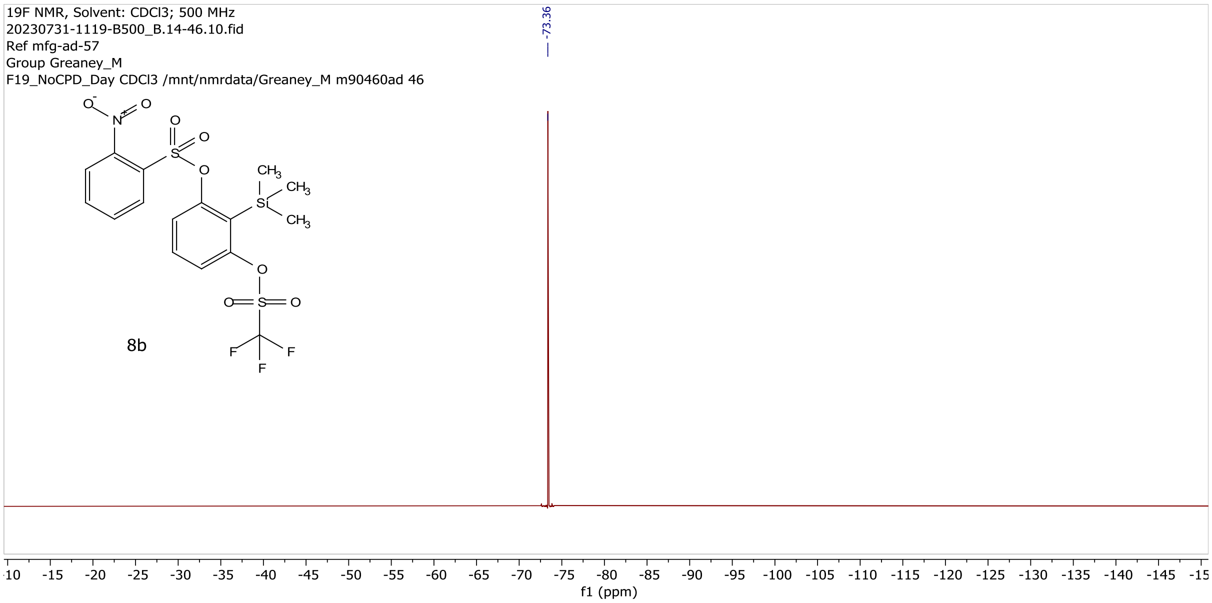
19F NMR, Solvent: CDCl3; 500 MHz
20230731-1119-B500_B.14-46.10.fid
Ref mfg-ad-57
Group Greaney_M
F19_NoCPD_Day CDCl3 /mnt/nmrdata/Greaney_M m90460ad 46
-73.36
8b
f1 (ppm)

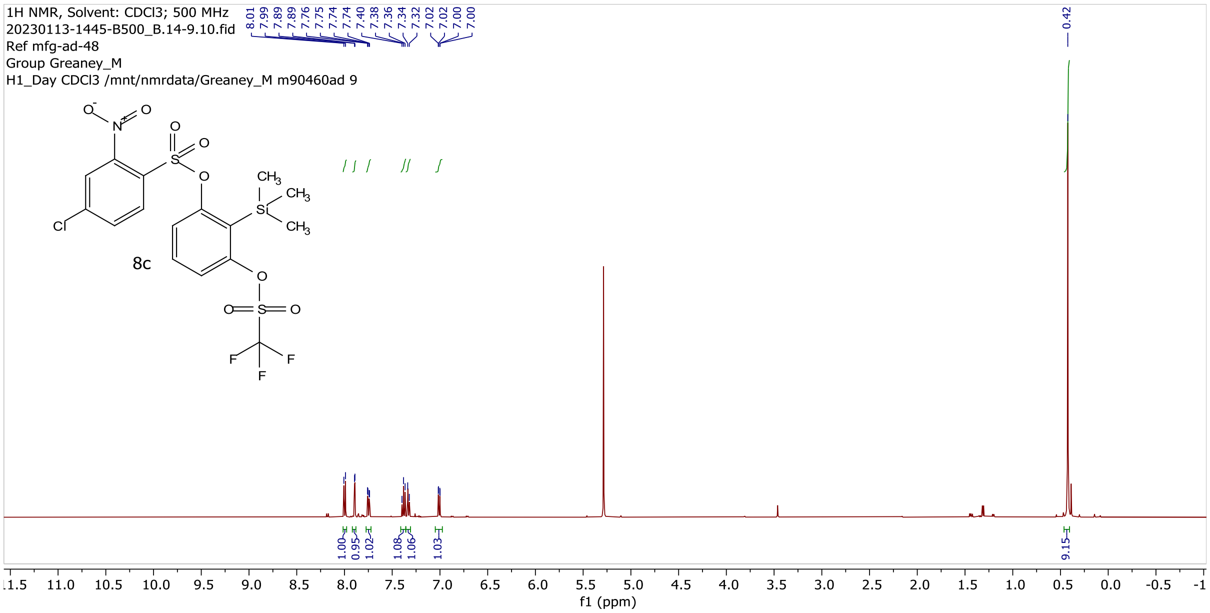
1H NMR, Solvent: CDCl3; 500 MHz
20230113-1445-B500_B.14-9.10.fid
Ref mfg-ad-48
Group Greaney_M
H1_Day CDCl3 /mnt/nmrdata/Greaney_M m90460ad 9
8.01 7.99 7.89 7.89 7.76 7.75 7.74 7.74 7.40 7.38 7.36 7.34 7.32 7.02 7.02 7.00 7.00
0.42
8c
1.00 0.95 1.02 1.08 1.06 1.03 9.15
f1 (ppm)

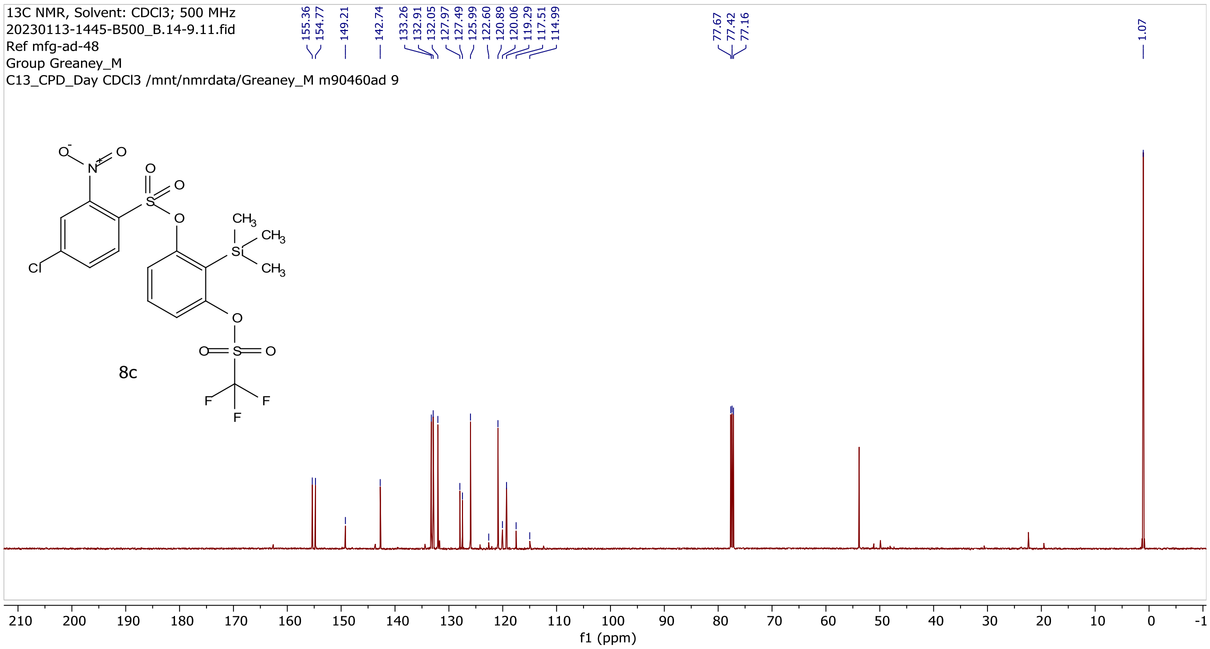
13C NMR, Solvent: CDCl3; 500 MHz
20230113-1445-B500_B.14-9.11.fid
Ref mfg-ad-48
Group Greaney_M
C13_CPD_Day CDCl3 /mnt/nmrdata/Greaney_M m90460ad 9
155.36 154.77 149.21 142.74 133.26 132.91 132.05 127.97 127.49 125.99 122.60 120.89 120.06 119.29 117.51 114.99
77.67 77.42 77.16
1.07
8c
f1 (ppm)

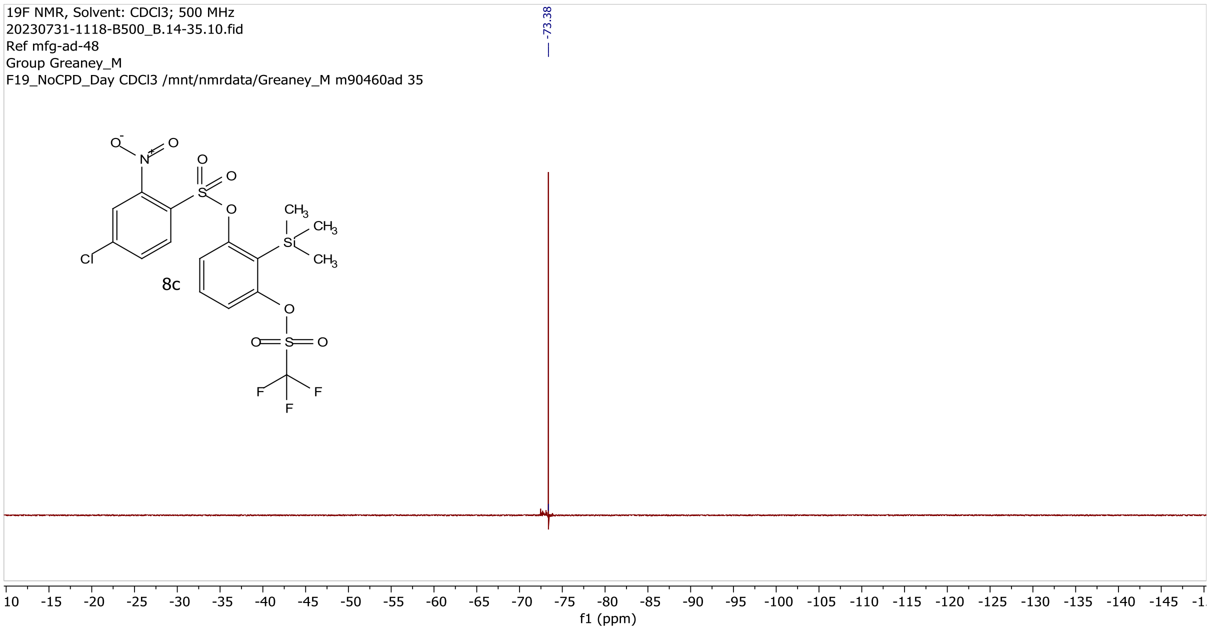
19F NMR, Solvent: CDCl3; 500 MHz
20230731-1118-B500_B.14-35.10.fid
Ref mfg-ad-48
Group Greaney_M
F19_NoCPD_Day CDCl3 /mnt/nmrdata/Greaney_M m90460ad 35
-73.38
8c
f1 (ppm)

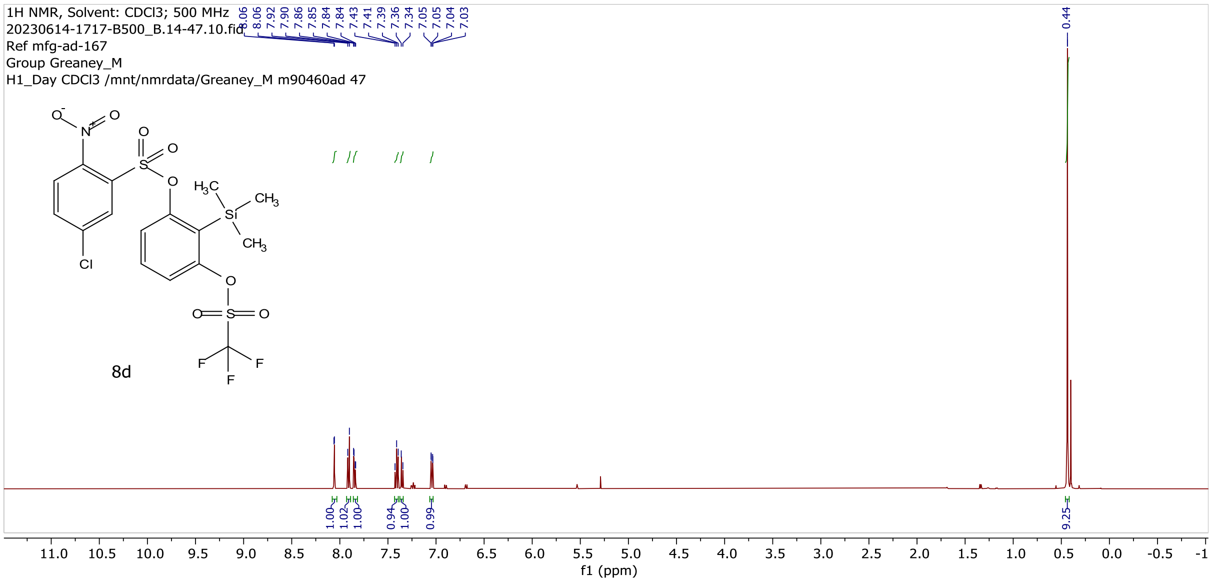
1H NMR, Solvent: CDCl3; 500 MHz
20230614-1717-B500_B.14-47.10.fid
Ref mfg-ad-167
Group Greaney_M
H1_Day CDCl3 /mnt/nmrdata/Greaney_M m90460ad 47
8.06 8.06 7.92 7.90 7.86 7.85 7.84 7.84 7.43 7.41 7.39 7.36 7.34 7.05 7.04 7.03
0.44
8d
1.00 1.02 1.00 0.94 1.00 0.99 9.25
f1 (ppm)

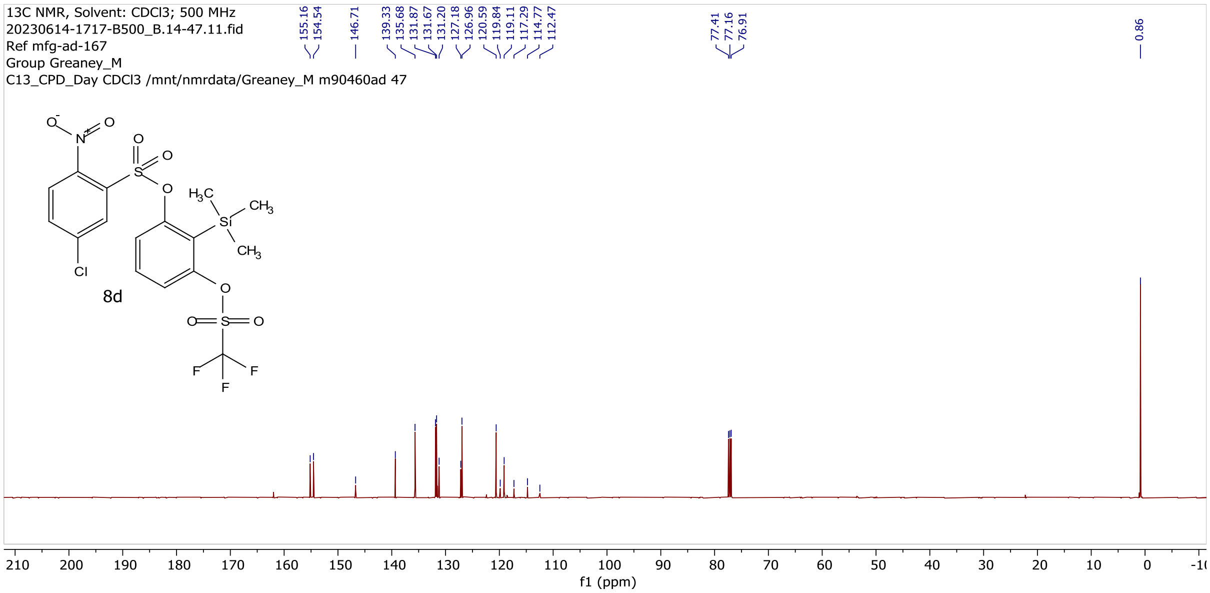
13C NMR, Solvent: CDCl3; 500 MHz
20230614-1717-B500_B.14-47.11.fid
Ref mfg-ad-167
Group Greaney_M
C13_CPD_Day CDCl3 /mnt/nmrdata/Greaney_M m90460ad 47
155.16
154.54
146.71
139.33
135.68
131.87
131.67
131.20
127.18
126.96
120.59
119.84
119.11
117.29
114.77
112.47
77.41
77.16
76.91
0.86
8d
f1 (ppm)

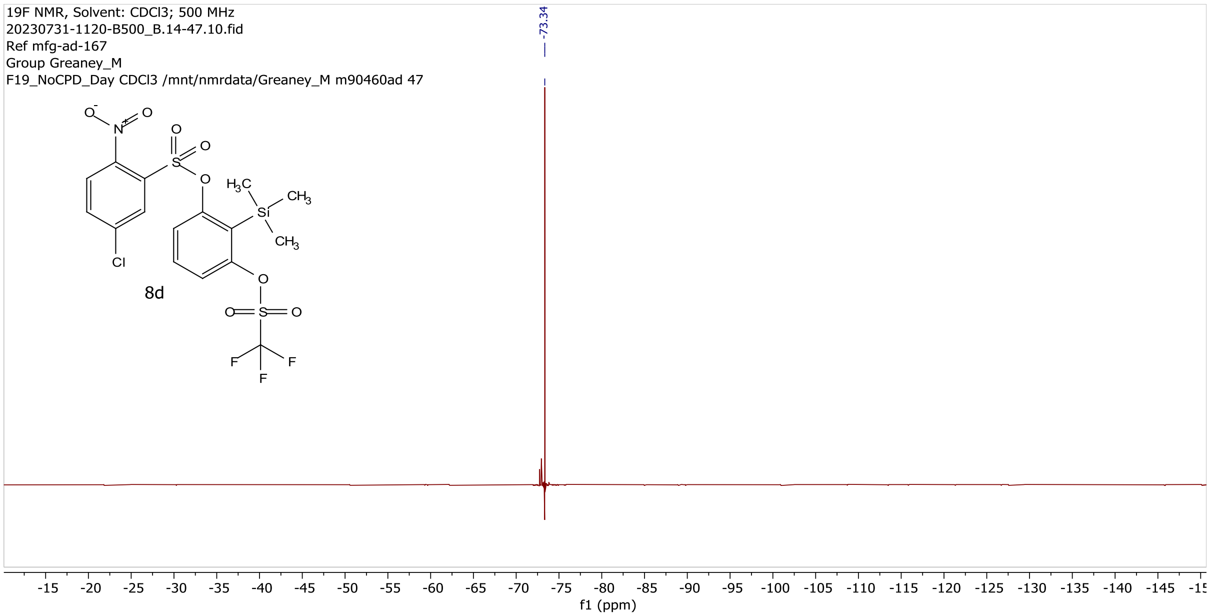
19F NMR, Solvent: CDCl3; 500 MHz
20230731-1120-B500_B.14-47.10.fid
Ref mfg-ad-167
Group Greaney_M
F19_NoCPD_Day CDCl3 /mnt/nmrdata/Greaney_M m90460ad 47
-73.34
8d
f1 (ppm)

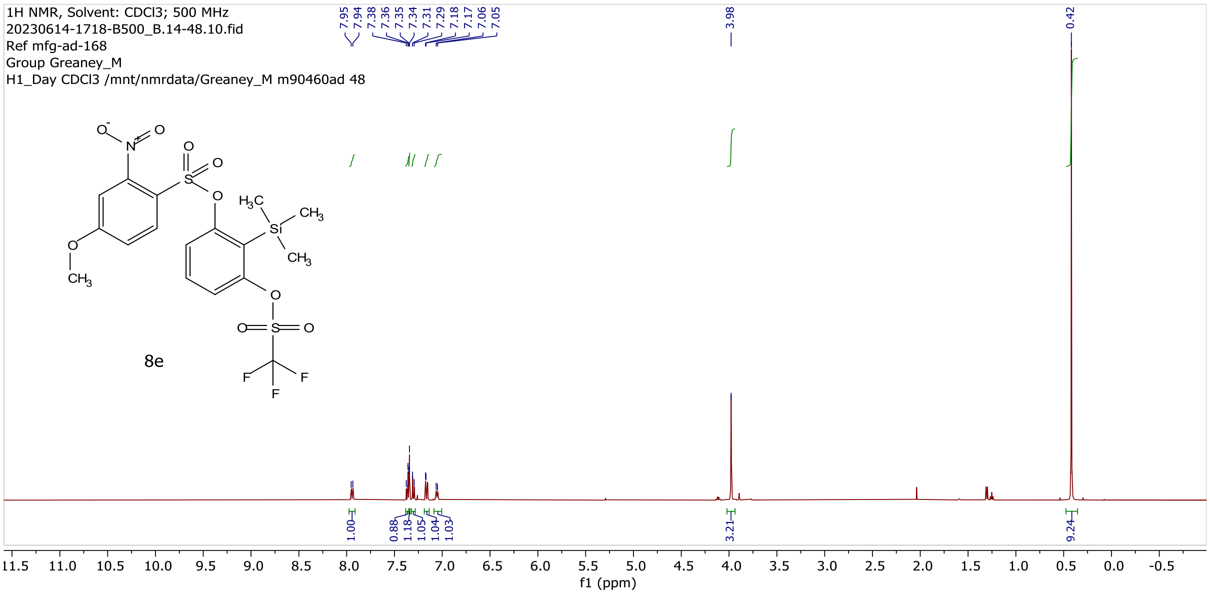

1H NMR, Solvent: CDCl3; 500 MHz
20230614-1718-B500_B.14-48.10.fid
Ref mfg-ad-168
Group Greaney_M
H1_Day CDCl3 /mnt/nmrdata/Greaney_M m90460ad 48
7.95
7.94
7.38
7.36
7.35
7.34
7.31
7.29
7.18
7.17
7.06
7.05
3.98
0.42
8e
1.00
0.88
1.18
1.05
1.04
1.03
3.21
9.24
f1 (ppm)

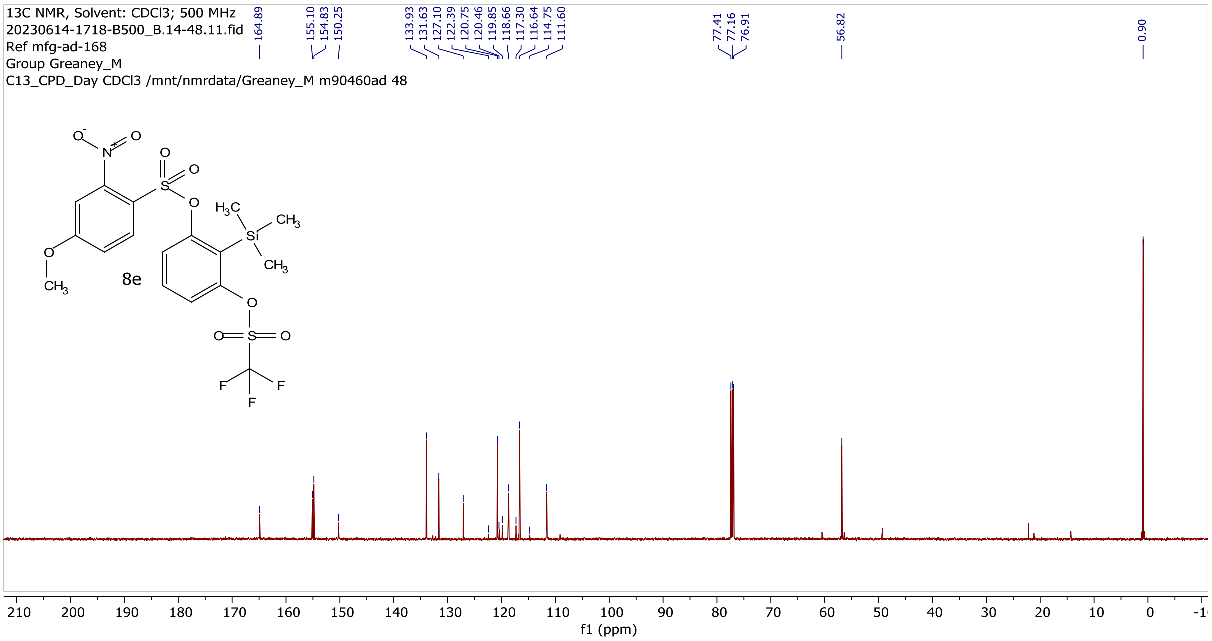

13C NMR, Solvent: CDCl3; 500 MHz
20230614-1718-B500_B.14-48.11.fid
Ref mfg-ad-168
Group Greaney_M
C13_CPD_Day CDCl3 /mnt/nmrdata/Greaney_M m90460ad 48
164.89
155.10
154.83
150.25
133.93
131.63
127.10
122.39
120.75
120.46
119.85
118.66
117.30
116.64
114.75
111.60
77.41
77.16
76.91
56.82
0.90
8e
f1 (ppm)

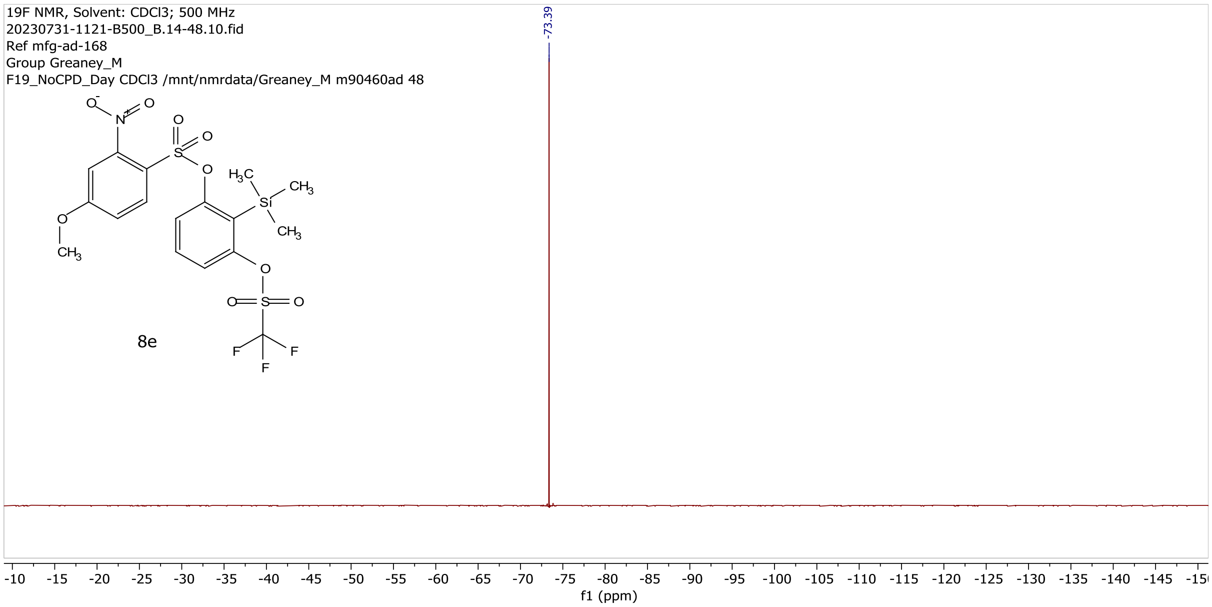
19F NMR, Solvent: CDCl3; 500 MHz
20230731-1121-B500_B.14-48.10.fid
Ref mfg-ad-168
Group Greaney_M
F19_NoCPD_Day CDCl3 /mnt/nmrdata/Greaney_M m90460ad 48
-73.39
8e
f1 (ppm)

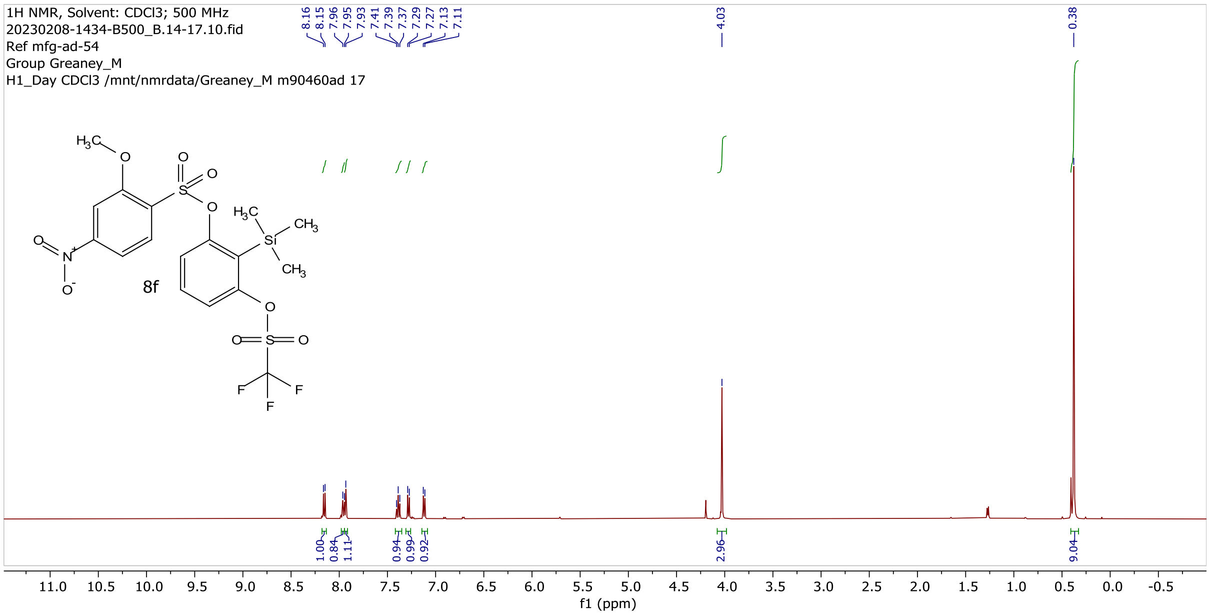
1H NMR, Solvent: CDCl3; 500 MHz
20230208-1434-B500_B.14-17.10.fid
Ref mfg-ad-54
Group Greaney_M
H1_Day CDCl3 /mnt/nmrdata/Greaney_M m90460ad 17
8.16
8.15
7.96
7.95
7.93
7.41
7.39
7.37
7.29
7.27
7.13
7.11
4.03
0.38
8f
1.00
0.84
1.11
0.94
0.99
0.92
2.96
9.04
f1 (ppm)

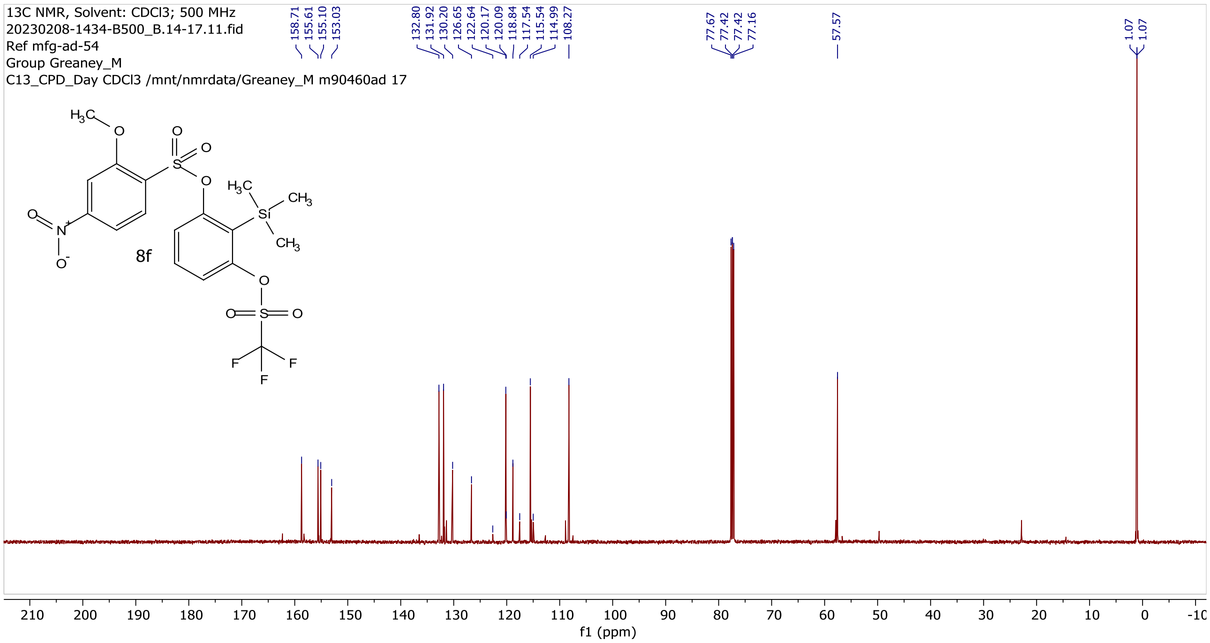
13C NMR, Solvent: CDCl3; 500 MHz
20230208-1434-B500_B.14-17.11.fid
Ref mfg-ad-54
Group Greaney_M
C13_CPD_Day CDCl3 /mnt/nmrdata/Greaney_M m90460ad 17
158.71
155.61
155.10
153.03
132.80
131.92
130.20
126.65
122.64
120.17
120.09
118.84
117.54
115.54
114.99
108.27
77.67
77.42
77.42
77.16
57.57
1.07
1.07
8f
f1 (ppm)

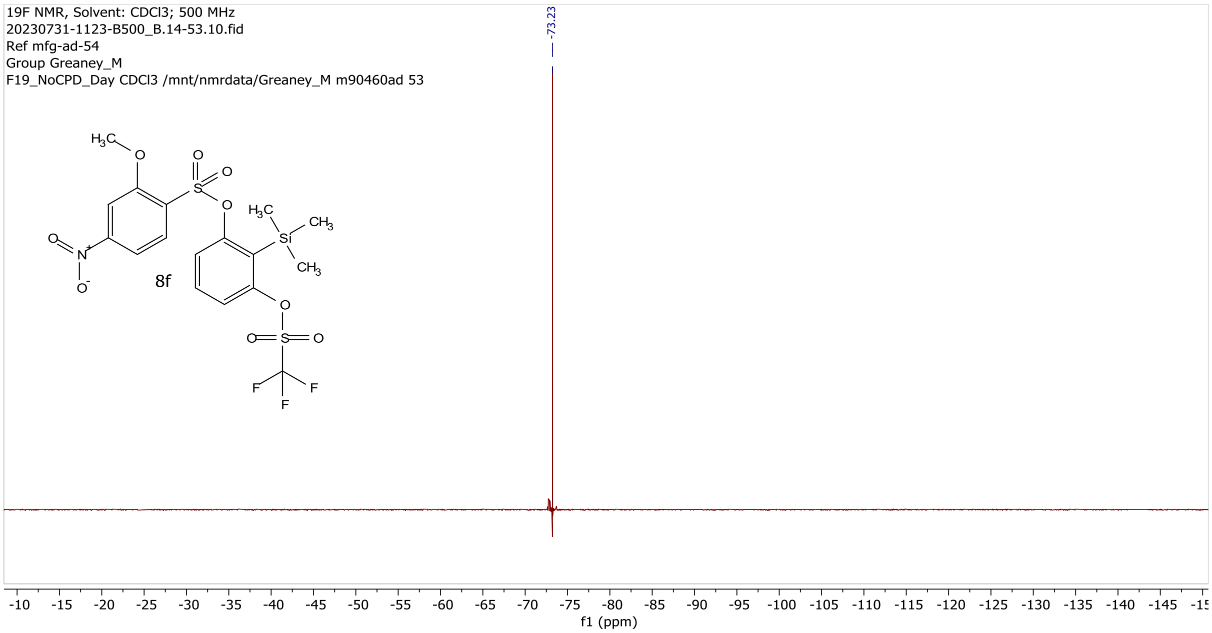
19F NMR, Solvent: CDCl3; 500 MHz
20230731-1123-B500_B.14-53.10.fid
Ref mfg-ad-54
Group Greaney_M
F19_NoCPD_Day CDCl3 /mnt/nmrdata/Greaney_M m90460ad 53
-73.23
8f
f1 (ppm)

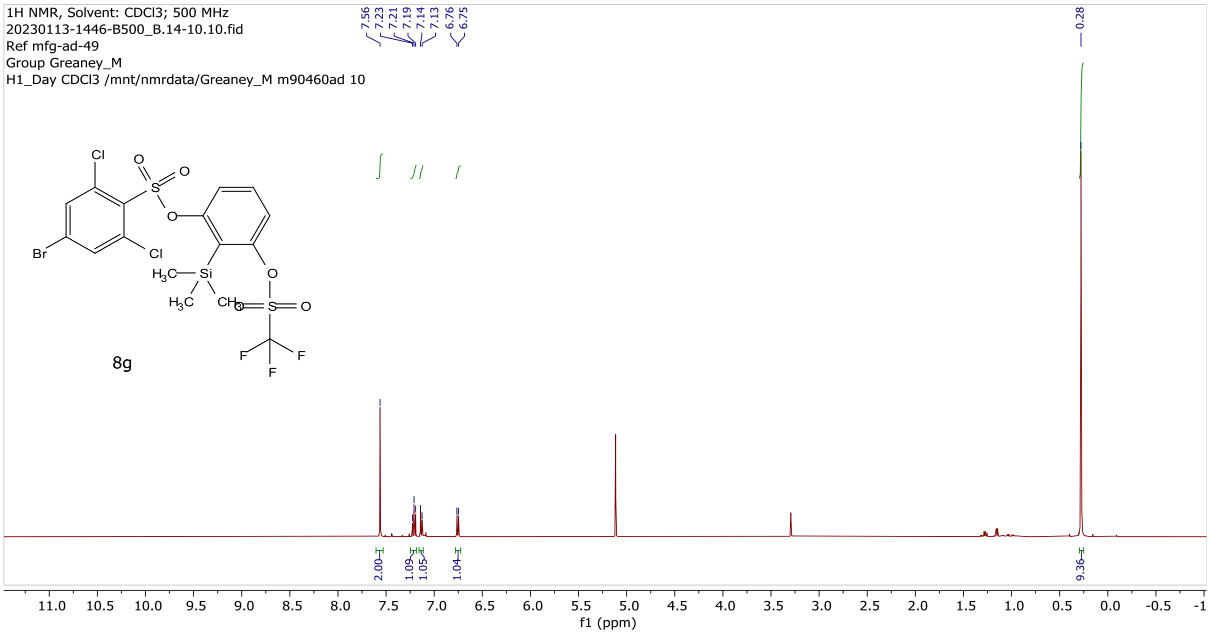

1H NMR, Solvent: CDCl3; 500 MHz
20230113-1446-B500_B.14-10.10.fid
Ref mfg-ad-49
Group Greaney_M
H1_Day CDCl3 /mnt/nmrdata/Greaney_M m90460ad 10
7.56
7.23
7.21
7.19
7.14
7.13
6.76
6.75
0.28
8g
2.00
1.09
1.05
1.04
9.36
f1 (ppm)

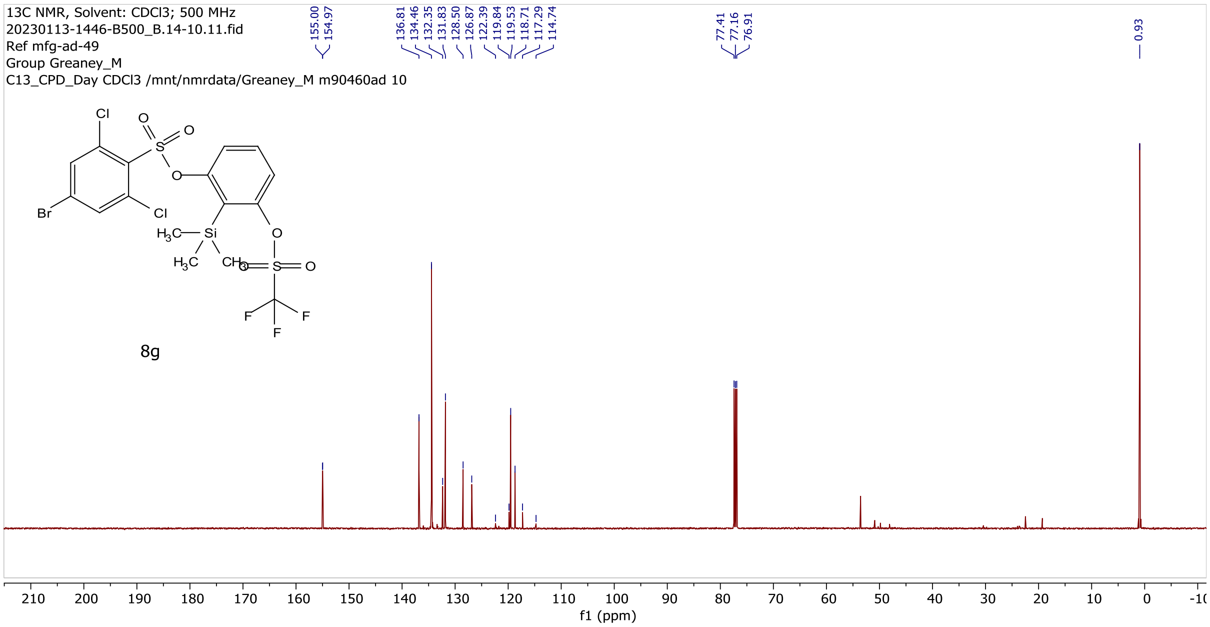

13C NMR, Solvent: CDCl3; 500 MHz
20230113-1446-B500_B.14-10.11.fid
Ref mfg-ad-49
Group Greaney_M
C13_CPD_Day CDCl3 /mnt/nmrdata/Greaney_M m90460ad 10
155.00
154.97
136.81
134.46
132.35
131.83
128.50
126.87
122.39
119.84
119.53
118.71
117.29
114.74
77.41
77.16
76.91
0.93
8g
f1 (ppm)

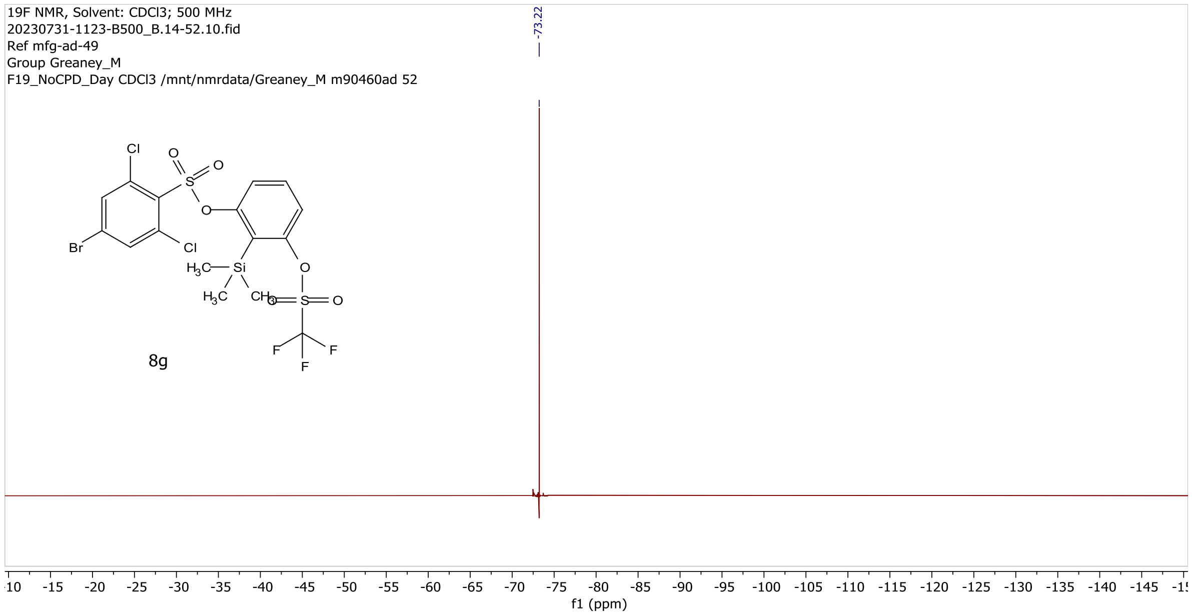
19F NMR, Solvent: CDCl3; 500 MHz
20230731-1123-B500_B.14-52.10.fid
Ref mfg-ad-49
Group Greaney_M
F19_NoCPD_Day CDCl3 /mnt/nmrdata/Greaney_M m90460ad 52
-73.22
8g
f1 (ppm)

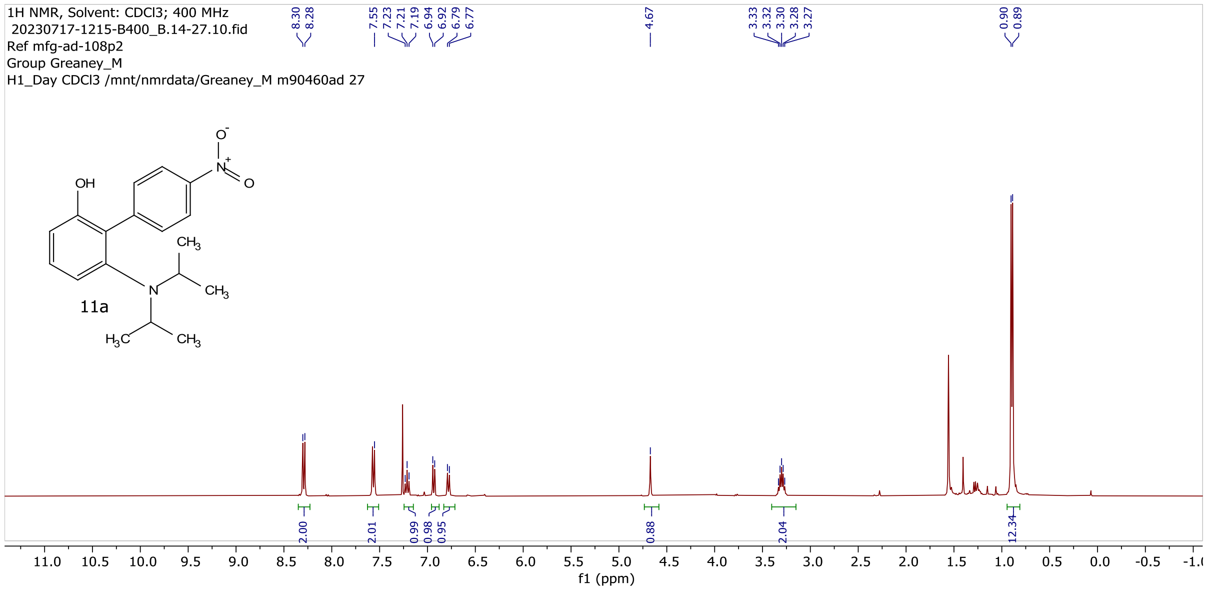
1H NMR, Solvent: CDCl3; 400 MHz
20230717-1215-B400_B.14-27.10.fid
Ref mfg-ad-108p2
Group Greaney_M
H1_Day CDCl3 /mnt/nmrdata/Greaney_M m90460ad 27
8.30
8.28
7.55
7.23
7.21
7.19
6.94
6.92
6.79
6.77
4.67
3.33
3.32
3.30
3.28
3.27
0.90
0.89
11a
2.00
2.01
0.99
0.98
0.95
0.88
2.04
12.34
f1 (ppm)

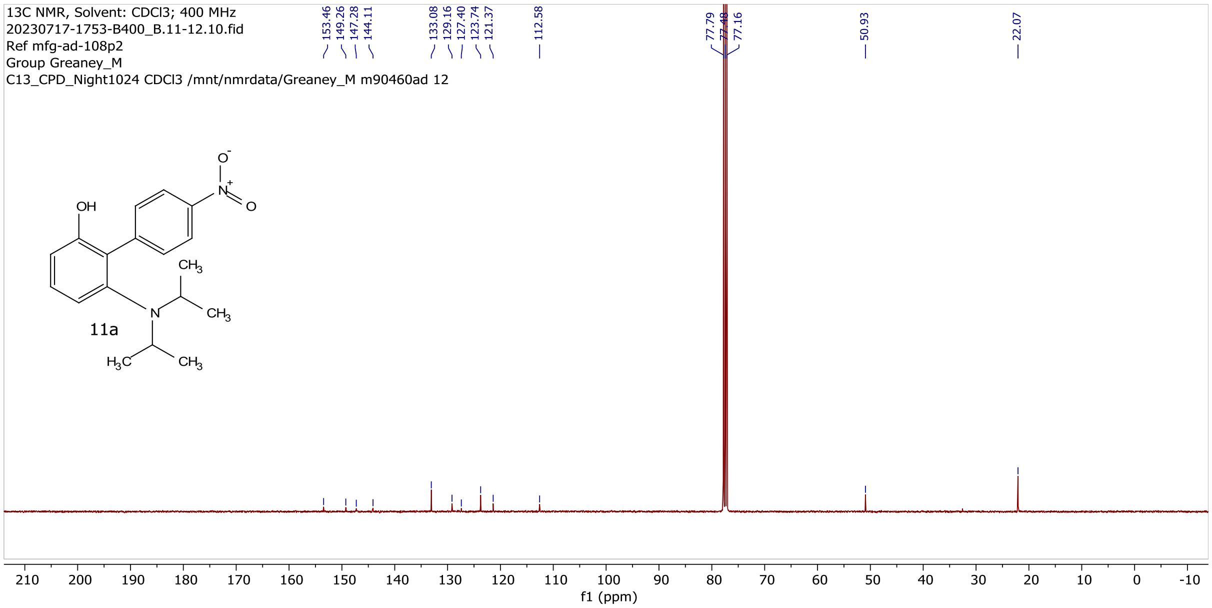
13C NMR, Solvent: CDCl3; 400 MHz
20230717-1753-B400_B.11-12.10.fid
Ref mfg-ad-108p2
Group Greaney_M
C13_CPD_Night1024 CDCl3 /mnt/nmrdata/Greaney_M m90460ad 12
153.46
149.26
147.28
144.11
133.08
129.16
127.40
123.74
121.37
112.58
77.79
77.16
50.93
22.07
OH
CH3
CH3
H3C
CH3
N
11a
210 200 190 180 170 160 150 140 130 120 110 100 90 80 70 60 50 40 30 20 10 0 -10
f1 (ppm)

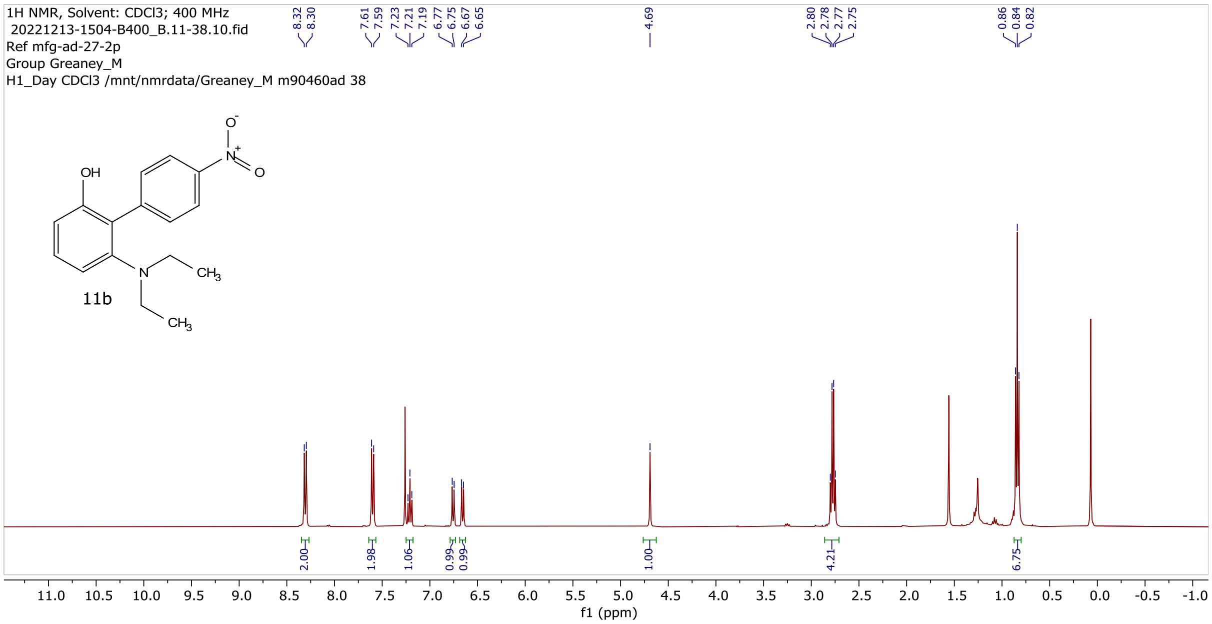
1H NMR, Solvent: CDCl3; 400 MHz
20221213-1504-B400_B.11-38.10.fid
Ref mfg-ad-27-2p
Group Greaney_M
H1_Day CDCl3 /mnt/nmrdata/Greaney_M m90460ad 38
8.32
8.30
7.61
7.59
7.23
7.21
7.19
6.77
6.75
6.67
6.65
4.69
2.80
2.78
2.77
2.75
0.86
0.84
0.82
OH
N
CH3
CH3
11b
2.00
1.98
1.06
0.99
0.99
1.00
4.21
6.75
11.0 10.5 10.0 9.5 9.0 8.5 8.0 7.5 7.0 6.5 6.0 5.5 5.0 4.5 4.0 3.5 3.0 2.5 2.0 1.5 1.0 0.5 0.0 -0.5 -1.0
f1 (ppm)

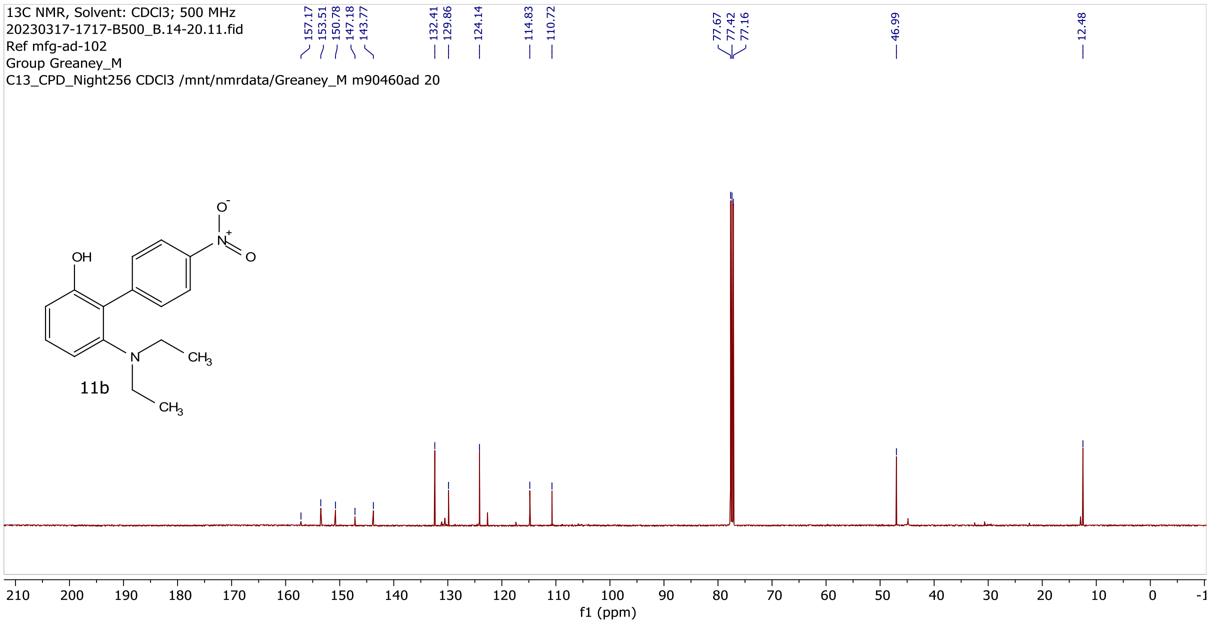
13C NMR, Solvent: CDCl3; 500 MHz
20230317-1717-B500_B.14-20.11.fid
Ref mfg-ad-102
Group Greaney_M
C13_CPD_Night256 CDCl3 /mnt/nmrdata/Greaney_M m90460ad 20
157.17
153.51
150.78
147.18
143.77
132.41
129.86
124.14
114.83
110.72
77.67
77.42
77.16
46.99
12.48
OH
O-
N+
O
N
CH3
CH3
11b
f1 (ppm)

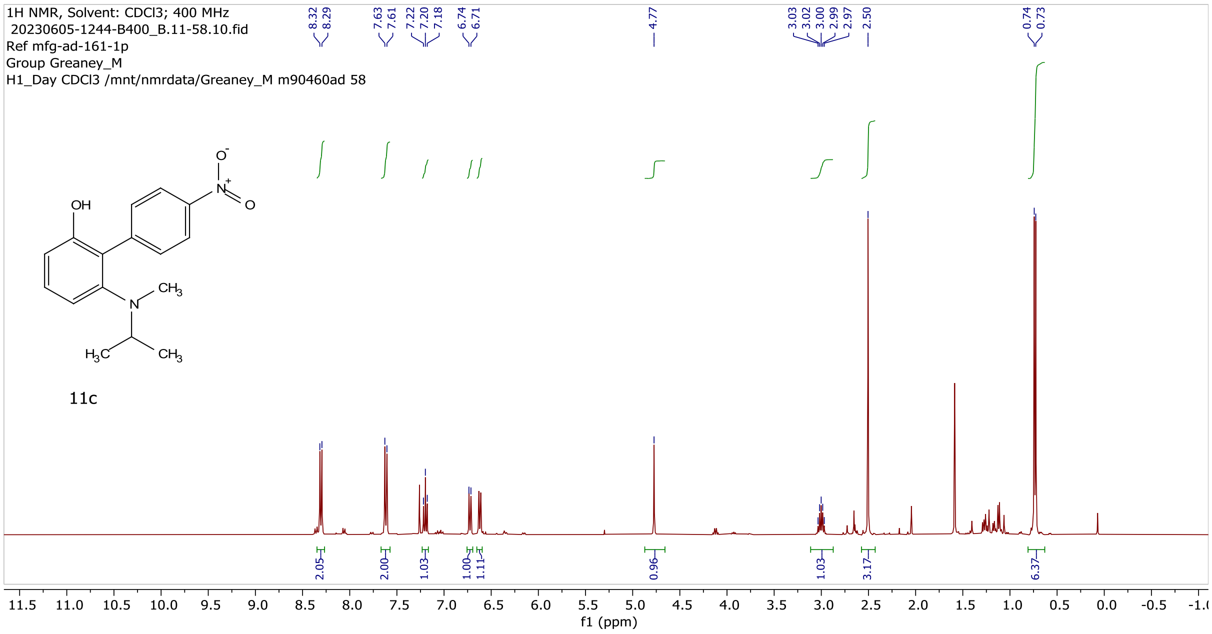
1H NMR, Solvent: CDCl3; 400 MHz
20230605-1244-B400_B.11-58.10.fid
Ref mfg-ad-161-1p
Group Greaney_M
H1_Day CDCl3 /mnt/nmrdata/Greaney_M m90460ad 58
8.32
8.29
7.63
7.61
7.22
7.20
7.18
6.74
6.71
4.77
3.03
3.02
3.00
2.99
2.97
2.50
0.74
0.73
OH
O-
N+
O
N
CH3
H3C
CH3
11c
2.05
2.00
1.03
1.00
1.11
0.96
1.03
3.17
6.37
f1 (ppm)

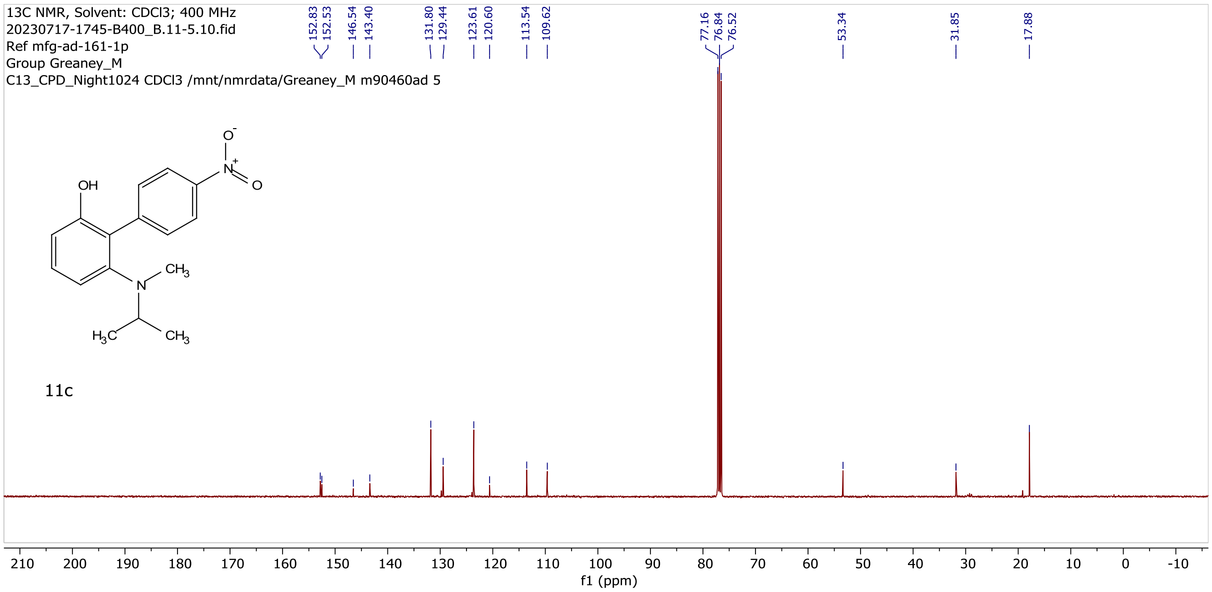
13C NMR, Solvent: CDCl3; 400 MHz
20230717-1745-B400_B.11-5.10.fid
Ref mfg-ad-161-1p
Group Greaney_M
C13_CPD_Night1024 CDCl3 /mnt/nmrdata/Greaney_M m90460ad 5
152.83
152.53
146.54
143.40
131.80
129.44
123.61
120.60
113.54
109.62
77.16
76.84
76.52
53.34
31.85
17.88
OH
N
CH3
H3C
CH3
11c
f1 (ppm)

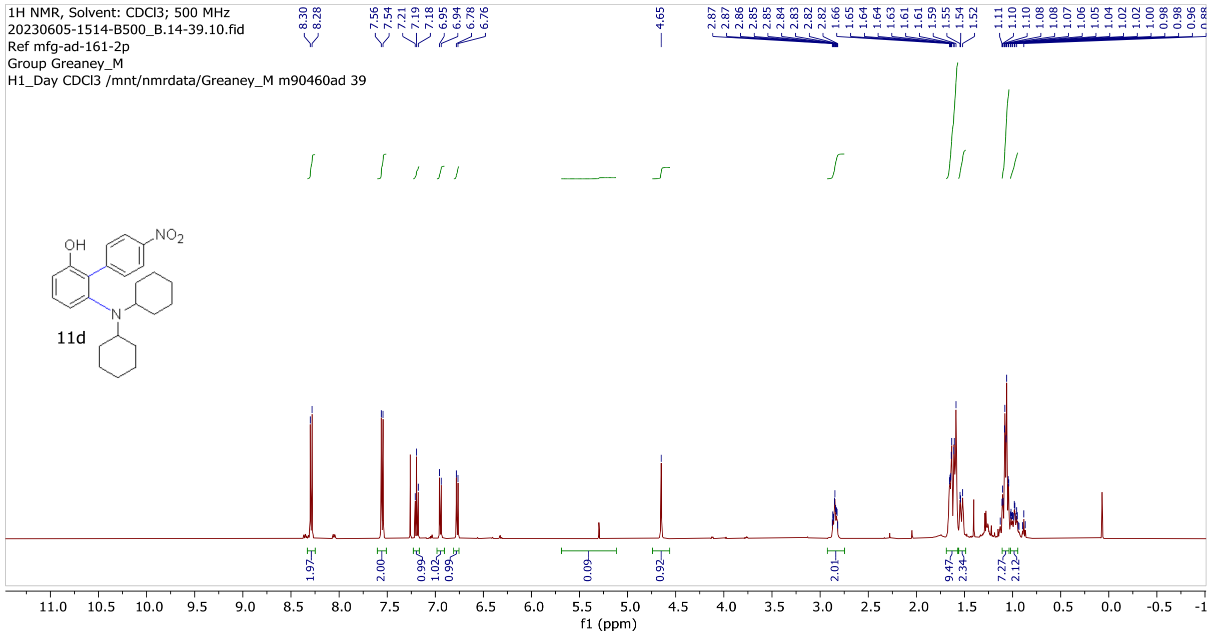
1H NMR, Solvent: CDCl3; 500 MHz
20230605-1514-B500_B.14-39.10.fid
Ref mfg-ad-161-2p
Group Greaney_M
H1_Day CDCl3 /mnt/nmrdata/Greaney_M m90460ad 39
OH
NO2
N
11d
1.97
2.00
0.99
1.02
0.99
0.09
0.92
2.01
9.47
2.34
7.27
2.12
f1 (ppm)

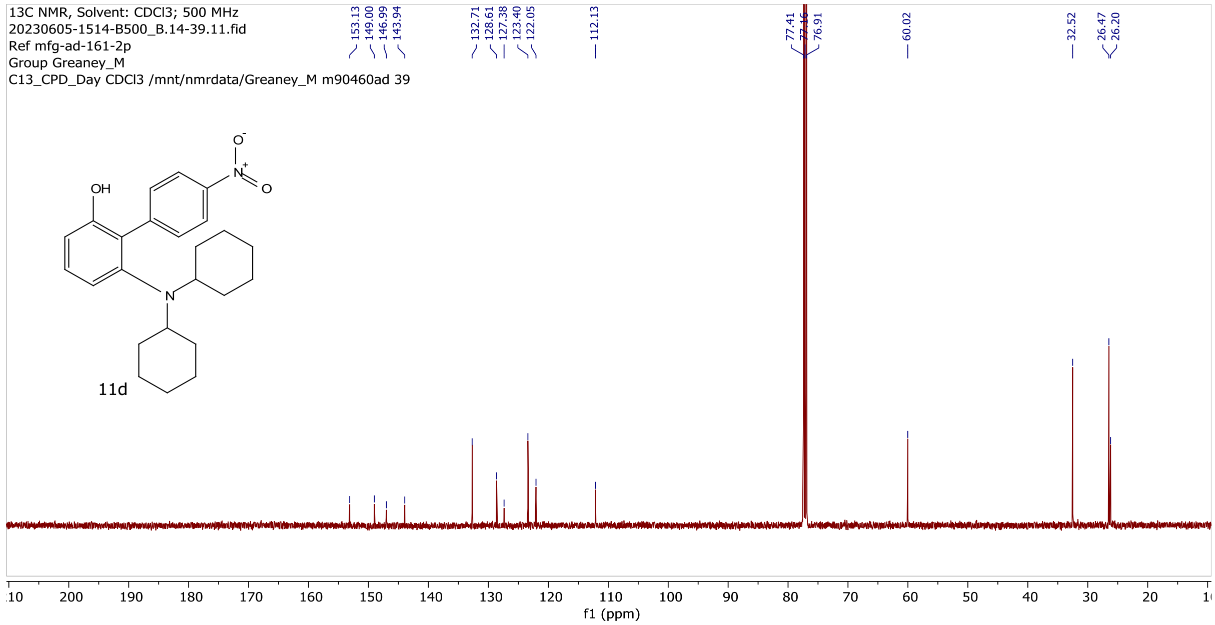
13C NMR, Solvent: CDCl3; 500 MHz
20230605-1514-B500_B.14-39.11.fid
Ref mfg-ad-161-2p
Group Greaney_M
C13_CPD_Day CDCl3 /mnt/nmrdata/Greaney_M m90460ad 39
11d
f1 (ppm)

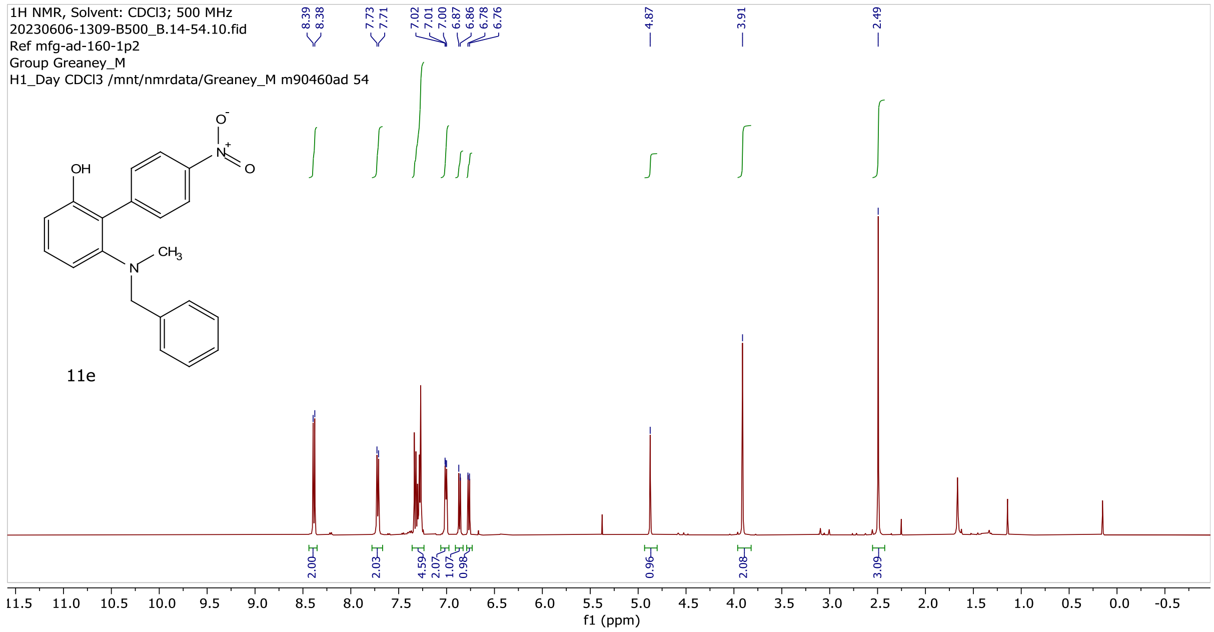
1H NMR, Solvent: CDCl3; 500 MHz
20230606-1309-B500_B.14-54.10.fid
Ref mfg-ad-160-1p2
Group Greaney_M
H1_Day CDCl3 /mnt/nmrdata/Greaney_M m90460ad 54
11e
f1 (ppm)

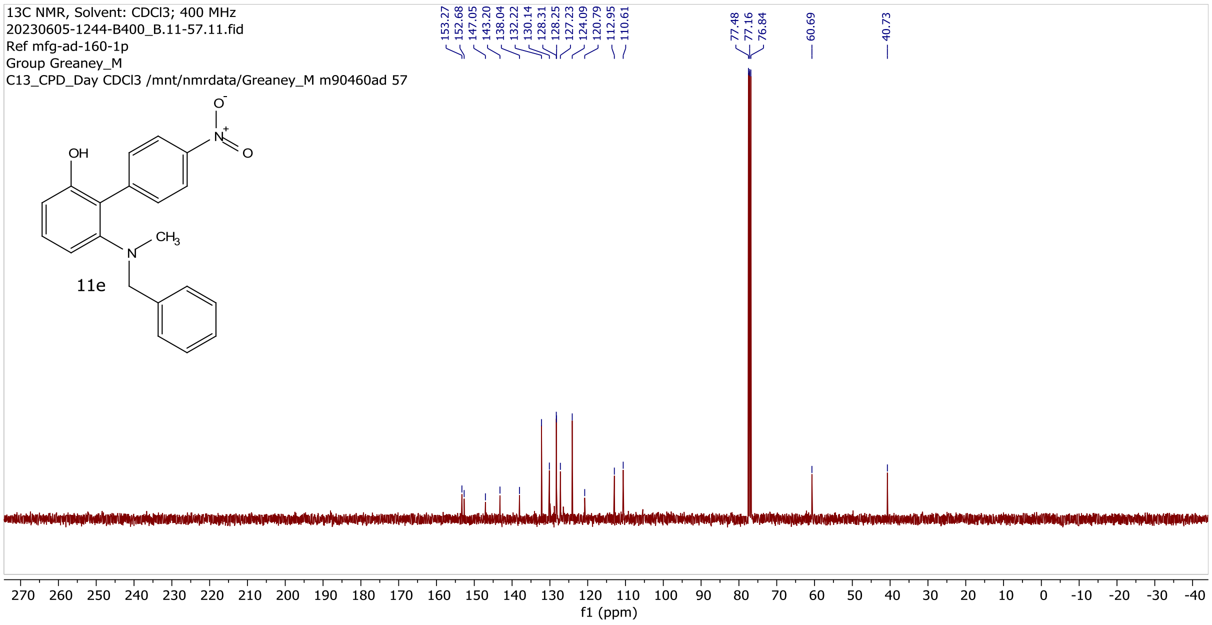
13C NMR, Solvent: CDCl3; 400 MHz
20230605-1244-B400_B.11-57.11.fid
Ref mfg-ad-160-1p
Group Greaney_M
C13_CPD_Day CDCl3 /mnt/nmrdata/Greaney_M m90460ad 57
153.27
152.68
147.05
143.20
138.04
132.22
130.14
128.31
128.25
127.23
124.09
120.79
112.95
110.61
77.48
77.16
76.84
60.69
40.73
11e
f1 (ppm)

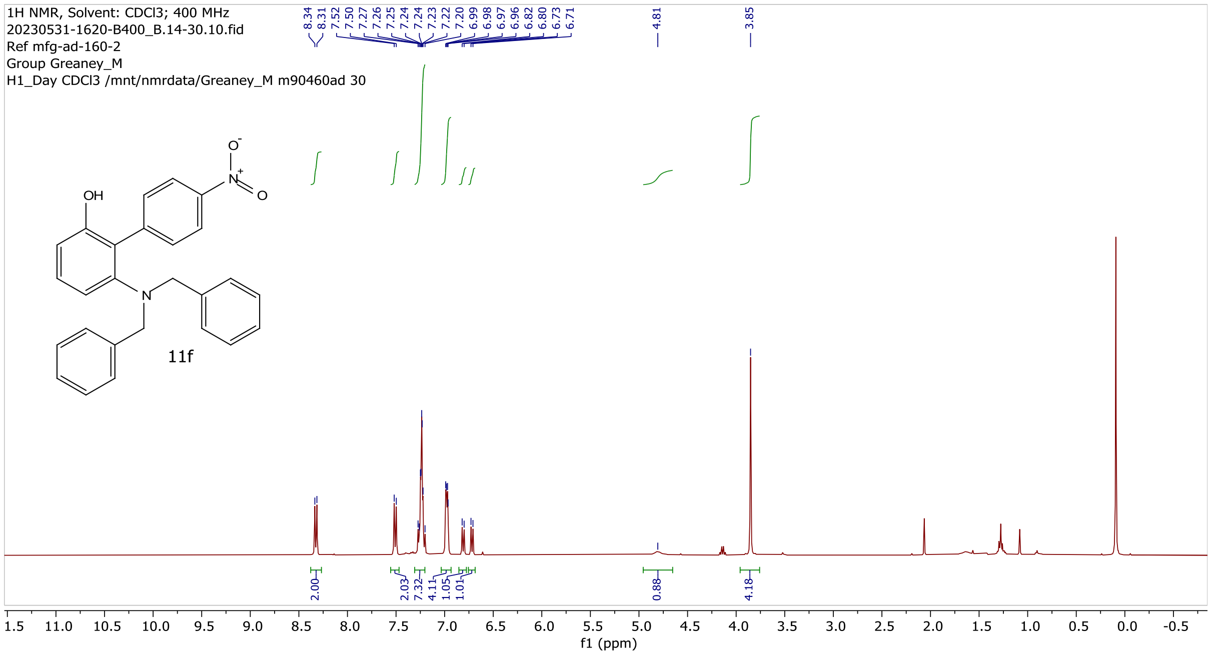
1H NMR, Solvent: CDCl3; 400 MHz
20230531-1620-B400_B.14-30.10.fid
Ref mfg-ad-160-2
Group Greaney_M
H1_Day CDCl3 /mnt/nmrdata/Greaney_M m90460ad 30
8.34
8.31
7.52
7.50
7.27
7.26
7.25
7.24
7.24
7.23
7.22
7.20
6.99
6.98
6.97
6.96
6.82
6.80
6.73
6.71
4.81
3.85
11f
2.00
2.03
7.32
4.11
1.05
1.01
0.88
4.18
f1 (ppm)

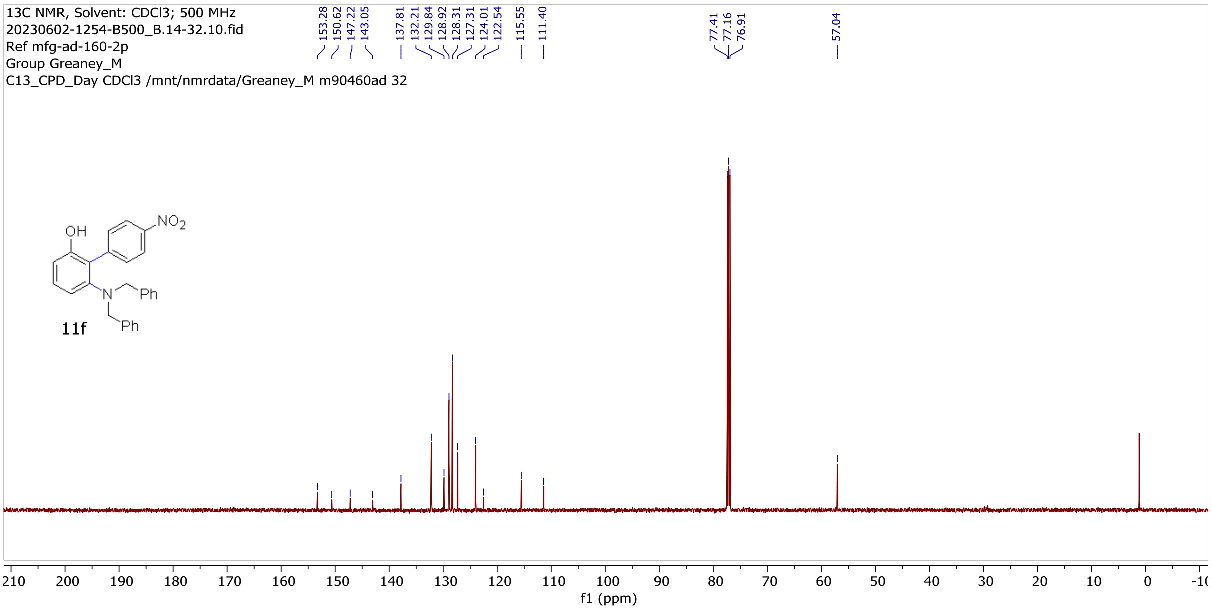
13C NMR, Solvent: CDCl3; 500 MHz
20230602-1254-B500_B.14-32.10.fid
Ref mfg-ad-160-2p
Group Greaney_M
C13_CPD_Day CDCl3 /mnt/nmrdata/Greaney_M m90460ad 32
153.28
150.62
147.22
143.05
137.81
132.21
129.84
128.92
128.31
127.31
124.01
122.54
115.55
111.40
77.41
77.16
76.91
57.04
OH
NO2
N
Ph
Ph
11f
f1 (ppm)

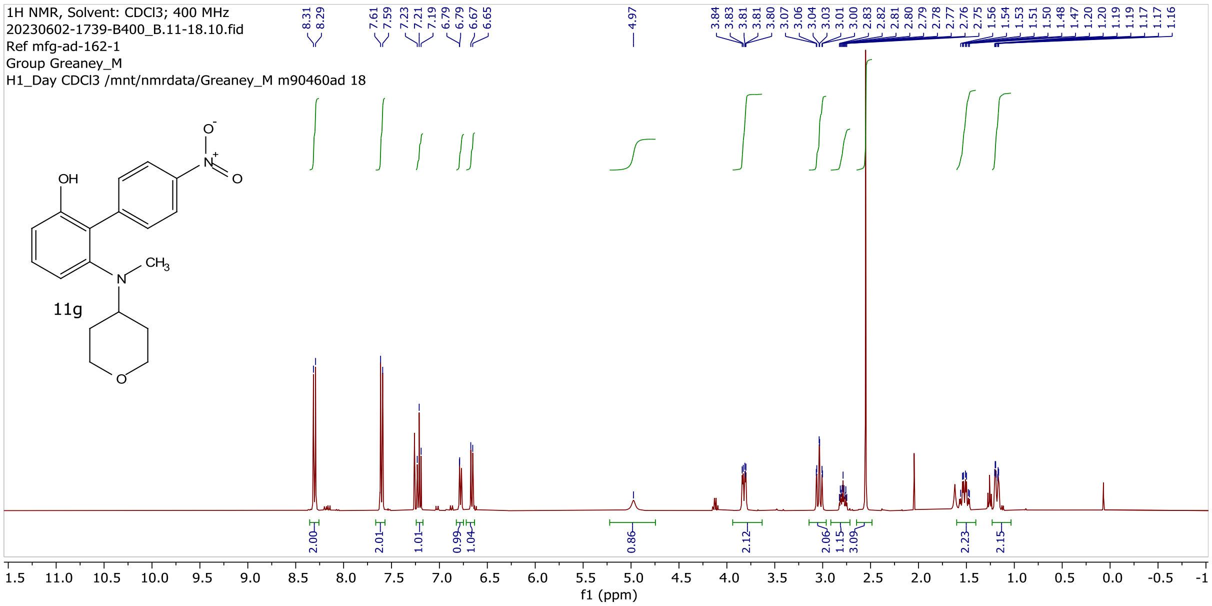
1H NMR, Solvent: CDCl3; 400 MHz
20230602-1739-B400_B.11-18.10.fid
Ref mfg-ad-162-1
Group Greaney_M
H1_Day CDCl3 /mnt/nmrdata/Greaney_M m90460ad 18
8.31
8.29
7.61
7.59
7.23
7.21
7.19
6.79
6.79
6.67
6.65
4.97
3.84
3.83
3.81
3.81
3.80
3.07
3.06
3.04
3.03
3.01
3.00
2.83
2.82
2.81
2.80
2.79
2.78
2.77
2.76
2.75
1.56
1.54
1.53
1.51
1.50
1.48
1.47
1.20
1.20
1.19
1.19
1.17
1.17
1.16
OH
N
CH3
O
11g
2.00
2.01
1.01
0.99
1.04
0.86
2.12
2.06
1.15
3.09
2.23
2.15
f1 (ppm)

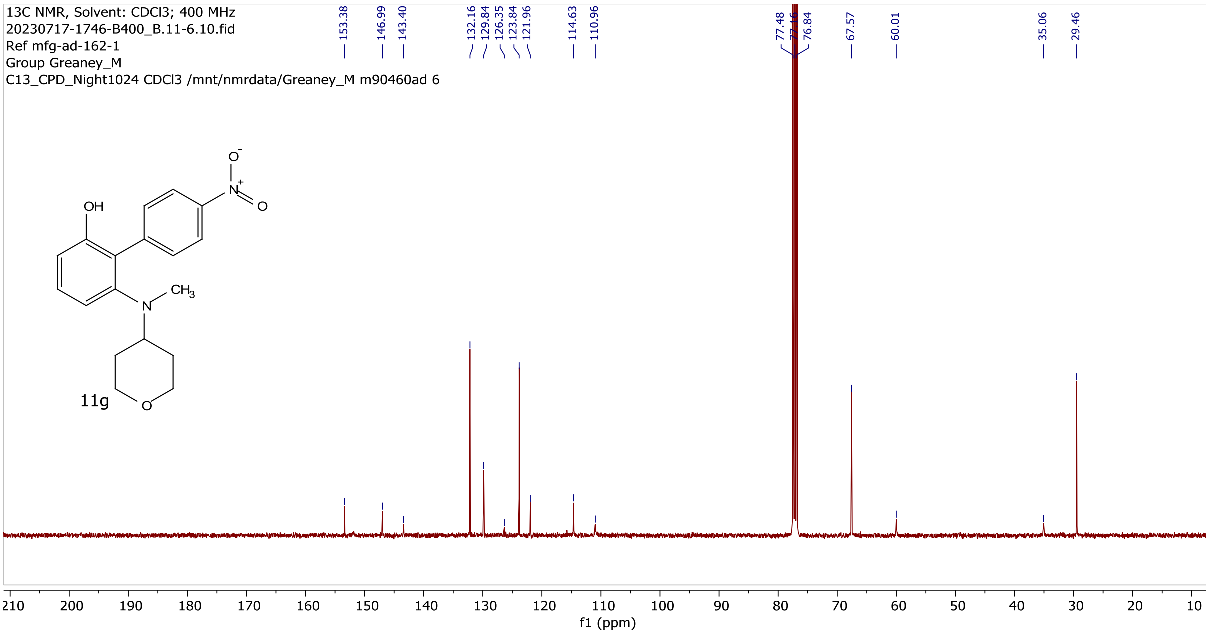
13C NMR, Solvent: CDCl3; 400 MHz
20230717-1746-B400_B.11-6.10.fid
Ref mfg-ad-162-1
Group Greaney_M
C13_CPD_Night1024 CDCl3 /mnt/nmrdata/Greaney_M m90460ad 6
153.38
146.99
143.40
132.16
129.84
126.35
123.84
121.96
114.63
110.96
77.48
77.16
76.84
67.57
60.01
35.06
29.46
OH
CH3
N
O
11g
210 200 190 180 170 160 150 140 130 120 110 100 90 80 70 60 50 40 30 20 10
f1 (ppm)

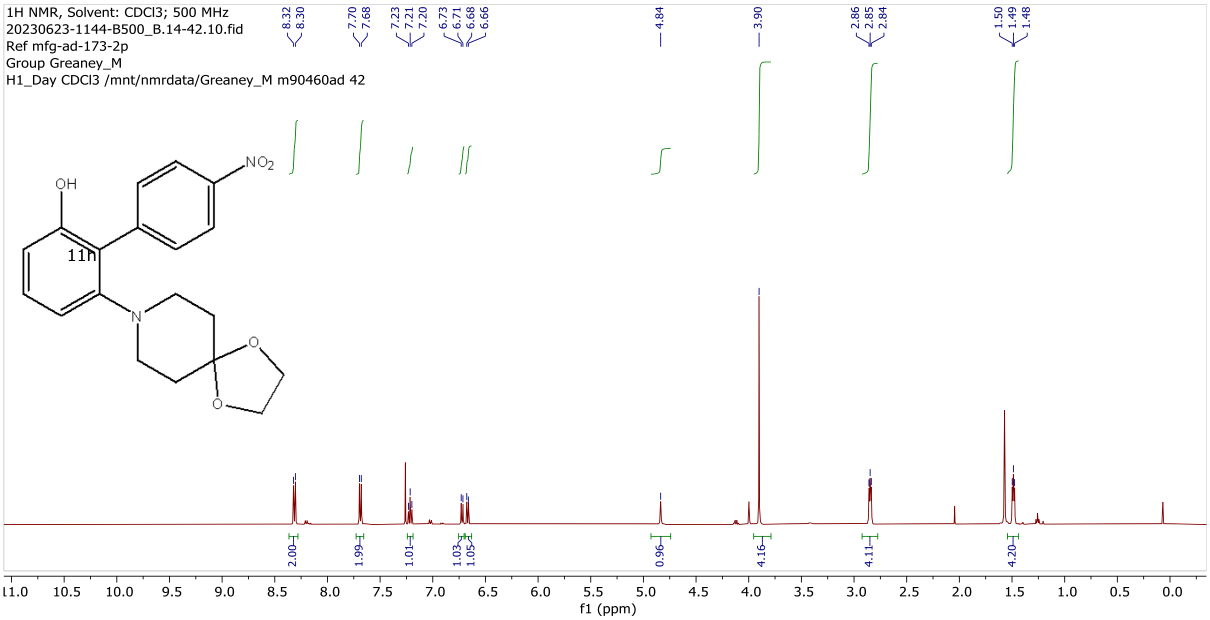
1H NMR, Solvent: CDCl3; 500 MHz
20230623-1144-B500_B.14-42.10.fid
Ref mfg-ad-173-2p
Group Greaney_M
H1_Day CDCl3 /mnt/nmrdata/Greaney_M m90460ad 42
8.32
8.30
7.70
7.68
7.23
7.21
7.20
6.73
6.71
6.68
6.66
4.84
3.90
2.86
2.85
2.84
1.50
1.49
1.48
OH
NO2
11h
N
O
2.00
1.99
1.01
1.03
1.05
0.96
4.16
4.11
4.20
11.0 10.5 10.0 9.5 9.0 8.5 8.0 7.5 7.0 6.5 6.0 5.5 5.0 4.5 4.0 3.5 3.0 2.5 2.0 1.5 1.0 0.5 0.0
f1 (ppm)

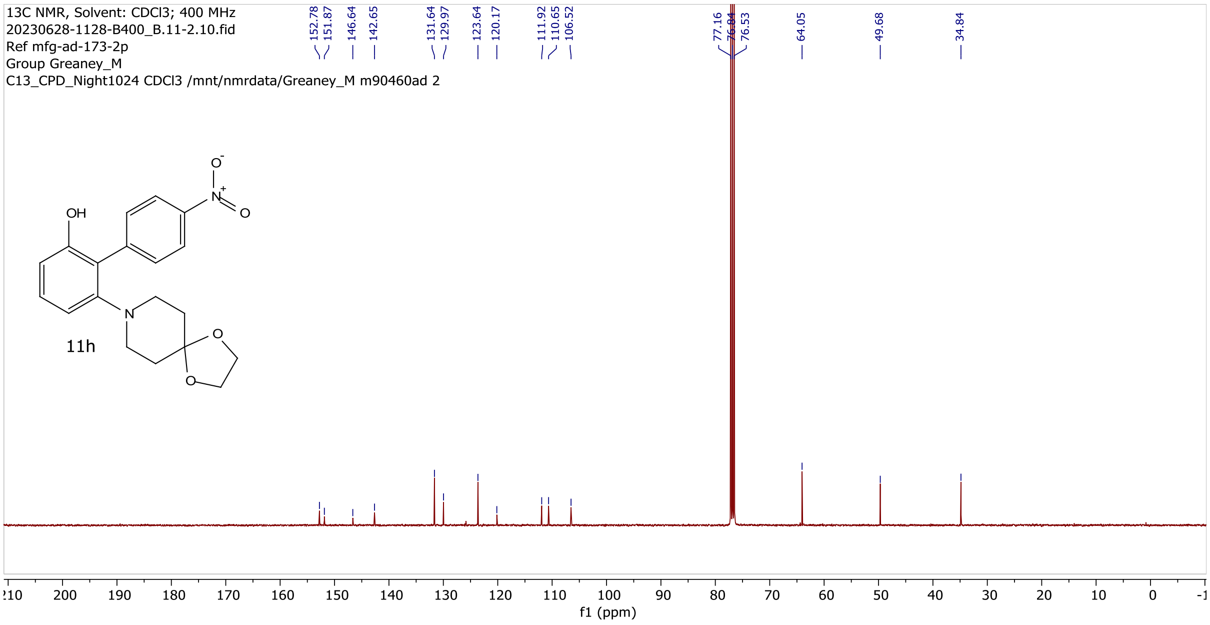
13C NMR, Solvent: CDCl3; 400 MHz
20230628-1128-B400_B.11-2.10.fid
Ref mfg-ad-173-2p
Group Greaney_M
C13_CPD_Night1024 CDCl3 /mnt/nmrdata/Greaney_M m90460ad 2
152.78
151.87
146.64
142.65
131.64
129.97
123.64
120.17
111.92
110.65
106.52
77.16
76.84
76.53
64.05
49.68
34.84
11h
f1 (ppm)

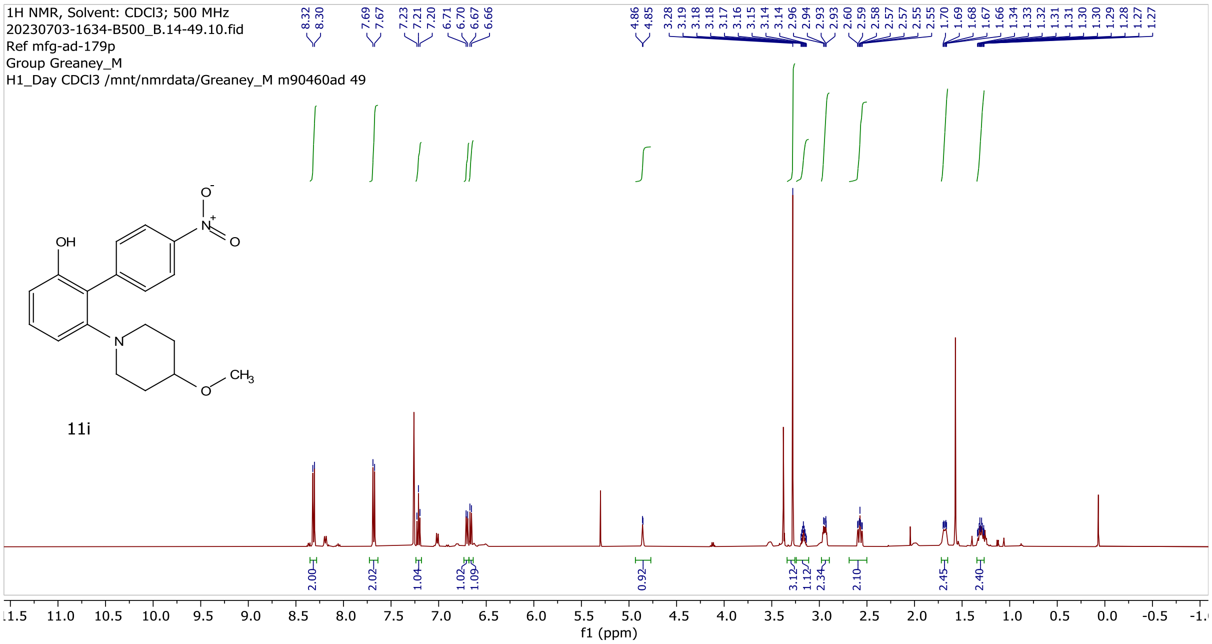
1H NMR, Solvent: CDCl3; 500 MHz
20230703-1634-B500_B.14-49.10.fid
Ref mfg-ad-179p
Group Greaney_M
H1_Day CDCl3 /mnt/nmrdata/Greaney_M m90460ad 49
8.32
8.30
7.69
7.67
7.23
7.21
7.20
6.71
6.70
6.67
6.66
4.86
4.85
3.28
3.19
3.18
3.18
3.17
3.16
3.15
3.14
3.14
2.96
2.94
2.93
2.93
2.60
2.59
2.58
2.57
2.57
2.55
2.55
1.70
1.69
1.68
1.67
1.66
1.34
1.33
1.32
1.31
1.31
1.30
1.30
1.29
1.28
1.27
1.27
11i
2.00
2.02
1.04
1.02
1.09
0.92
3.12
1.12
2.34
2.10
2.45
2.40
f1 (ppm)

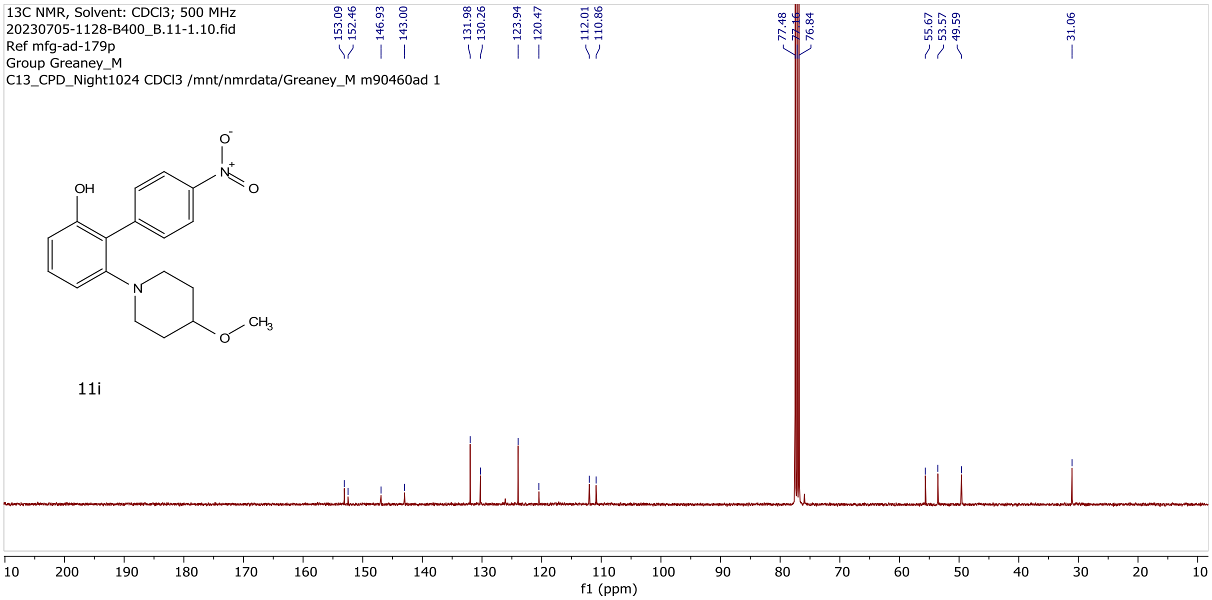

13C NMR, Solvent: CDCl3; 500 MHz
20230705-1128-B400_B.11-1.10.fid
Ref mfg-ad-179p
Group Greaney_M
C13_CPD_Night1024 CDCl3 /mnt/nmrdata/Greaney_M m90460ad 1
153.09
152.46
146.93
143.00
131.98
130.26
123.94
120.47
112.01
110.86
77.48
76.84
55.67
53.57
49.59
31.06
OH
O-
N+
O
N
O
CH3
11i
f1 (ppm)

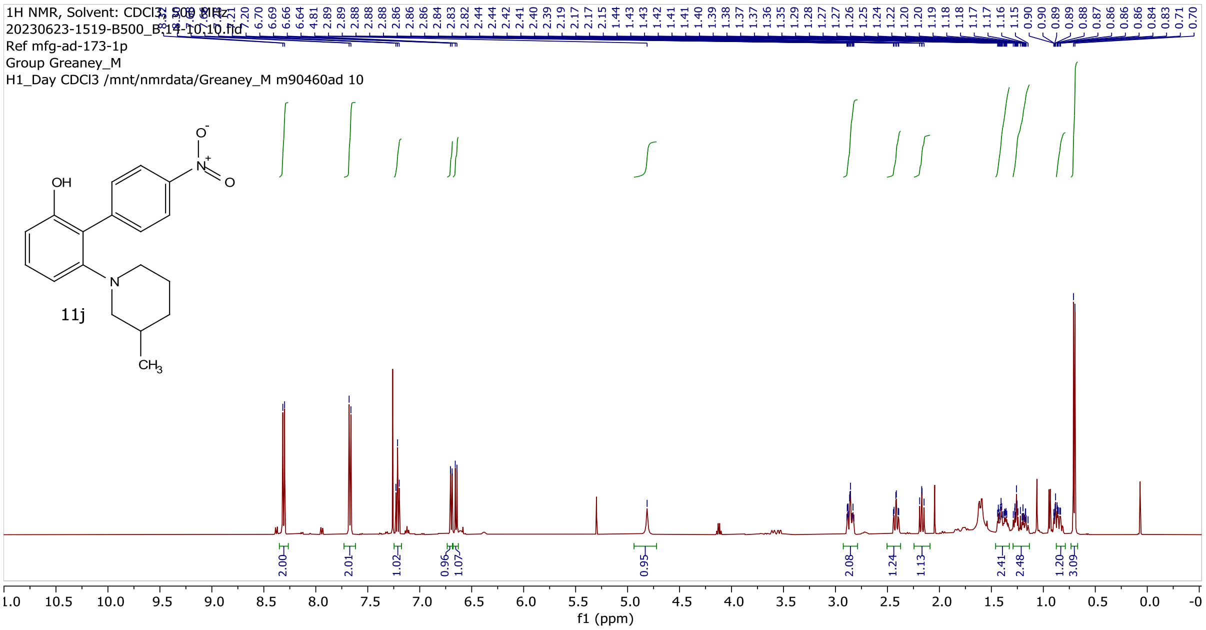

20230623-1519-B500_B.14-10.10.fid
Ref mfg-ad-173-1p
Group Greaney_M
H1_Day CDCl3 /mnt/nmrdata/Greaney_M m90460ad 10
OH
O-
N+
O
N
CH3
11j
2.00
2.01
1.02
0.96
1.07
0.95
2.08
1.24
1.13
2.41
2.48
1.20
3.09
f1 (ppm)

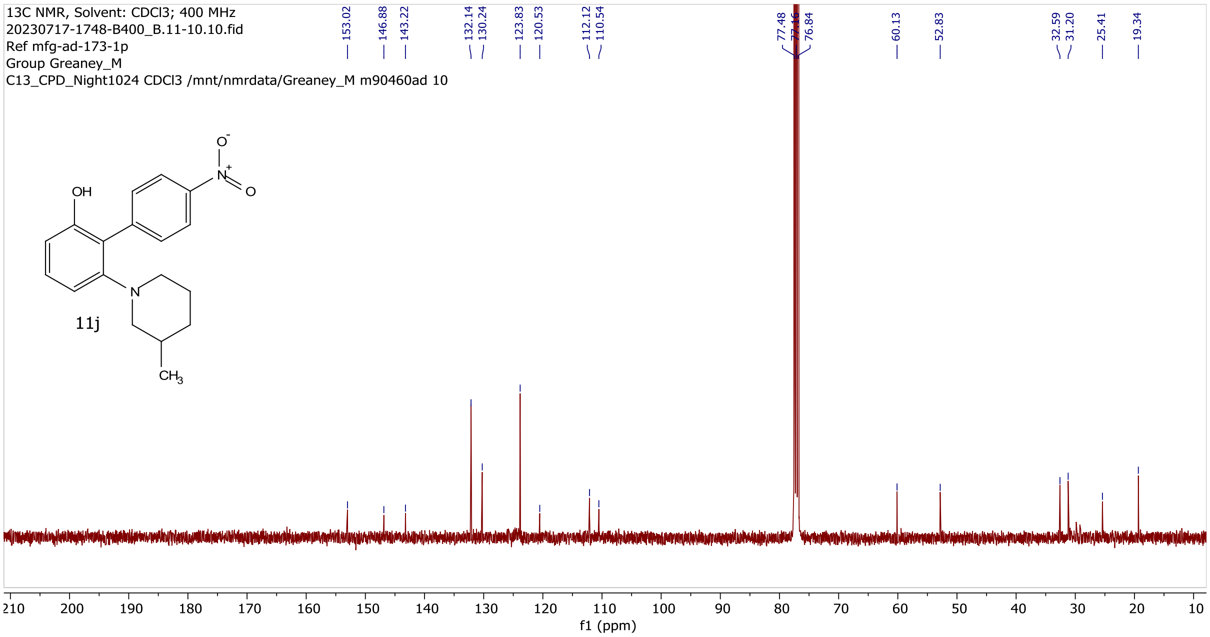

13C NMR, Solvent: CDCl3; 400 MHz
20230717-1748-B400_B.11-10.10.fid
Ref mfg-ad-173-1p
Group Greaney_M
C13_CPD_Night1024 CDCl3 /mnt/nmrdata/Greaney_M m90460ad 10
153.02
146.88
143.22
132.14
130.24
123.83
120.53
112.12
110.54
77.48
77.16
76.84
60.13
52.83
32.59
31.20
25.41
19.34
OH
N
11j
CH3
f1 (ppm)

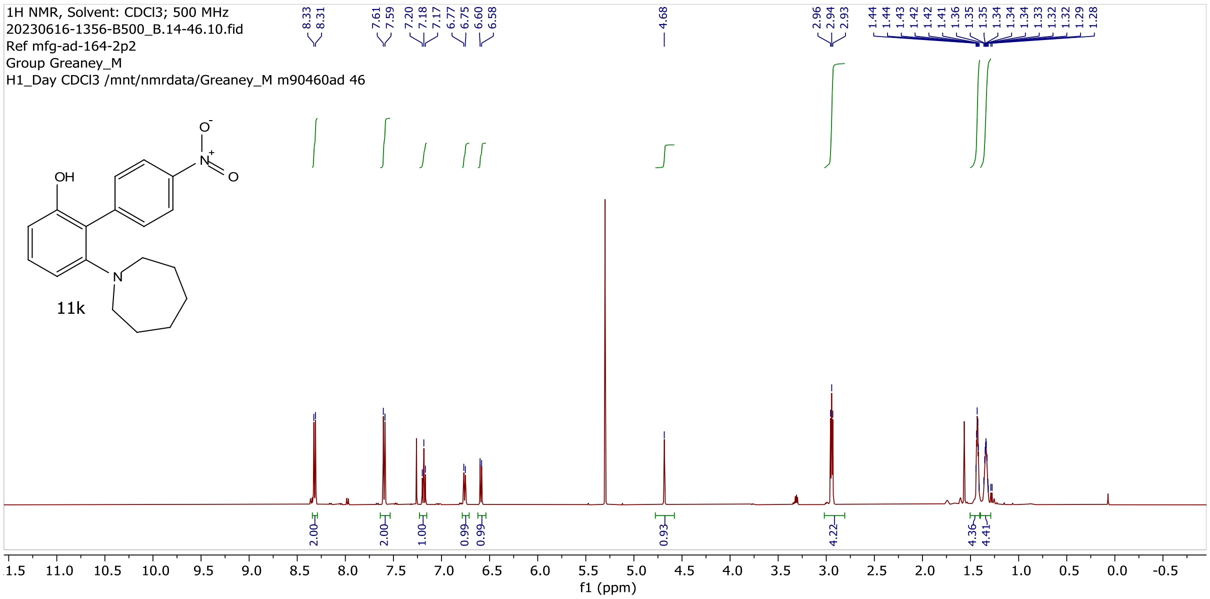

1H NMR, Solvent: CDCl3; 500 MHz
20230616-1356-B500_B.14-46.10.fid
Ref mfg-ad-164-2p2
Group Greaney_M
H1_Day CDCl3 /mnt/nmrdata/Greaney_M m90460ad 46
8.33
8.31
7.61
7.59
7.20
7.18
7.17
6.77
6.75
6.60
6.58
4.68
2.96
2.94
2.93
1.44
1.44
1.43
1.42
1.42
1.41
1.36
1.35
1.35
1.34
1.34
1.34
1.33
1.32
1.32
1.29
1.28
OH
N
11k
2.00
2.00
1.00
0.99
0.99
0.93
4.22
4.36
4.41
f1 (ppm)

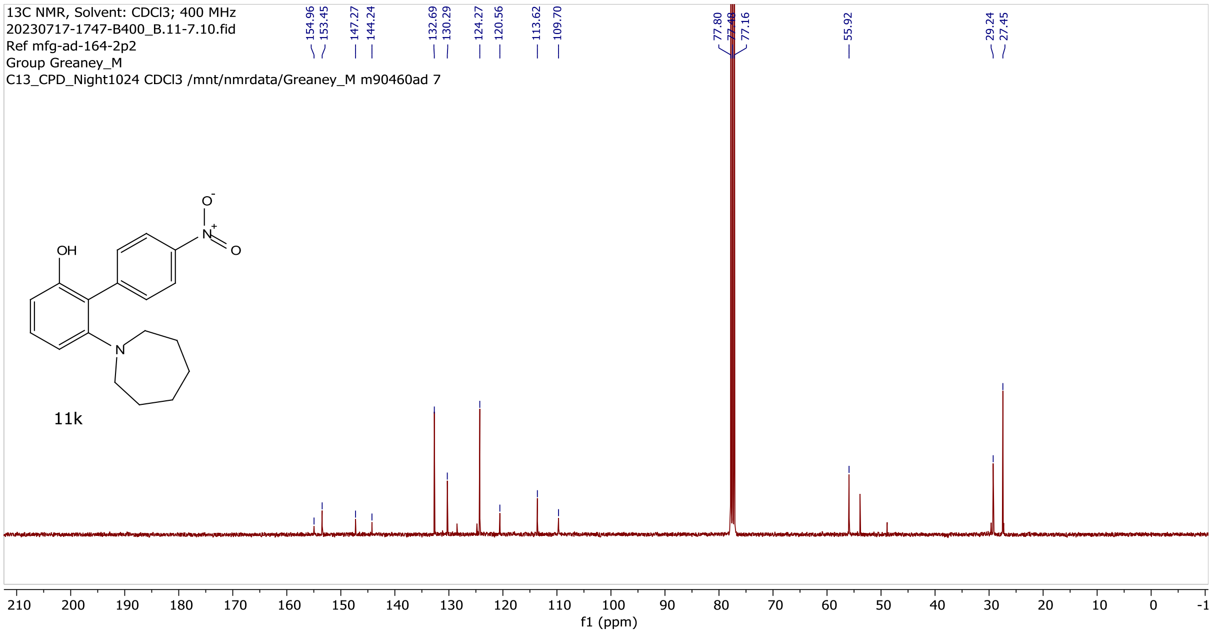
13C NMR, Solvent: CDCl3; 400 MHz
20230717-1747-B400_B.11-7.10.fid
Ref mfg-ad-164-2p2
Group Greaney_M
C13_CPD_Night1024 CDCl3 /mnt/nmrdata/Greaney_M m90460ad 7
154.96
153.45
147.27
144.24
132.69
130.29
124.27
120.56
113.62
109.70
77.80
77.16
55.92
29.24
27.45
OH
N
O
11k
210 200 190 180 170 160 150 140 130 120 110 100 90 80 70 60 50 40 30 20 10 0 -1
f1 (ppm)

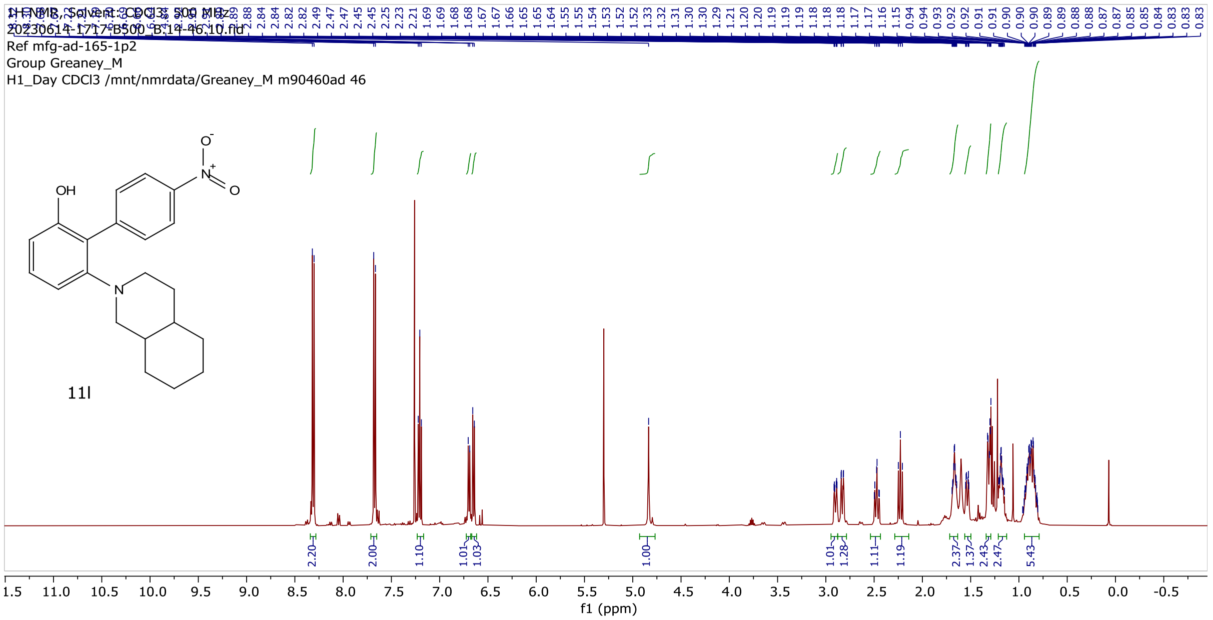
Ref mfg-ad-165-1p2
Group Greaney_M
H1_Day CDCl3 /mnt/nmrdata/Greaney_M m90460ad 46
2.88 2.84 2.84 2.82 2.82 2.49 2.47 2.47 2.45 2.45 2.25 2.23 2.21 1.69 1.69 1.68 1.68 1.67 1.67 1.66 1.65 1.65 1.64 1.55 1.55 1.54 1.53 1.53 1.52 1.52 1.33 1.32 1.31 1.30 1.30 1.29 1.21 1.20 1.19 1.19 1.19 1.18 1.18 1.18 1.17 1.17 1.16 1.15 0.94 0.94 0.93 0.92 0.92 0.91 0.91 0.90 0.90 0.90 0.89 0.89 0.88 0.88 0.87 0.87 0.85 0.85 0.84 0.83 0.83 0.83
OH
N
O
11l
2.20
2.00
1.10
1.01
1.03
1.00
1.01
1.28
1.11
1.19
2.37
1.37
2.43
2.47
5.43
1.5 11.0 10.5 10.0 9.5 9.0 8.5 8.0 7.5 7.0 6.5 6.0 5.5 5.0 4.5 4.0 3.5 3.0 2.5 2.0 1.5 1.0 0.5 0.0 -0.5
f1 (ppm)

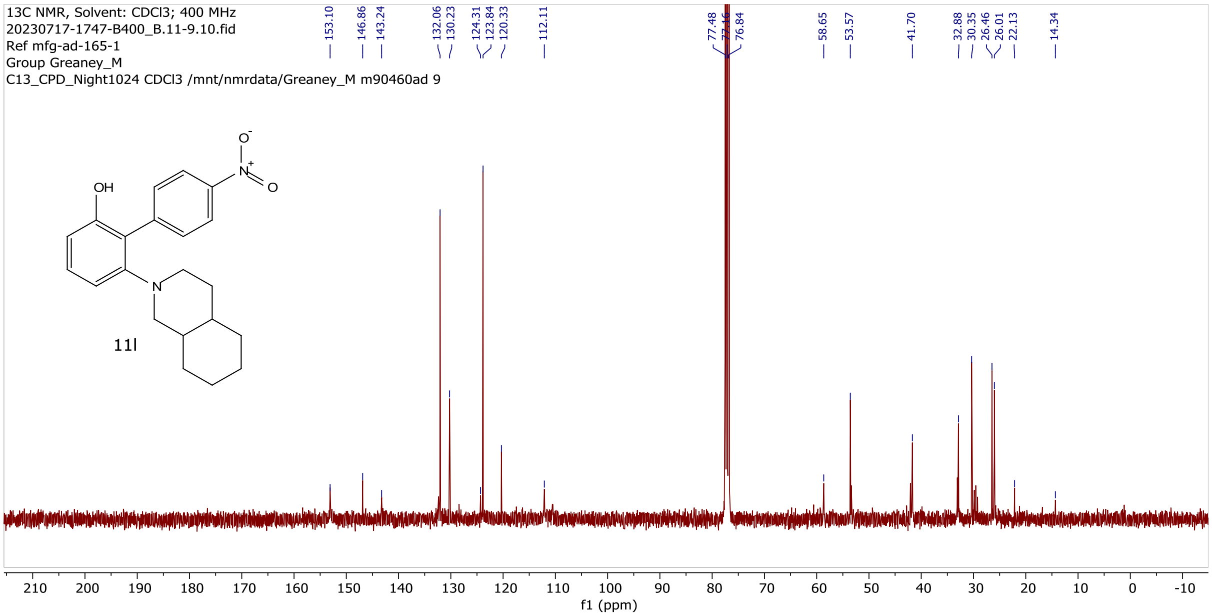

13C NMR, Solvent: CDCl3; 400 MHz
20230717-1747-B400_B.11-9.10.fid
Ref mfg-ad-165-1
Group Greaney_M
C13_CPD_Night1024 CDCl3 /mnt/nmrdata/Greaney_M m90460ad 9
153.10
146.86
143.24
132.06
130.23
124.31
123.84
120.33
112.11
77.48
76.84
58.65
53.57
41.70
32.88
30.35
26.46
26.01
22.13
14.34
OH
N
O
210 200 190 180 170 160 150 140 130 120 110 100 90 80 70 60 50 40 30 20 10 0 -10
f1 (ppm)

11l

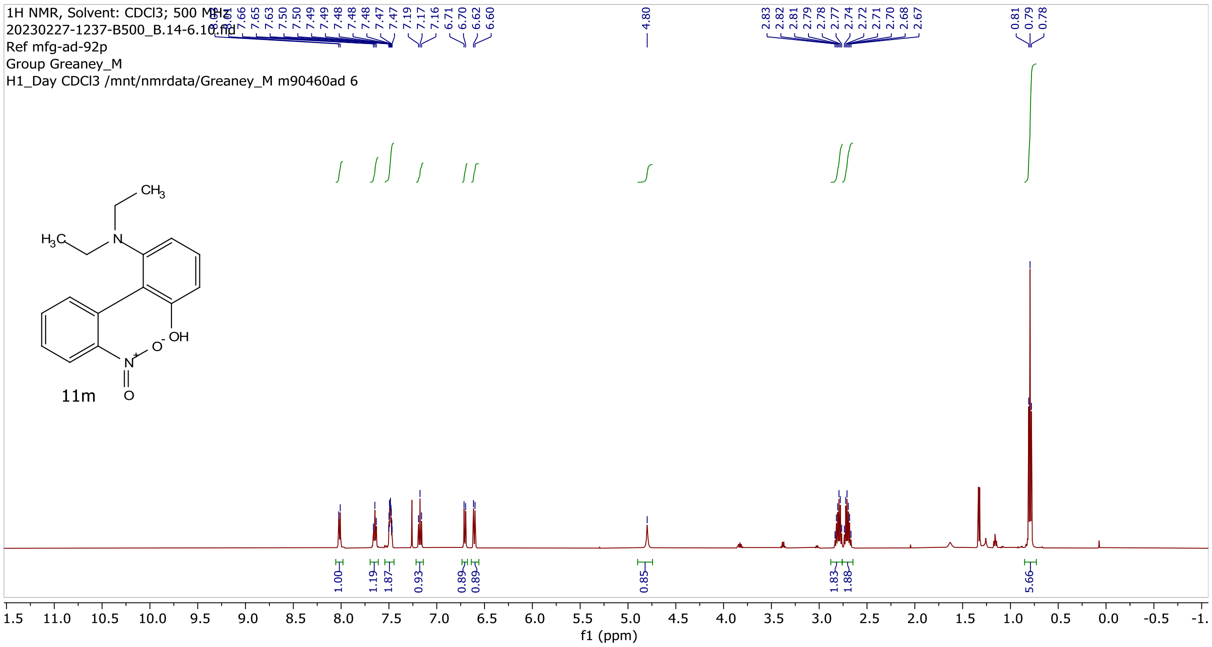

1H NMR, Solvent: CDCl3; 500 MHz
20230227-1237-B500_B.14-6.10.fid
Ref mfg-ad-92p
Group Greaney_M
H1_Day CDCl3 /mnt/nmrdata/Greaney_M m90460ad 6
7.66
7.65
7.63
7.50
7.50
7.49
7.49
7.48
7.48
7.48
7.47
7.47
7.19
7.17
7.16
6.71
6.70
6.62
6.60
4.80
2.83
2.82
2.81
2.79
2.78
2.77
2.74
2.72
2.71
2.70
2.68
2.67
0.81
0.79
0.78
CH3
H3C
N
OH
O
1.00
1.19
1.87
0.93
0.89
0.89
0.85
1.83
1.88
5.66
1.5 11.0 10.5 10.0 9.5 9.0 8.5 8.0 7.5 7.0 6.5 6.0 5.5 5.0 4.5 4.0 3.5 3.0 2.5 2.0 1.5 1.0 0.5 0.0 -0.5 -1.
f1 (ppm)

11m

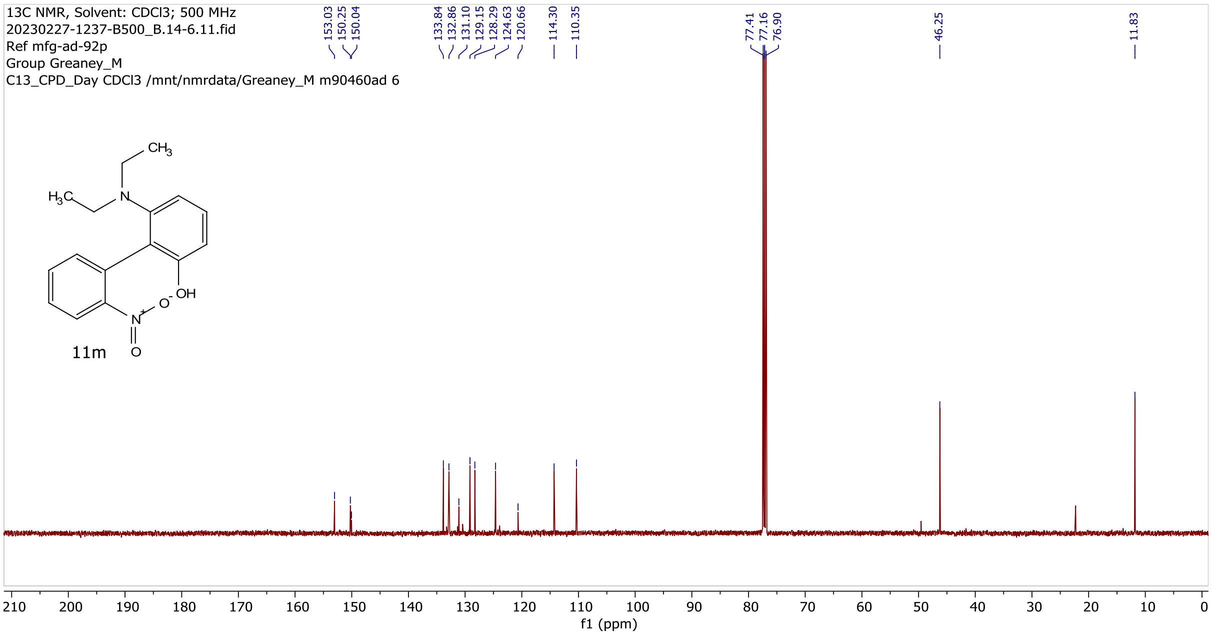
13C NMR, Solvent: CDCl3; 500 MHz
20230227-1237-B500_B.14-6.11.fid
Ref mfg-ad-92p
Group Greaney_M
C13_CPD_Day CDCl3 /mnt/nmrdata/Greaney_M m90460ad 6
153.03
150.25
150.04
133.84
132.86
131.10
129.15
128.29
124.63
120.66
114.30
110.35
77.41
77.16
76.90
46.25
11.83
CH3
H3C
N
OH
O-
N+
O
11m
f1 (ppm)

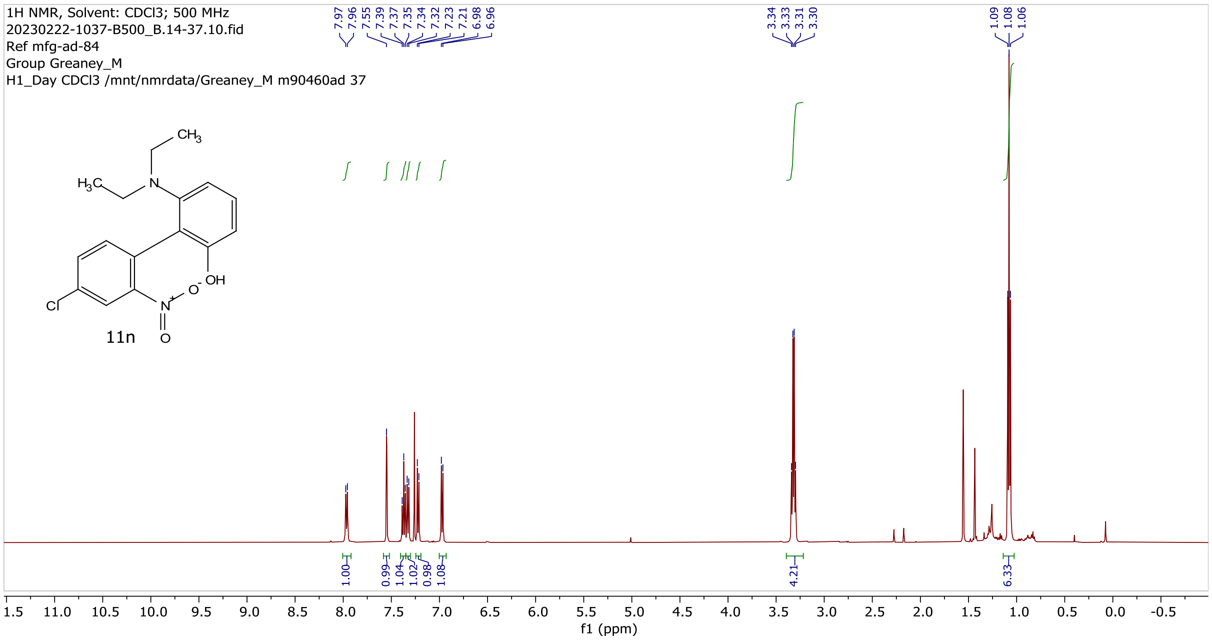
1H NMR, Solvent: CDCl3; 500 MHz
20230222-1037-B500_B.14-37.10.fid
Ref mfg-ad-84
Group Greaney_M
H1_Day CDCl3 /mnt/nmrdata/Greaney_M m90460ad 37
7.97
7.96
7.55
7.39
7.37
7.35
7.34
7.32
7.23
7.21
6.98
6.96
3.34
3.33
3.31
3.30
1.09
1.08
1.06
CH3
H3C
N
OH
O-
N+
Cl
O
11n
1.00
0.99
1.04
1.02
0.98
1.08
4.21
6.33
f1 (ppm)

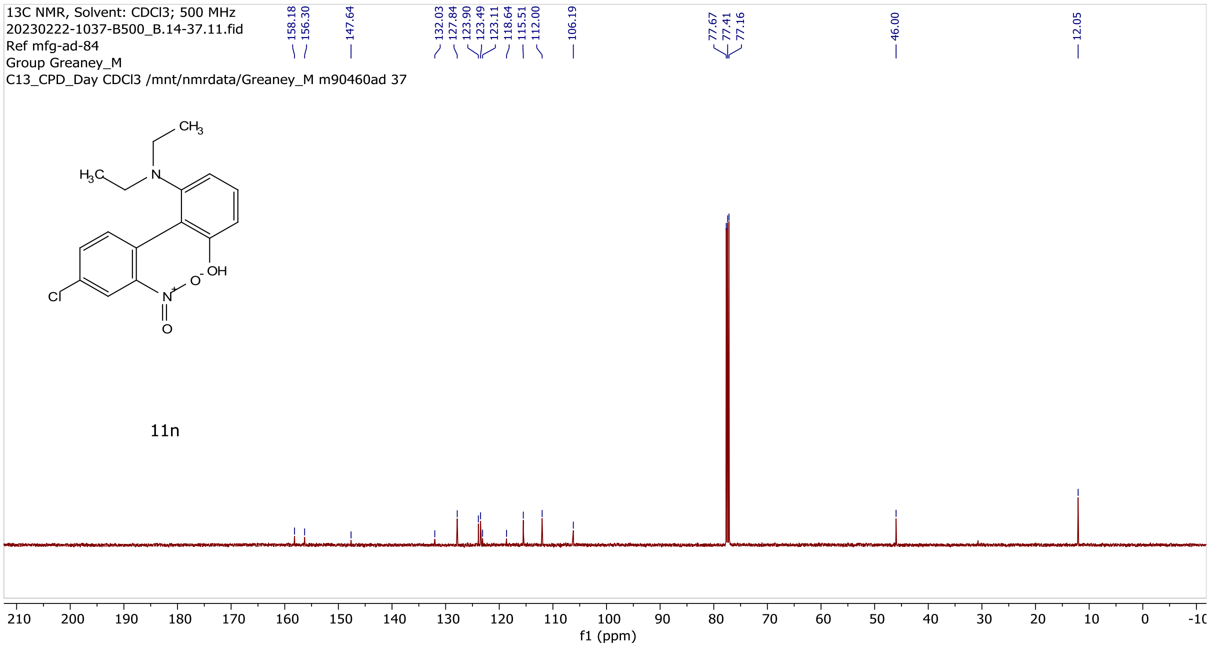
13C NMR, Solvent: CDCl3; 500 MHz
20230222-1037-B500_B.14-37.11.fid
Ref mfg-ad-84
Group Greaney_M
C13_CPD_Day CDCl3 /mnt/nmrdata/Greaney_M m90460ad 37
158.18
156.30
147.64
132.03
127.84
123.90
123.49
123.11
118.64
115.51
112.00
106.19
77.67
77.41
77.16
46.00
12.05
11n
f1 (ppm)

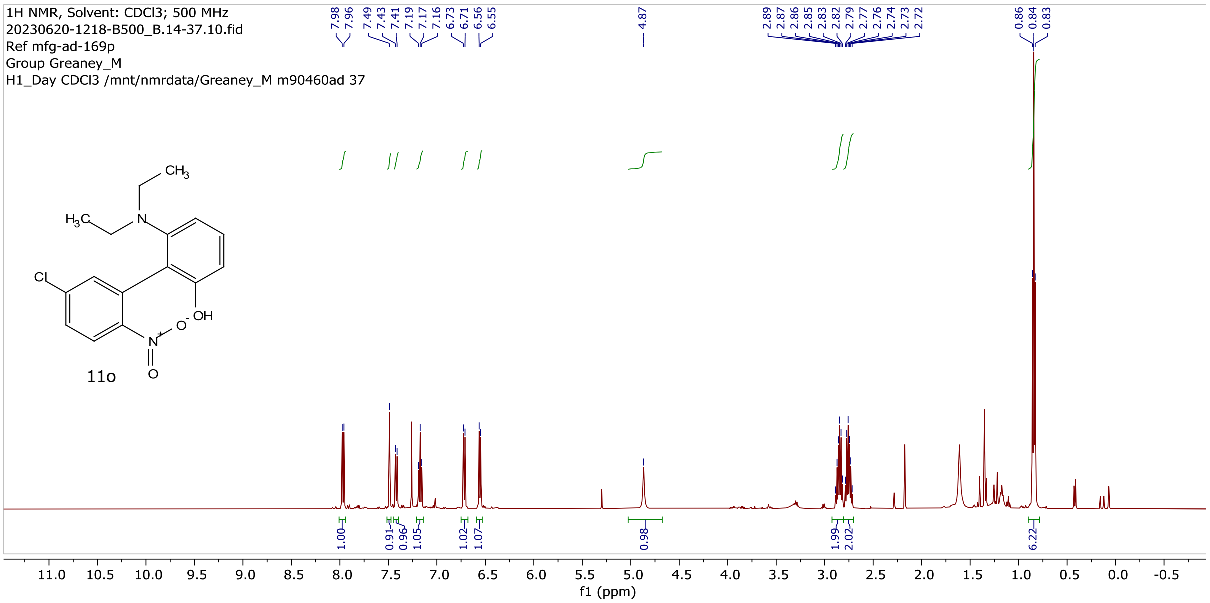
1H NMR, Solvent: CDCl3; 500 MHz
20230620-1218-B500_B.14-37.10.fid
Ref mfg-ad-169p
Group Greaney_M
H1_Day CDCl3 /mnt/nmrdata/Greaney_M m90460ad 37
7.98
7.96
7.49
7.43
7.41
7.19
7.17
7.16
6.73
6.71
6.56
6.55
4.87
2.89
2.87
2.86
2.85
2.83
2.82
2.79
2.77
2.76
2.74
2.73
2.72
0.86
0.84
0.83
11o
1.00
0.91
0.96
1.05
1.02
1.07
0.98
1.99
2.02
6.22
f1 (ppm)

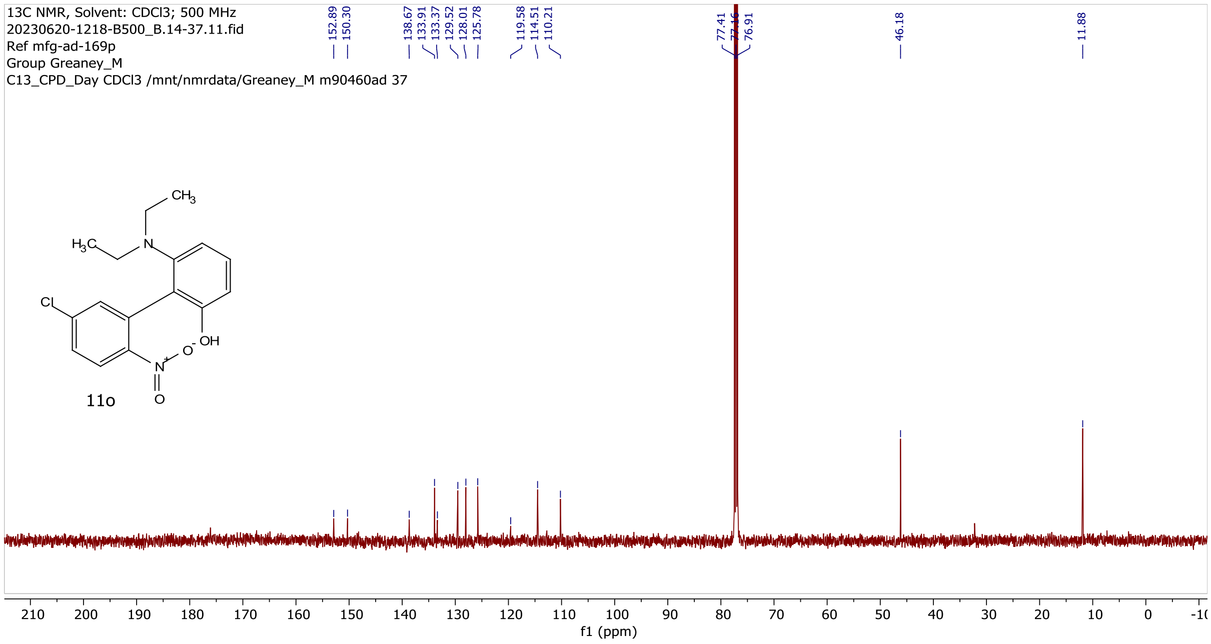
13C NMR, Solvent: CDCl3; 500 MHz
20230620-1218-B500_B.14-37.11.fid
Ref mfg-ad-169p
Group Greaney_M
C13_CPD_Day CDCl3 /mnt/nmrdata/Greaney_M m90460ad 37
152.89
150.30
138.67
133.91
133.37
129.52
128.01
125.78
119.58
114.51
110.21
77.41
76.91
46.18
11.88
11o
f1 (ppm)

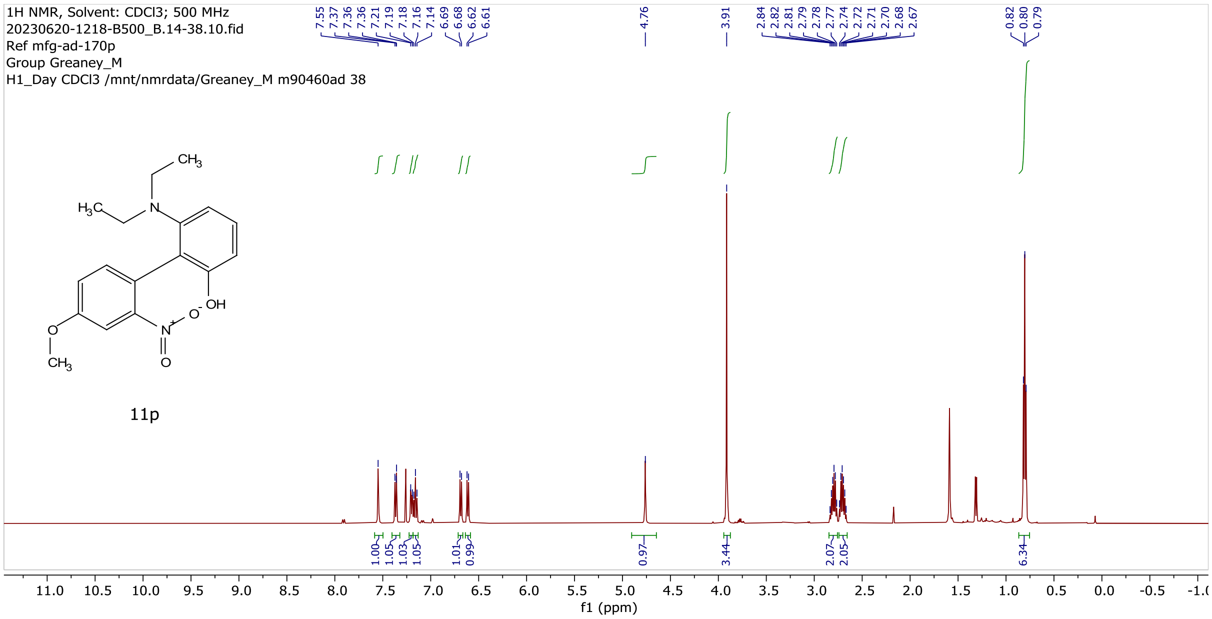
1H NMR, Solvent: CDCl3; 500 MHz
20230620-1218-B500_B.14-38.10.fid
Ref mfg-ad-170p
Group Greaney_M
H1_Day CDCl3 /mnt/nmrdata/Greaney_M m90460ad 38
7.55
7.37
7.36
7.21
7.19
7.18
7.16
7.14
6.69
6.68
6.62
6.61
4.76
3.91
2.84
2.82
2.81
2.79
2.78
2.77
2.74
2.72
2.71
2.70
2.68
2.67
0.82
0.80
0.79
11p
1.00
1.05
1.03
1.05
1.01
0.99
0.97
3.44
2.07
2.05
6.34
f1 (ppm)

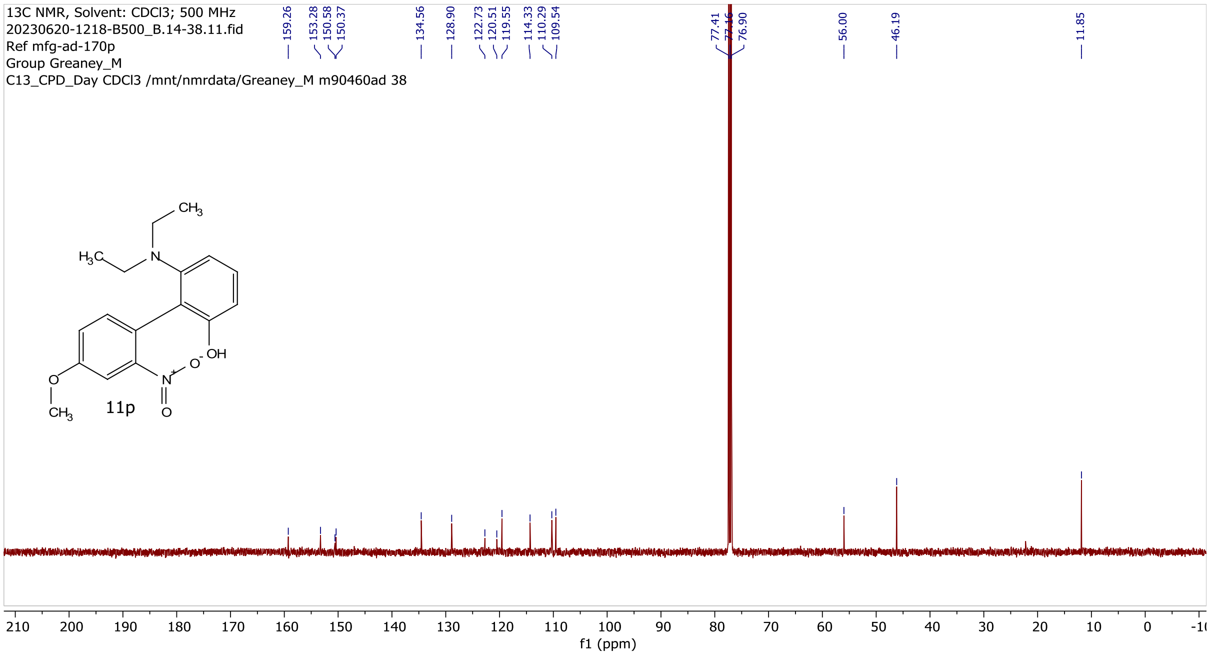
13C NMR, Solvent: CDCl3; 500 MHz
20230620-1218-B500_B.14-38.11.fid
Ref mfg-ad-170p
Group Greaney_M
C13_CPD_Day CDCl3 /mnt/nmrdata/Greaney_M m90460ad 38
159.26 153.28 150.58 150.37 134.56 128.90 122.73 120.51 119.55 114.33 110.29 109.54 77.41 77.16 76.90 56.00 46.19 11.85
11p
f1 (ppm)

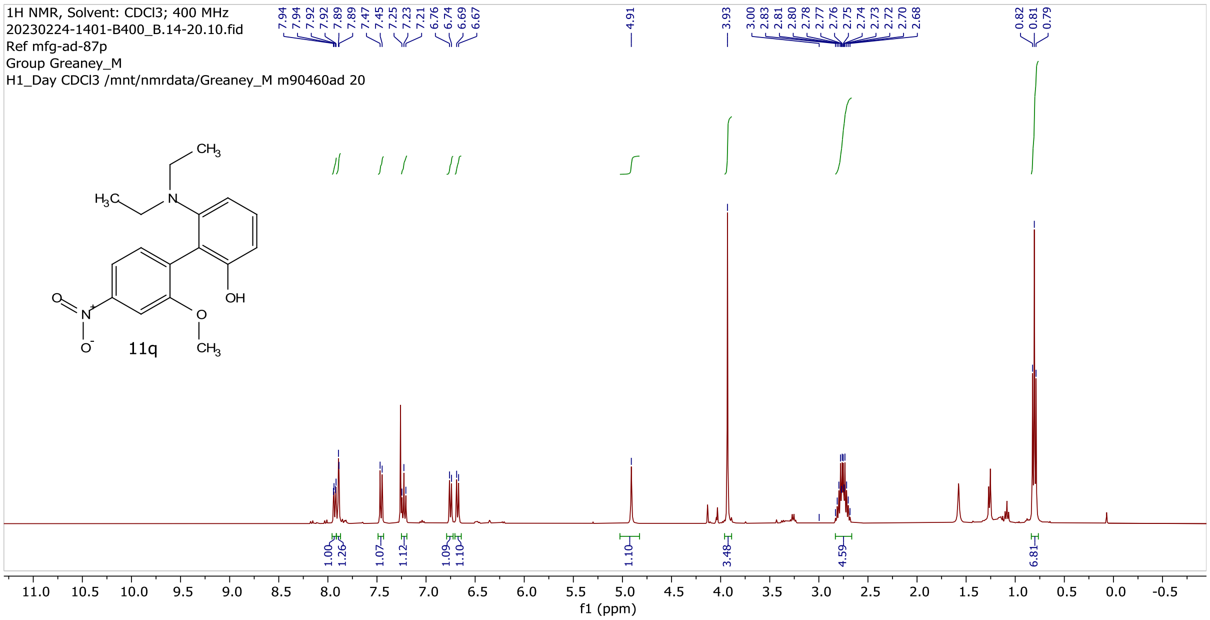
1H NMR, Solvent: CDCl3; 400 MHz
20230224-1401-B400_B.14-20.10.fid
Ref mfg-ad-87p
Group Greaney_M
H1_Day CDCl3 /mnt/nmrdata/Greaney_M m90460ad 20
7.94 7.94 7.92 7.92 7.89 7.47 7.45 7.25 7.23 7.21 6.76 6.74 6.69 6.67 4.91 3.93 3.00 2.83 2.81 2.80 2.78 2.77 2.76 2.75 2.74 2.73 2.72 2.70 2.68 0.82 0.81 0.79
11q
1.00 1.26 1.07 1.12 1.09 1.10 1.10 3.48 4.59 6.81
f1 (ppm)

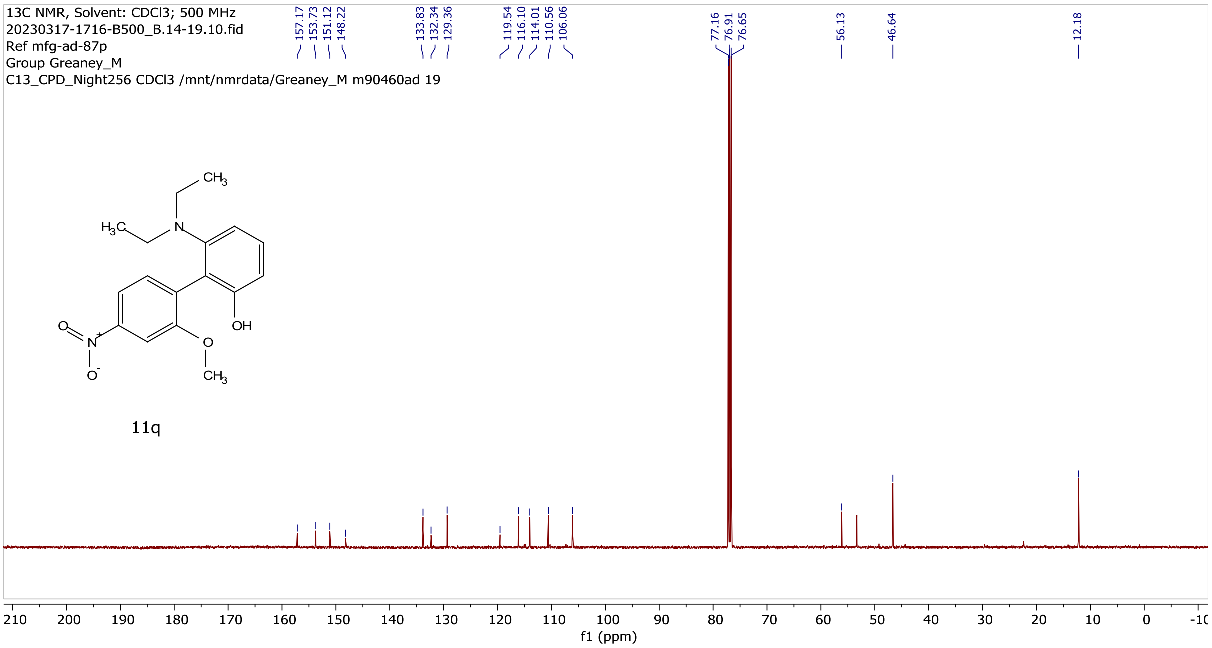
13C NMR, Solvent: CDCl3; 500 MHz
20230317-1716-B500_B.14-19.10.fid
Ref mfg-ad-87p
Group Greaney_M
C13_CPD_Night256 CDCl3 /mnt/nmrdata/Greaney_M m90460ad 19
157.17
153.73
151.12
148.22
133.83
132.34
129.36
119.54
116.10
114.01
110.56
106.06
77.16
76.91
76.65
56.13
46.64
12.18
11q
f1 (ppm)

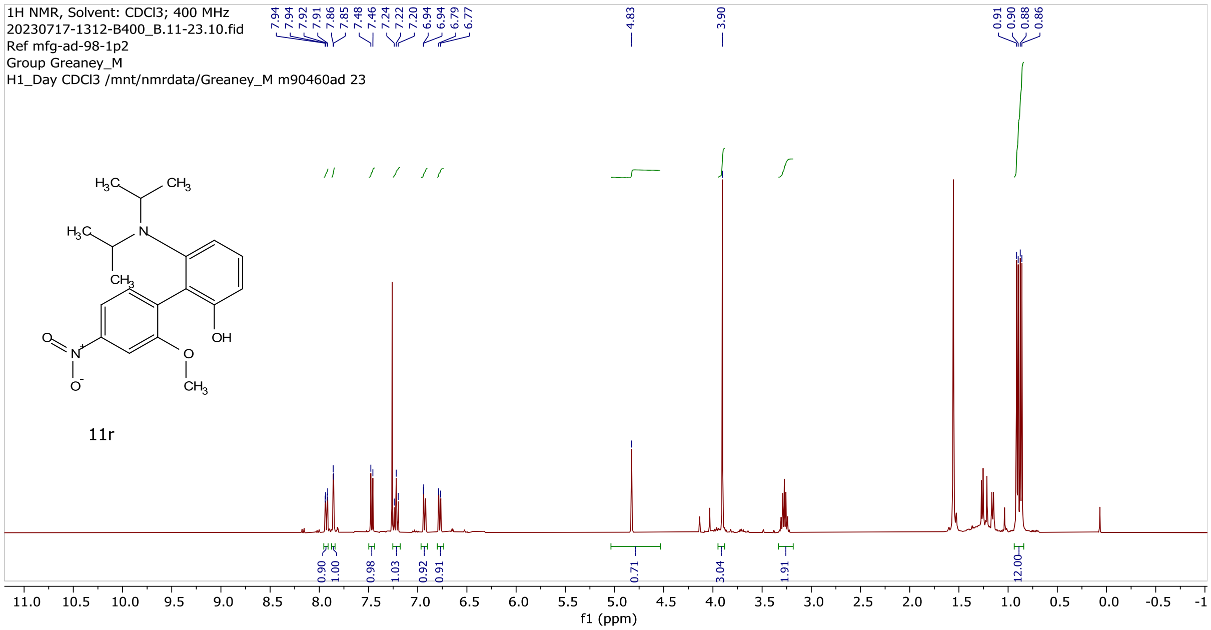
1H NMR, Solvent: CDCl3; 400 MHz
20230717-1312-B400_B.11-23.10.fid
Ref mfg-ad-98-1p2
Group Greaney_M
H1_Day CDCl3 /mnt/nmrdata/Greaney_M m90460ad 23
7.94
7.94
7.92
7.91
7.86
7.85
7.48
7.46
7.24
7.22
7.20
6.94
6.94
6.79
6.77
4.83
3.90
0.91
0.90
0.88
0.86
11r
0.90
1.00
0.98
1.03
0.92
0.91
0.71
3.04
1.91
12.00
f1 (ppm)

13C NMR, Solvent: CDCl3; 500 MHz
20230313-1410-B500_B.14-23.11.fid
Ref mfg-ad-98-1-p1
Group Greaney_M
C13_CPD_Day CDCl3 /mnt/nmrdata/Greaney_M m90460ad 23

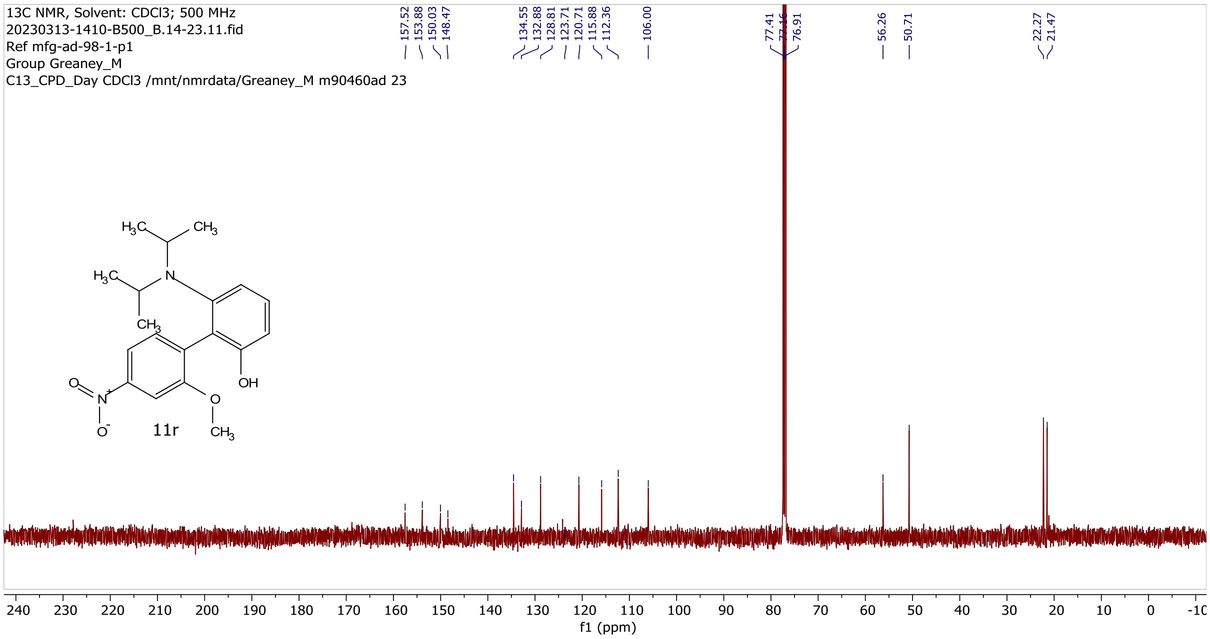

1H NMR, Solvent: CDCl3; 400 MHz
20230217-1643-B400_B.14-57.10.fid
Ref mfg-ad-77a
Group Greaney_M
H1_Day CDCl3 /mnt/nmrdata/Greaney_M m90460ad 57

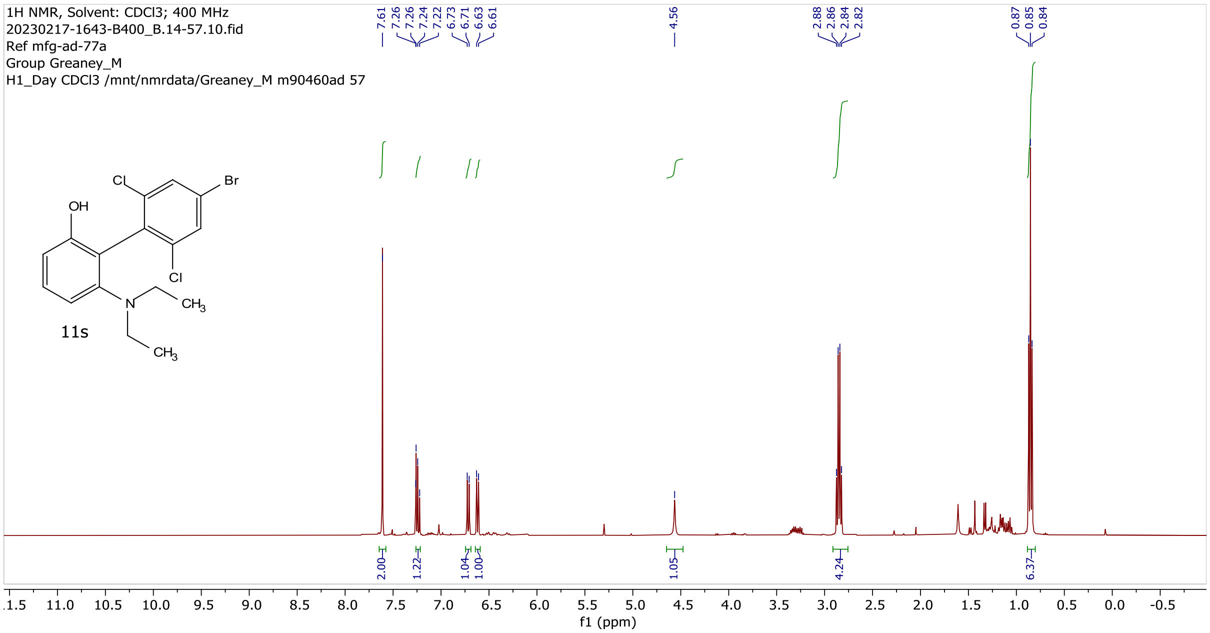

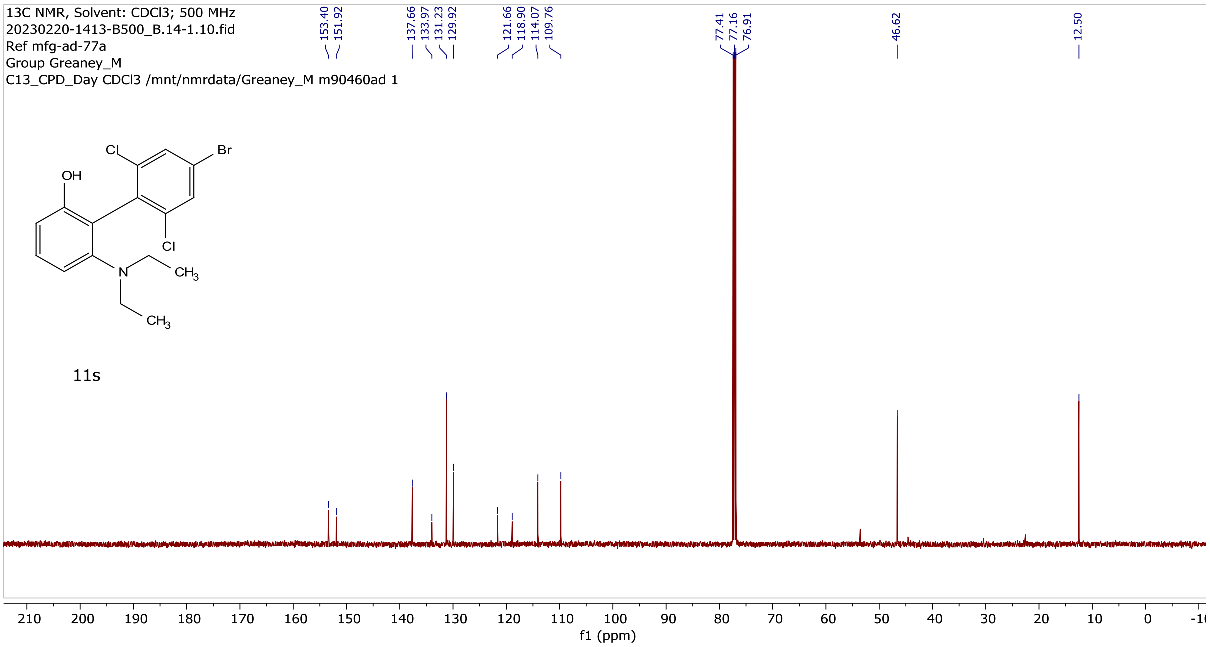
13C NMR, Solvent: CDCl3; 500 MHz
20230220-1413-B500_B.14-1.10.fid
Ref mfg-ad-77a
Group Greaney_M
C13_CPD_Day CDCl3 /mnt/nmrdata/Greaney_M m90460ad 1
153.40
151.92
137.66
133.97
131.23
129.92
121.66
118.90
114.07
109.76
77.41
77.16
76.91
46.62
12.50
OH
Cl
Br
Cl
N
CH3
CH3
11s
f1 (ppm)

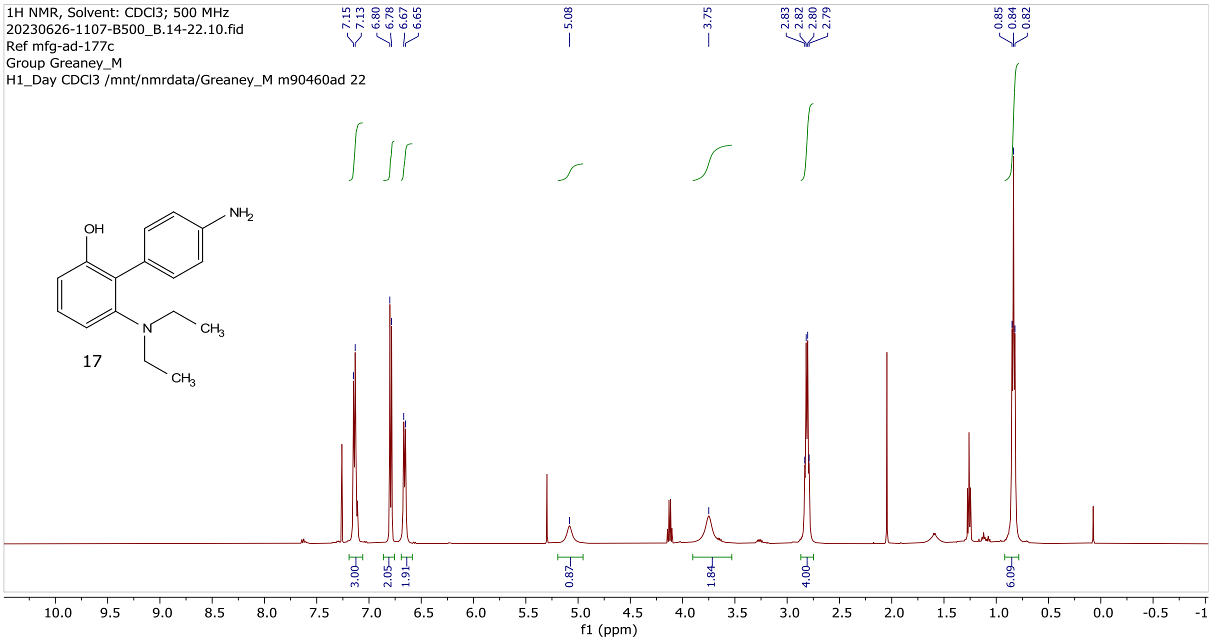
1H NMR, Solvent: CDCl3; 500 MHz
20230626-1107-B500_B.14-22.10.fid
Ref mfg-ad-177c
Group Greaney_M
H1_Day CDCl3 /mnt/nmrdata/Greaney_M m90460ad 22
7.15
7.13
6.80
6.78
6.67
6.65
5.08
3.75
2.83
2.82
2.80
2.79
0.85
0.84
0.82
OH
NH2
N
CH3
CH3
17
3.00
2.05
1.91
0.87
1.84
4.00
6.09
f1 (ppm)

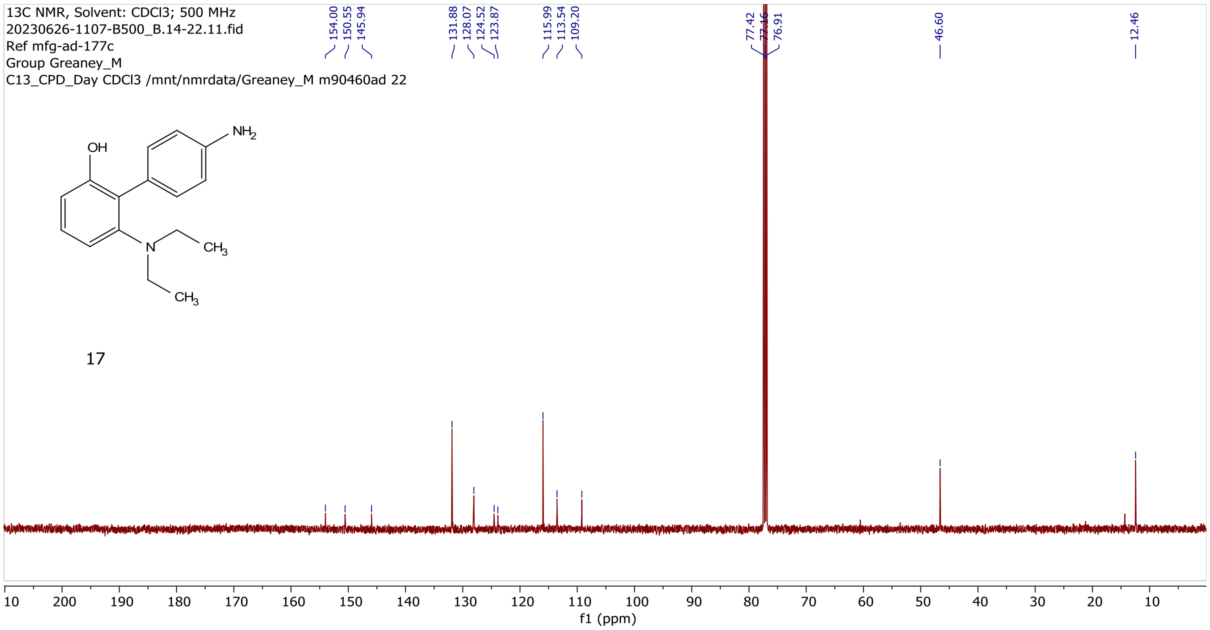
13C NMR, Solvent: CDCl3; 500 MHz
20230626-1107-B500_B.14-22.11.fid
Ref mfg-ad-177c
Group Greaney_M
C13_CPD_Day CDCl3 /mnt/nmrdata/Greaney_M m90460ad 22
154.00
150.55
145.94
131.88
128.07
124.52
123.87
115.99
113.54
109.20
77.42
77.16
76.91
46.60
12.46
OH
NH2
N
CH3
CH3
17
f1 (ppm)

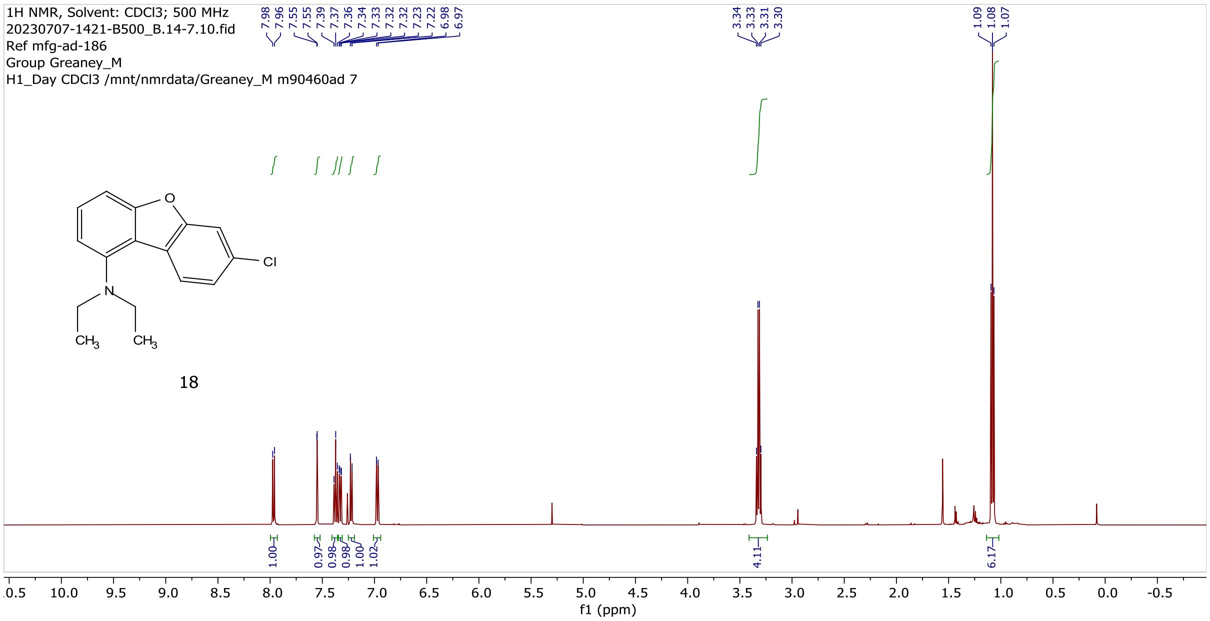
1H NMR, Solvent: CDCl3; 500 MHz
20230707-1421-B500_B.14-7.10.fid
Ref mfg-ad-186
Group Greaney_M
H1_Day CDCl3 /mnt/nmrdata/Greaney_M m90460ad 7
7.98
7.96
7.55
7.55
7.39
7.37
7.36
7.34
7.33
7.32
7.32
7.23
7.22
6.98
6.97
3.34
3.33
3.31
3.30
1.09
1.08
1.07
O
Cl
N
CH3
CH3
18
1.00
0.97
0.98
0.98
1.00
1.02
4.11
6.17
f1 (ppm)

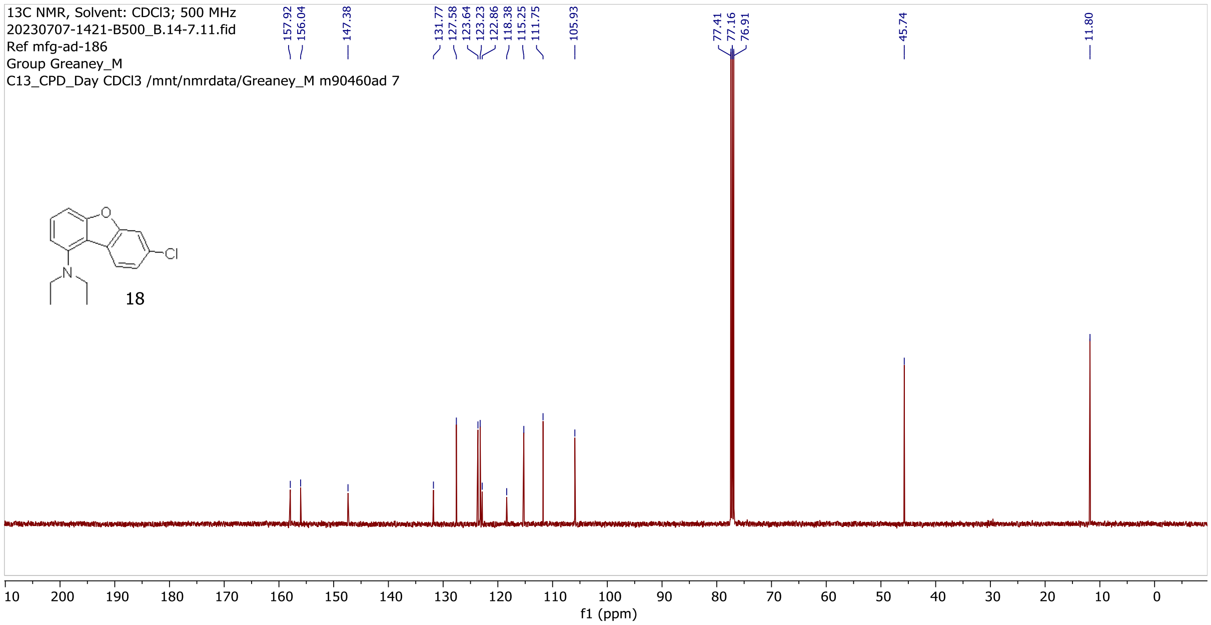

7. References

1. Yoshida, S.; Shimomori, K.; Nonaka, T.; Hosoya, T., Facile Synthesis of Diverse Multisubstituted ortho-Silylaryl Triflates via C–H Borylation. *Chem. Lett.* **2015,** *44*, 1324-1326.
2. Bronner, S. M.; Lee, D.; Bacauanu, V.; Cyr, P., A Novel Cascade Benzyne Nucleophilic Addition/Fries Rearrangement for Entry into 2,3-Disubstituted Phenols. *Synlett* **2017,** *28*, 799-804.
3. Xu, H.; He, J.; Shi, J.; Tan, L.; Qiu, D.; Luo, X.; Li, Y., Domino Aryne Annulation via a Nucleophilic–Ene Process. *J. Am. Chem. Soc.* **2018,** *140*, 3555-3559.
4. Sierakowski, A. F., A convenient synthesis of two dibenzofurans. *Aust. J. Chem.* **1983,** *36*, 1281-1283.
